# Supplementary material for: Effects of Substituents on the Photophysical/Photobiological Properties of Mono-Substituted Corroles
Source: Molecules. 2023 Feb 1;28(3):1385. doi: 10.3390/molecules28031385 (PMC9921392; doi:10.3390/molecules28031385)
Supplement: Supplementary file 1 [file molecules-28-01385-s001.zip › molecules-2164048-supplementary.pdf]

Supplementary information

Effects of substituents on the photophysical/photobiological properties of mono-substituted corroles

Vitória Barbosa de Souza[1], Vinícius N. da Rocha[2], Paulo Cesar Piquini[2], Otávio Augusto Chaves[3] and Bernardo A. Iglesias[1,*]

[1]Department of Chemistry, Bioinorganic and Porphyrinoids Materials Lab, Federal University of Santa Maria, 97105-900, Santa Maria, RS, Brazil

[2]Department of Physics, Federal University of Santa Maria, 97105-900, Santa Maria, RS, Brazil

[3]CQC-IMS, Department of Chemistry, University of Coimbra, Rua Larga, 3004-535, Coimbra, Portugal

*Correspondence: bernardopgq@gmail.com; bernardo.iglesias@ufsm.br (B.A.I.)

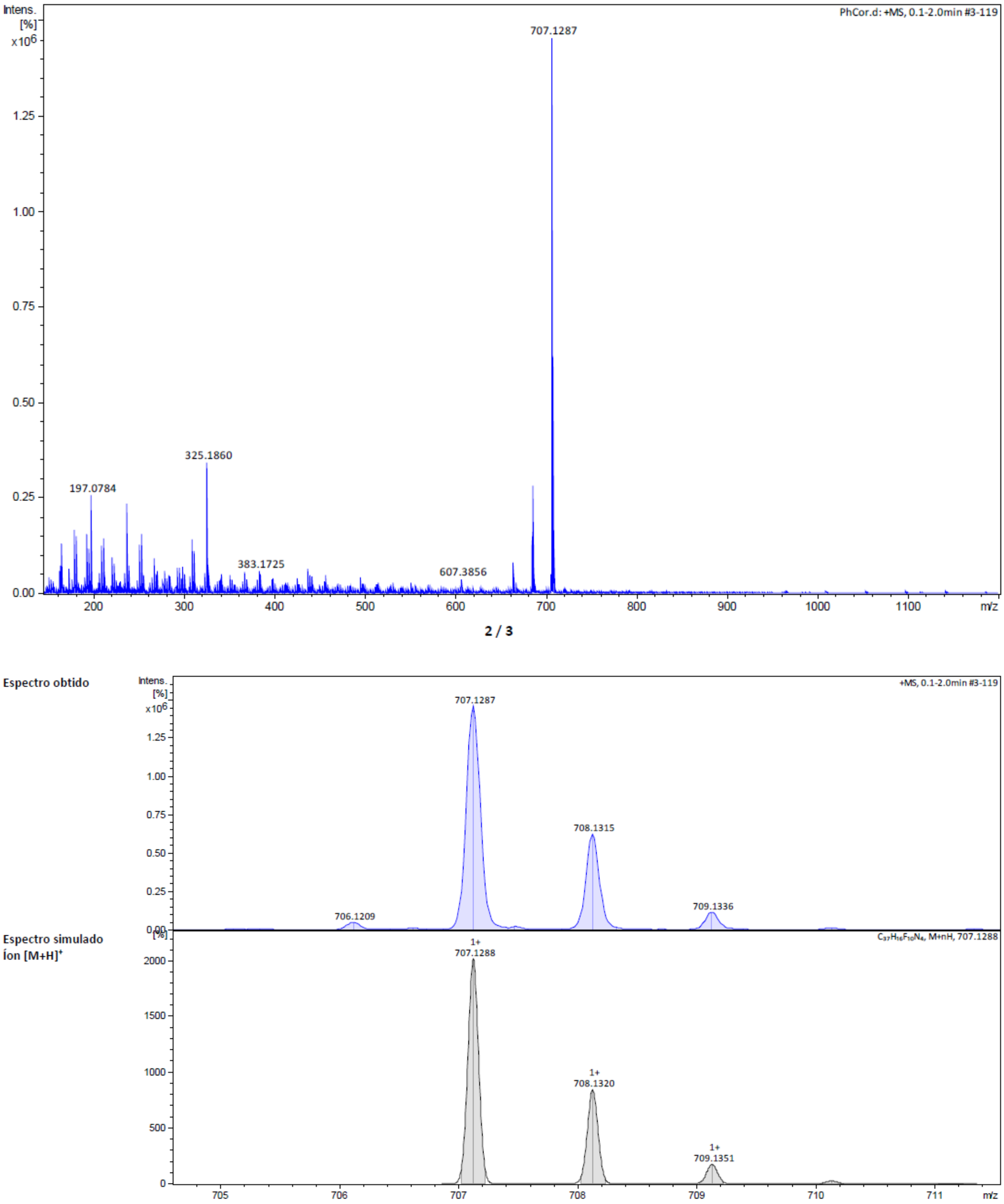

Figure S1. HRMS-ESI(+) mass spectrum of corrole **1**.

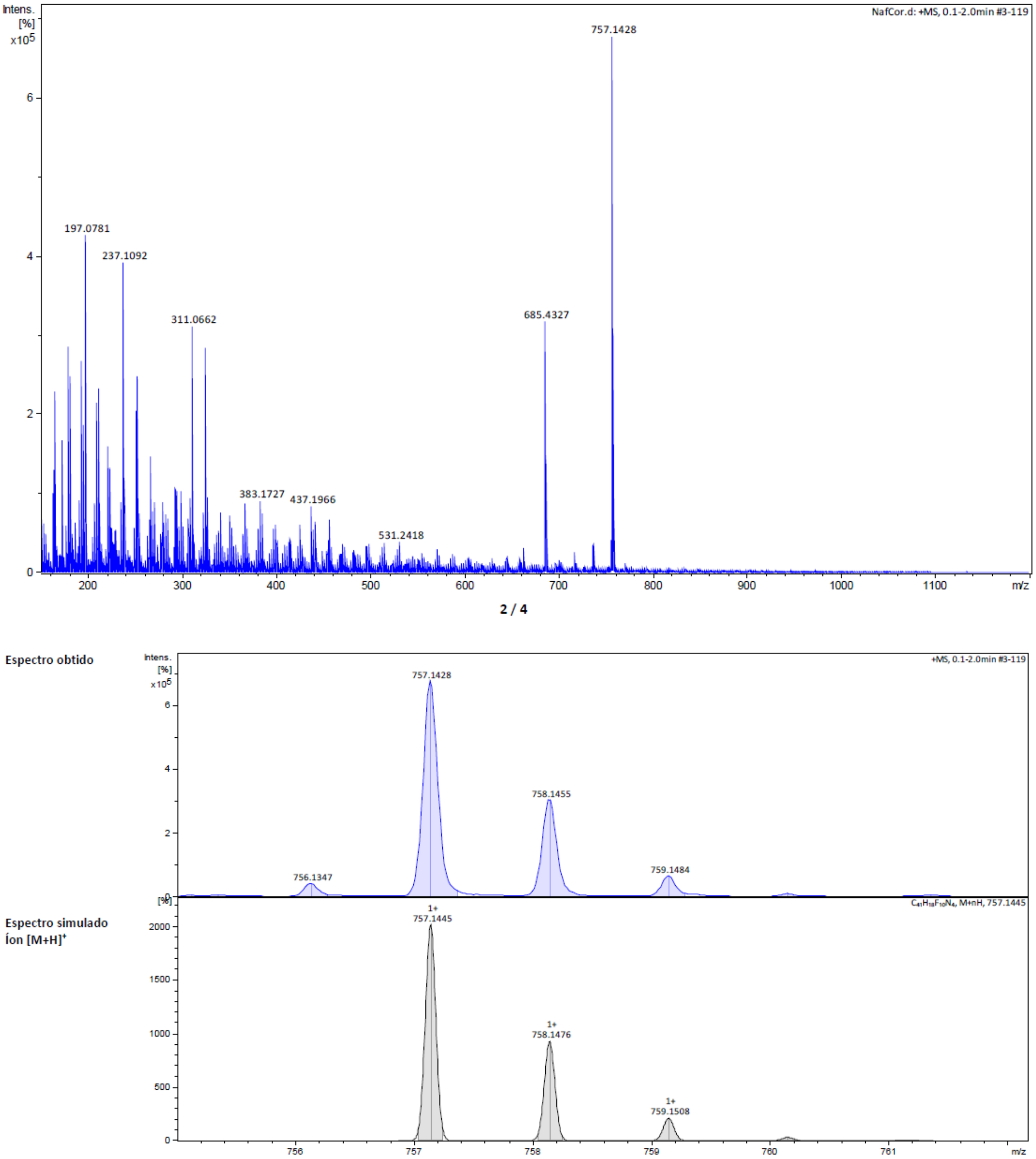

Figure S2. HRMS-ESI(+) mass spectrum of corrole **2**.

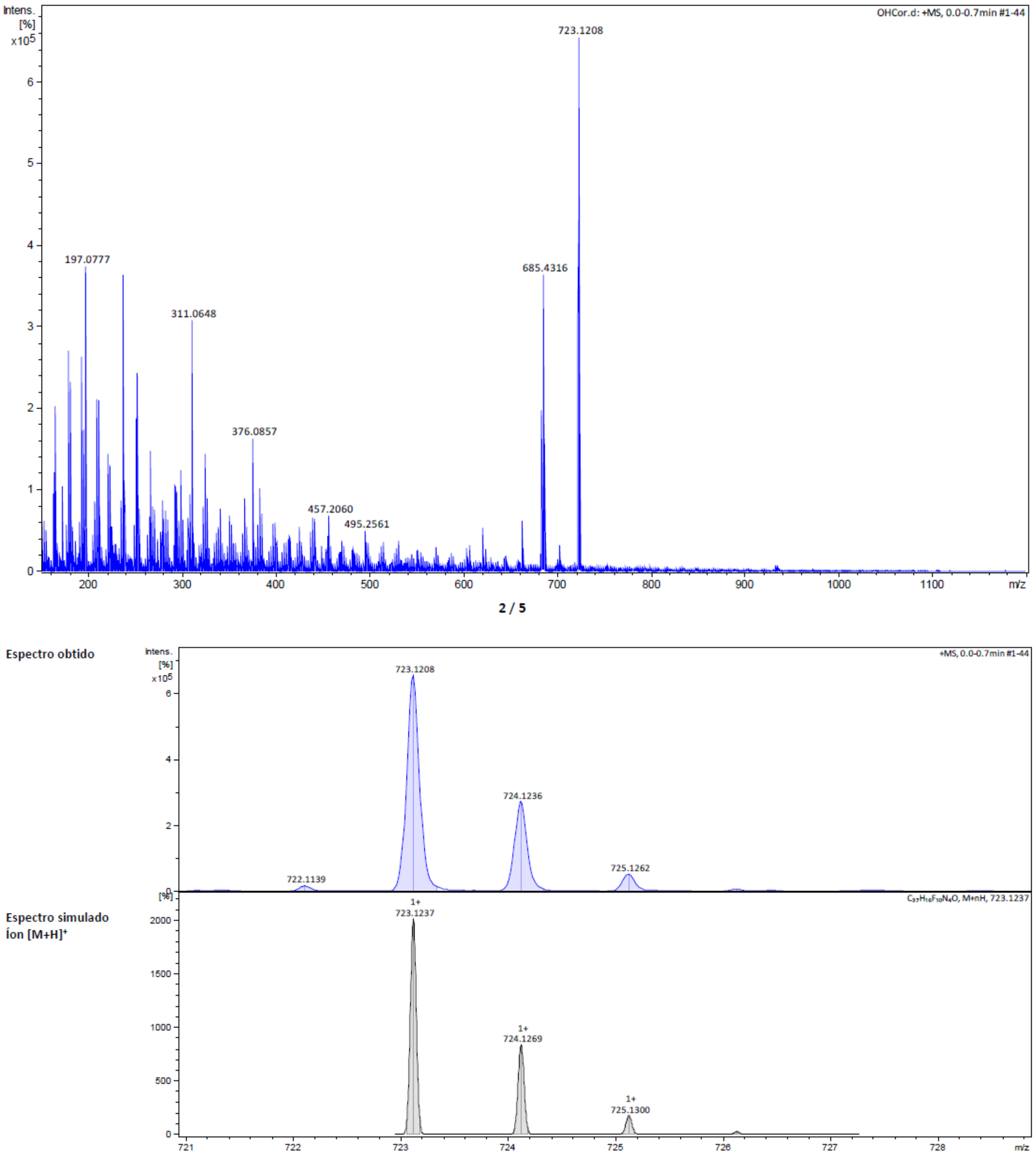

Figure S3. HRMS-ESI(+) mass spectrum of corrole **3**.

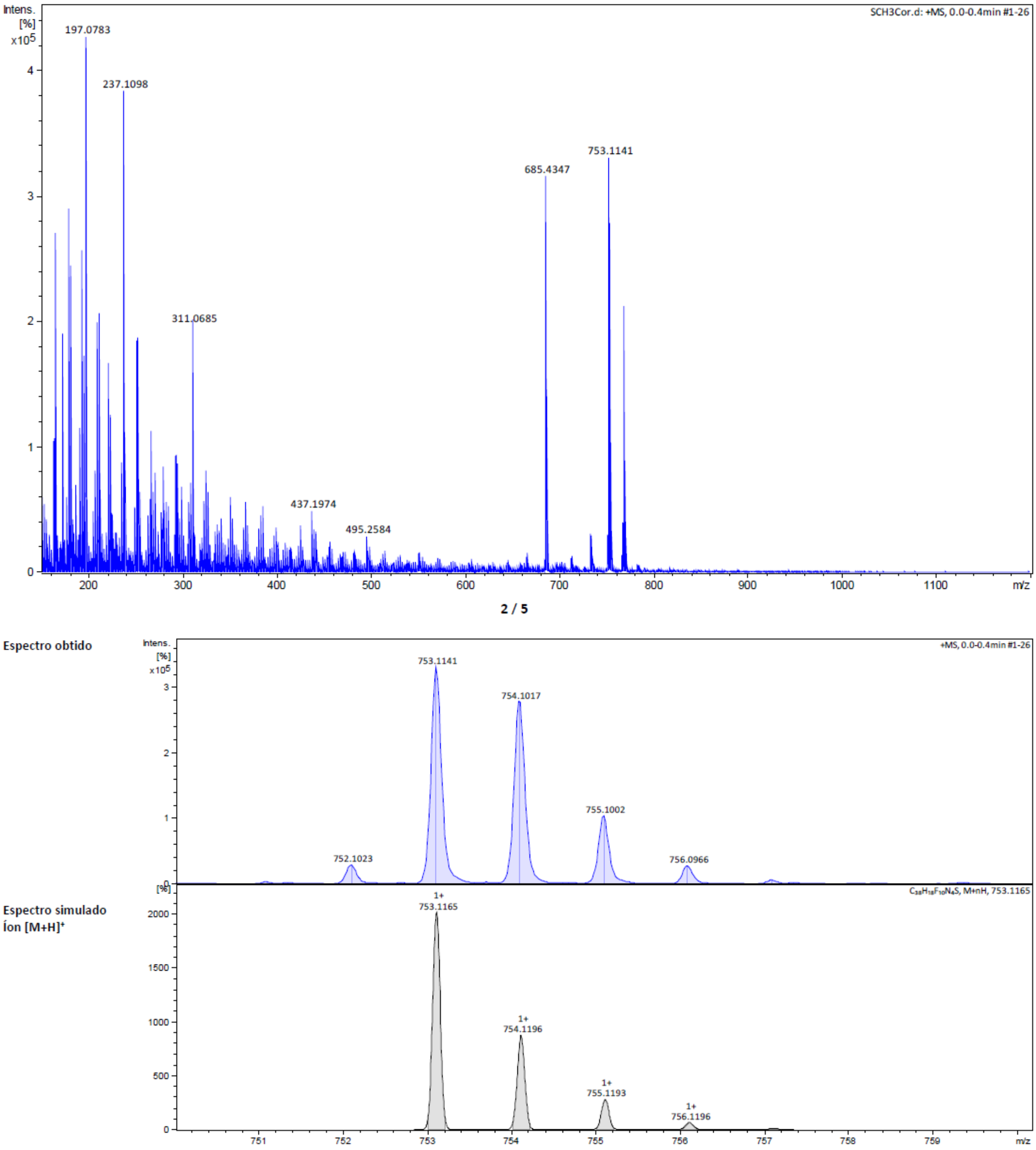

Figure S4. HRMS-ESI(+) mass spectrum of corrole **4**.

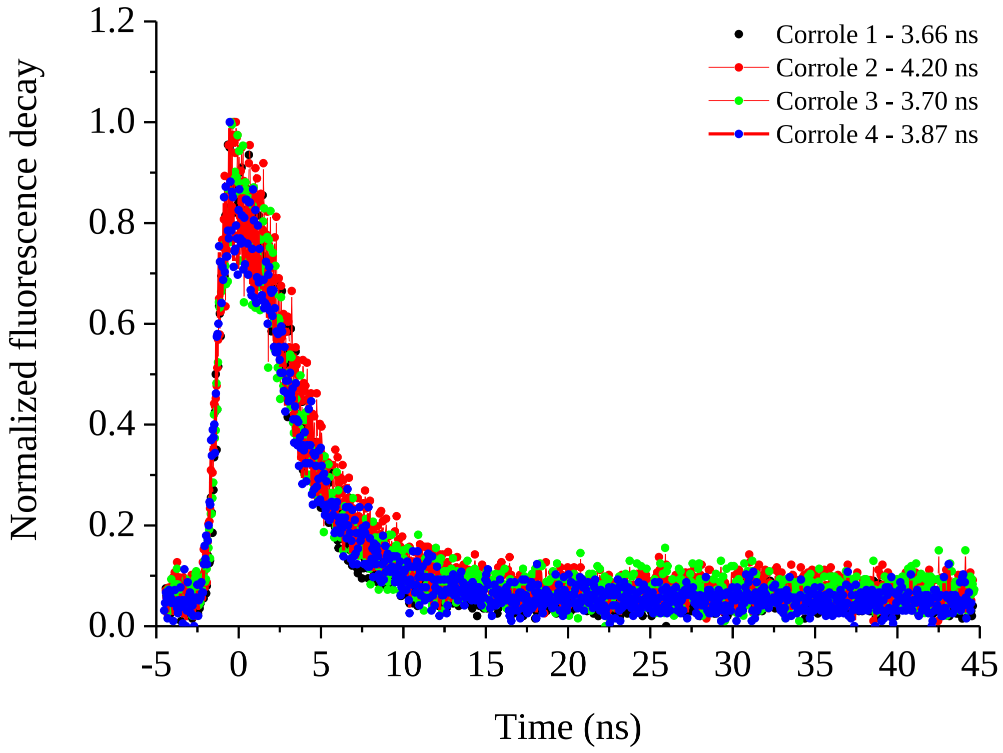

Figure S5. Normalized fluorescence decays of corroles **1-4** in DCM solution.

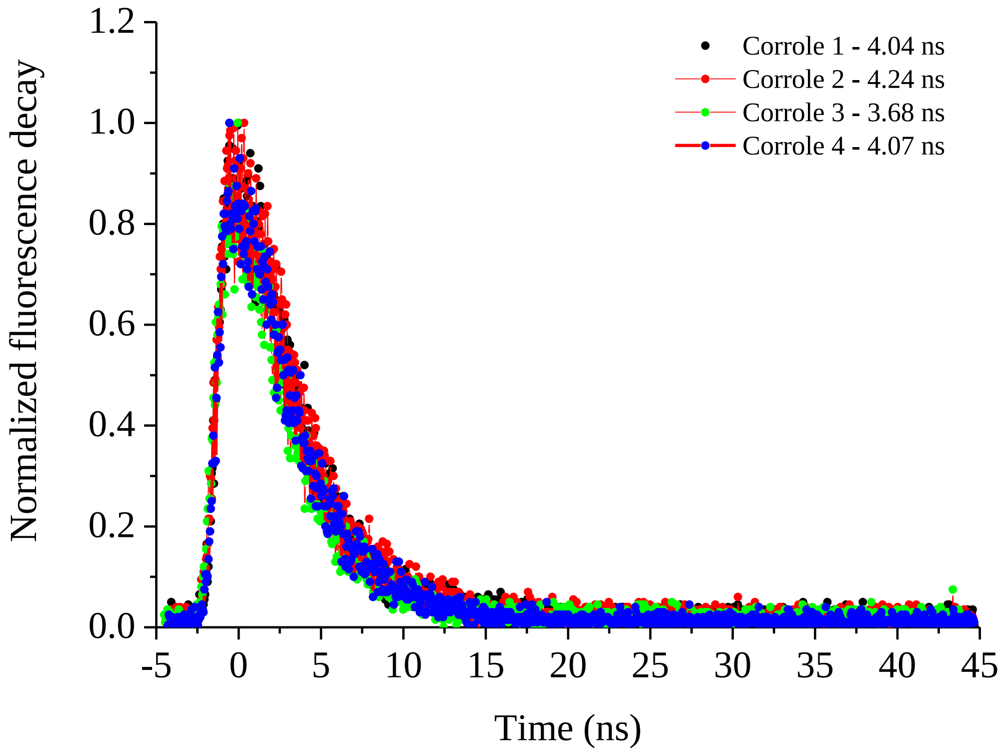

Figure S6. Normalized fluorescence decays of corroles **1-4** in ACN solution.

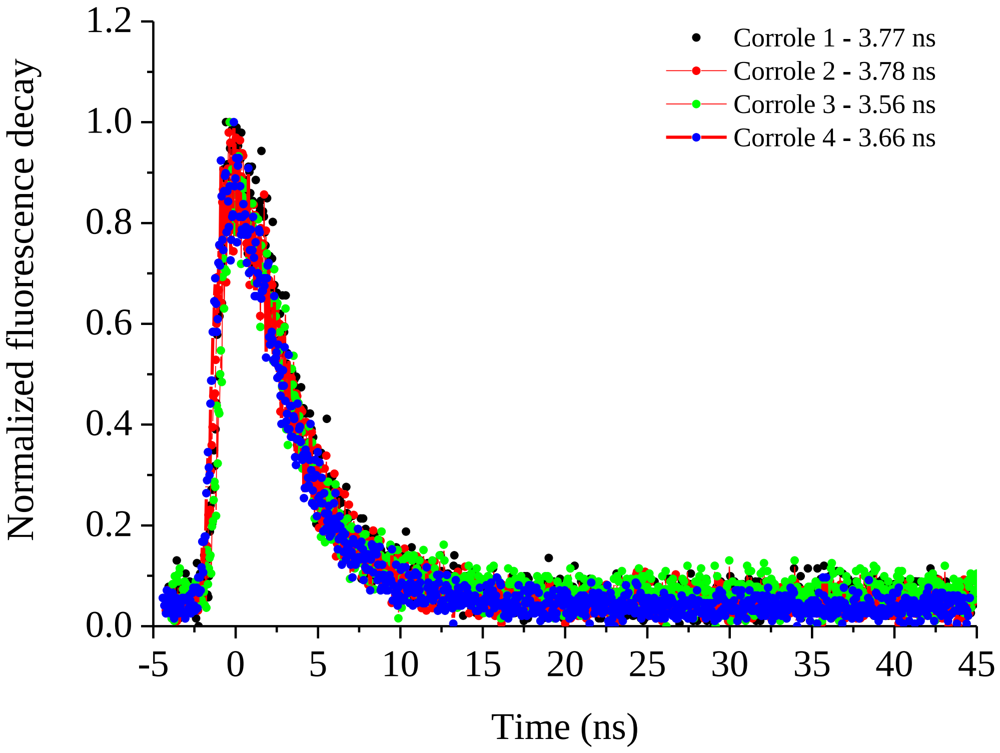

Figure S7. Normalized fluorescence decays of corroles **1-4** in MeOH solution.

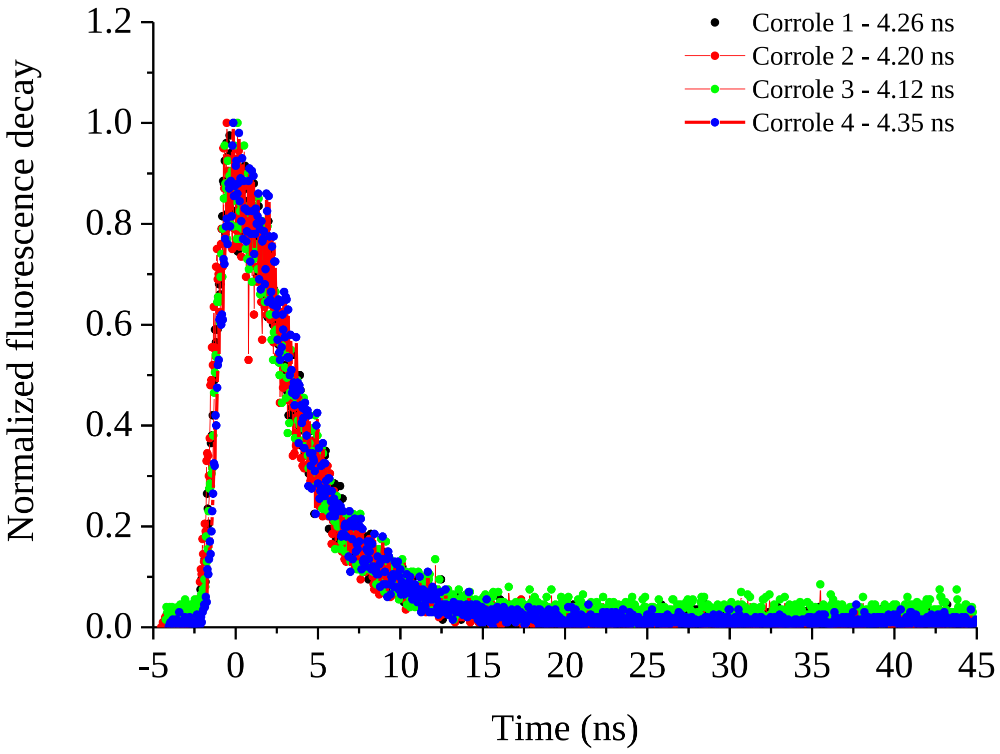

Figure S8. Normalized fluorescence decays of corroles **1-4** in DMSO solution.

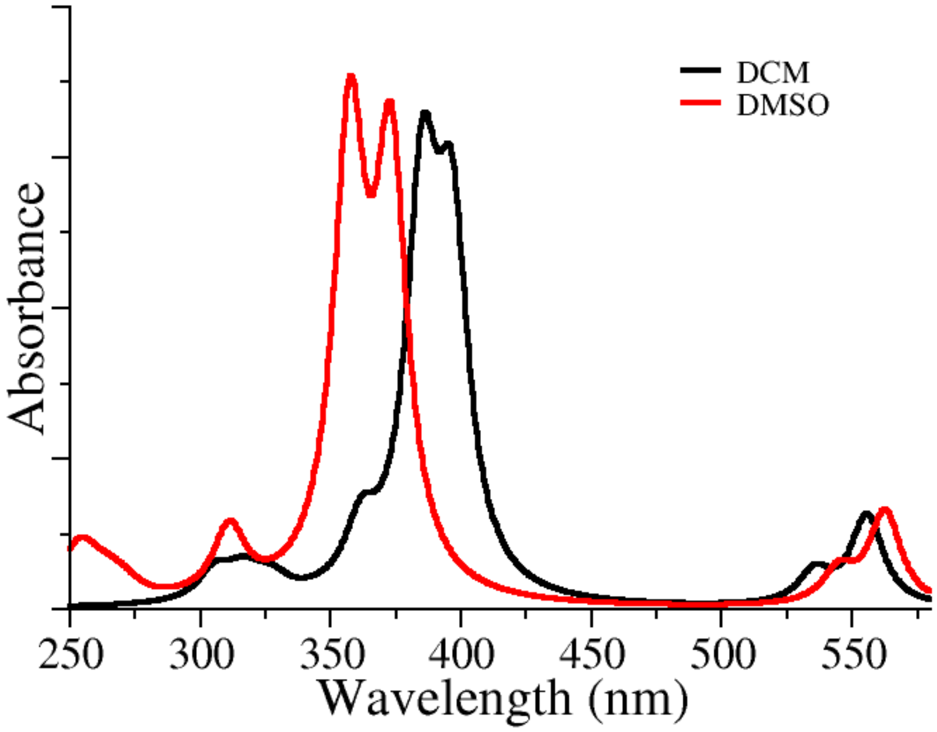

Figure S9. Theoretical optical absorption spectra in DCM and DMSO of corrole **1**.

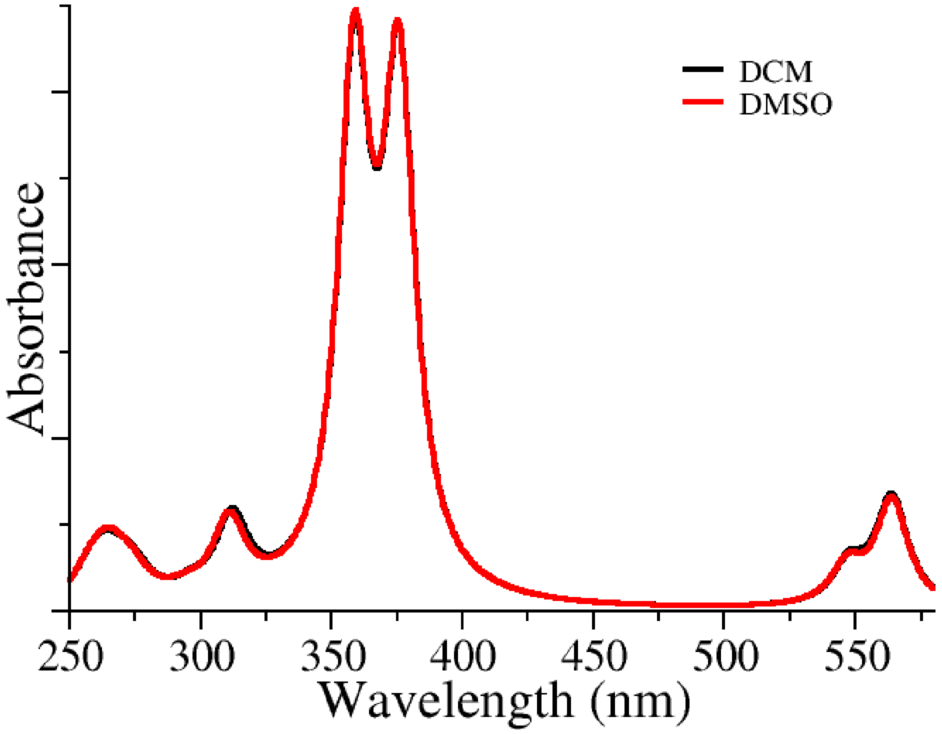

Figure S10. Theoretical optical absorption spectra in DCM and DMSO of corrole **2**.

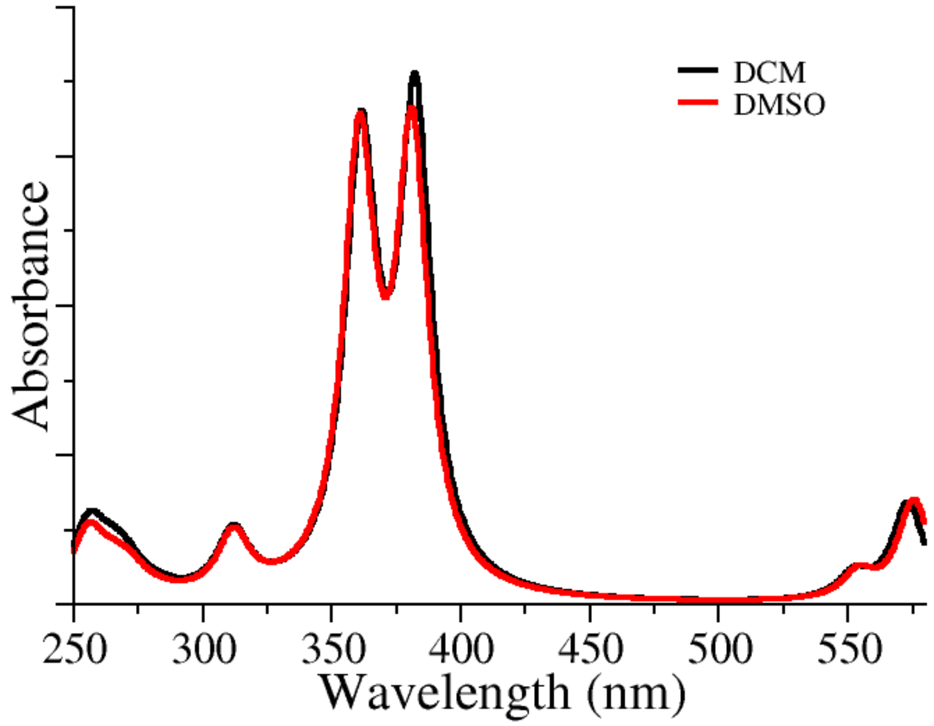

Figure S11. Theoretical optical absorption spectra in DCM and DMSO of corrole **3**.

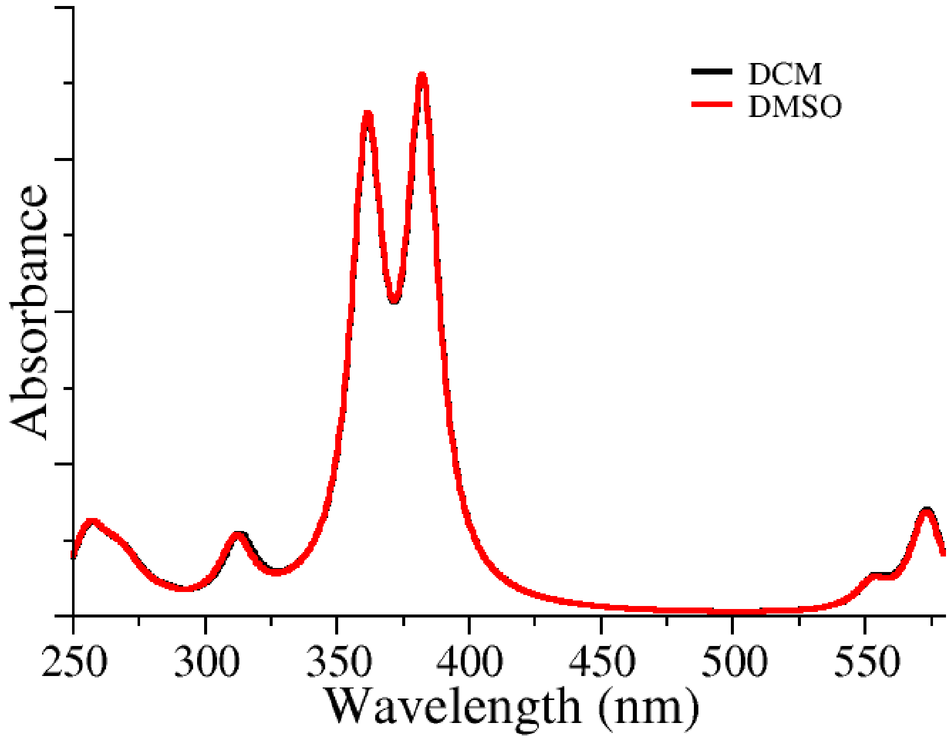

Figure S12. Theoretical optical absorption spectra in DCM and DMSO of corrole **4**.

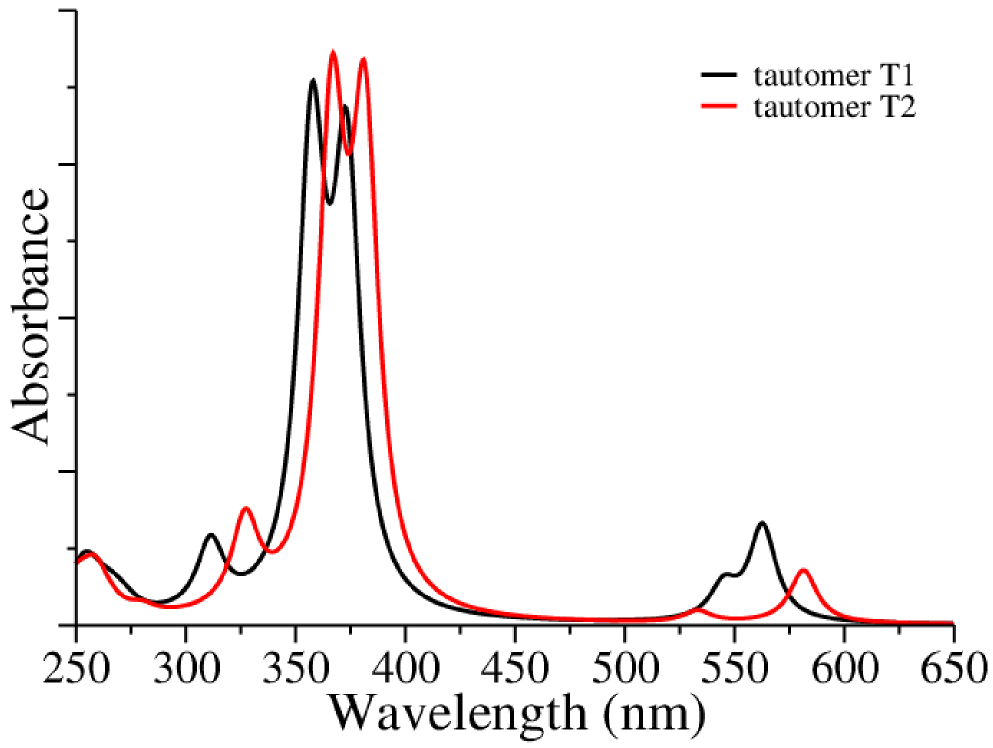

Figure S13. Theoretical optical absorption spectra in DMSO of tautomeric states of corrole **1**.

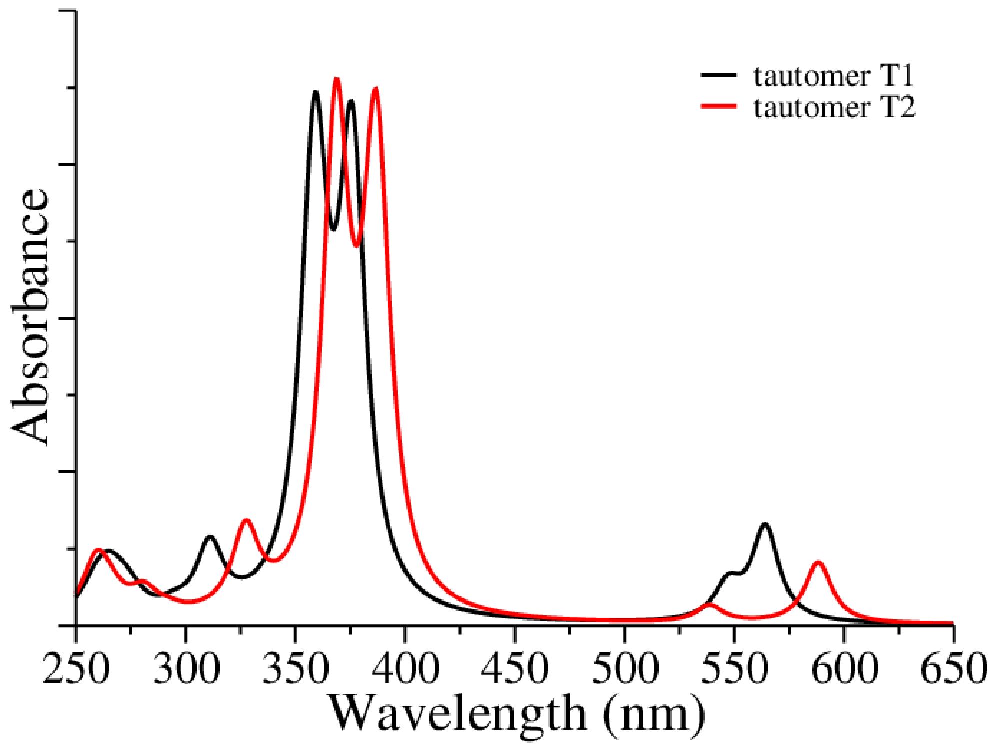

Figure S14. Theoretical optical absorption spectra in DMSO of tautomeric states of corrole **2**.

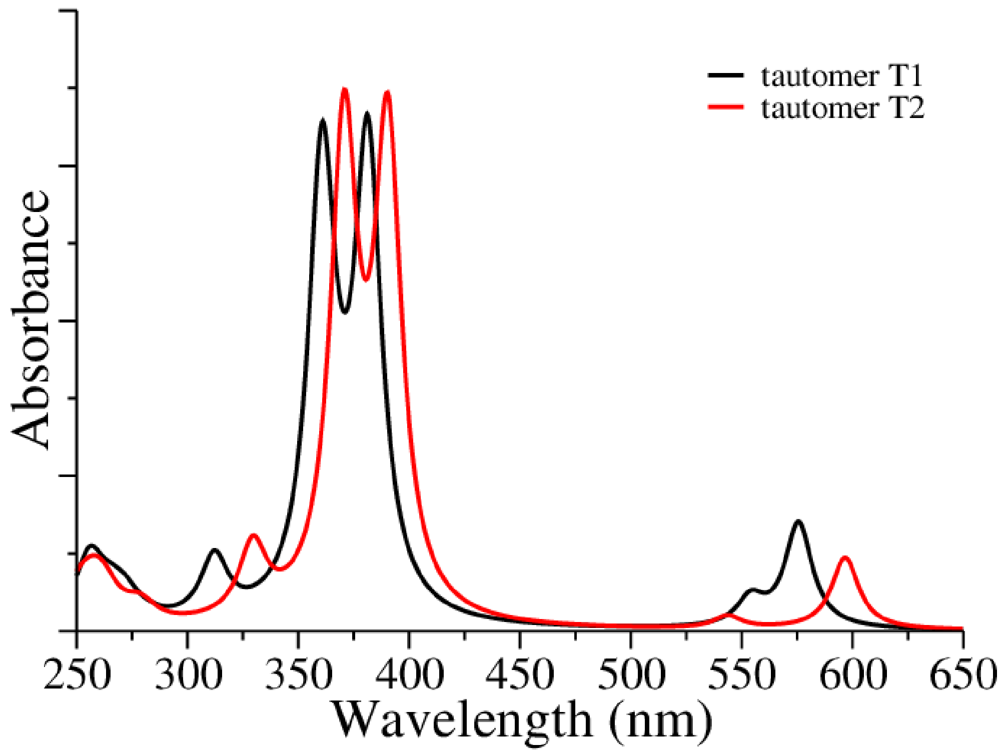

Figure S15. Theoretical optical absorption spectra in DMSO of tautomeric states of corrole **3**.

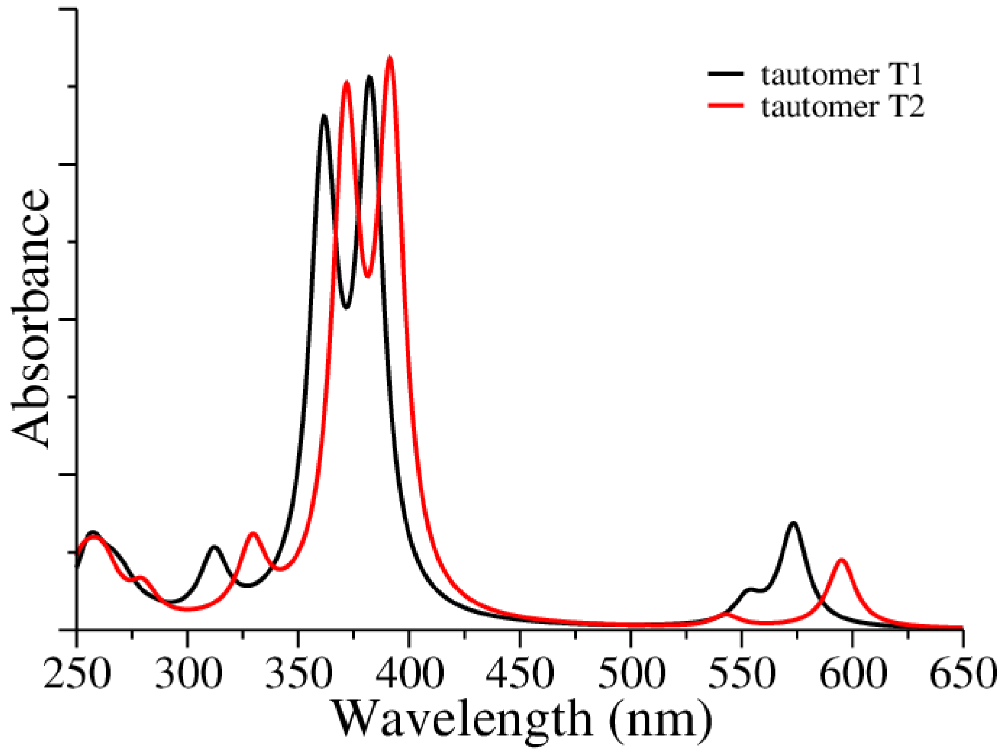

Figure S16. Theoretical optical absorption spectra in DMSO of tautomeric states of corrole **4**.

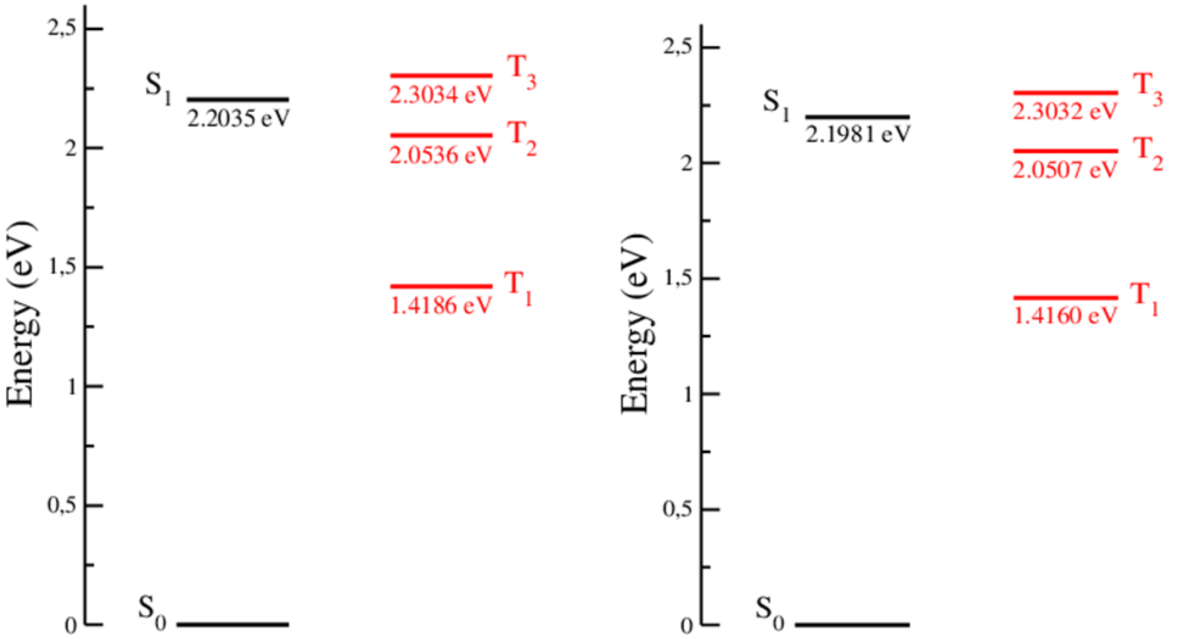

Figure S17. Total energies of the S1 and T1, T2 and T3 excited states, relative to the ground state S0 of corroles **1** (left panel), and **2** (right panel).

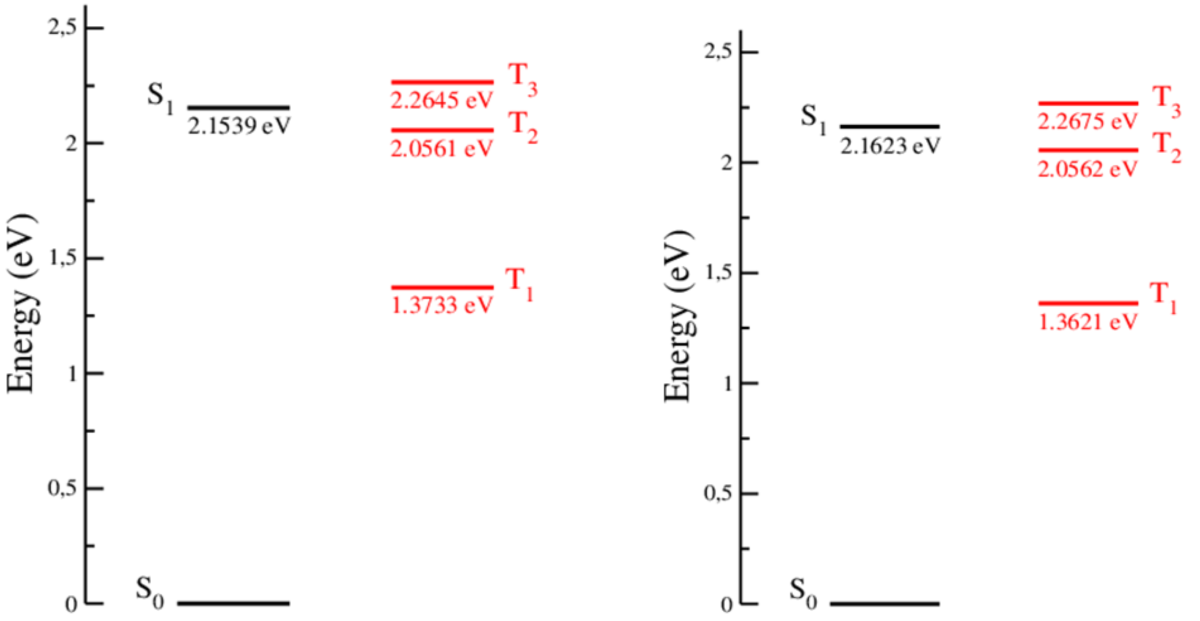

Figure S18. Total energies of the S1 and T1, T2 and T3 excited states, relative to the ground state S0 of corroles **3** (left panel), and **4** (right panel).

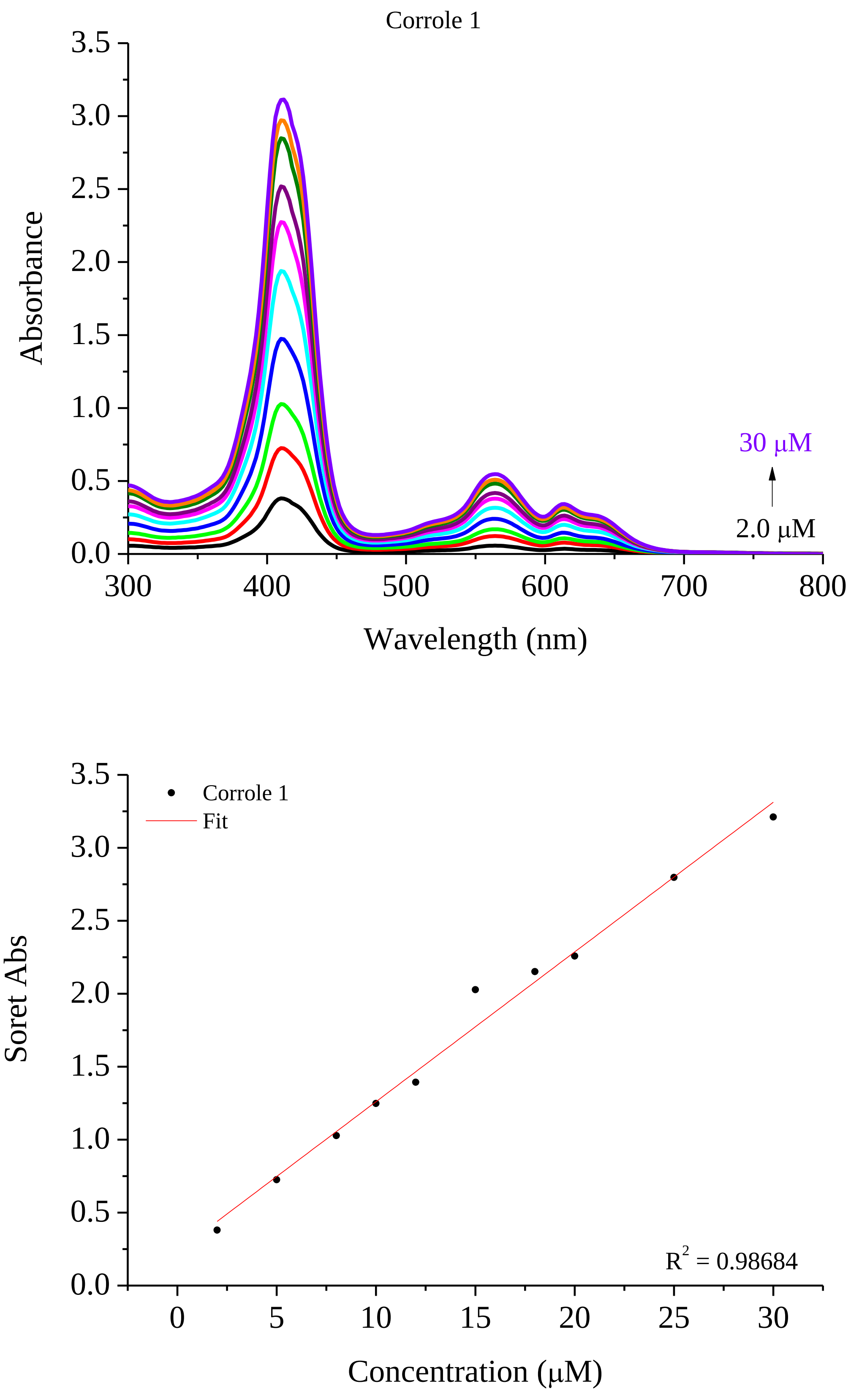

Figure S19. (up) Aggregation behavior of corrole **1** in DCM solution and (down) Abs_{Soret} *versus* concentration plot.

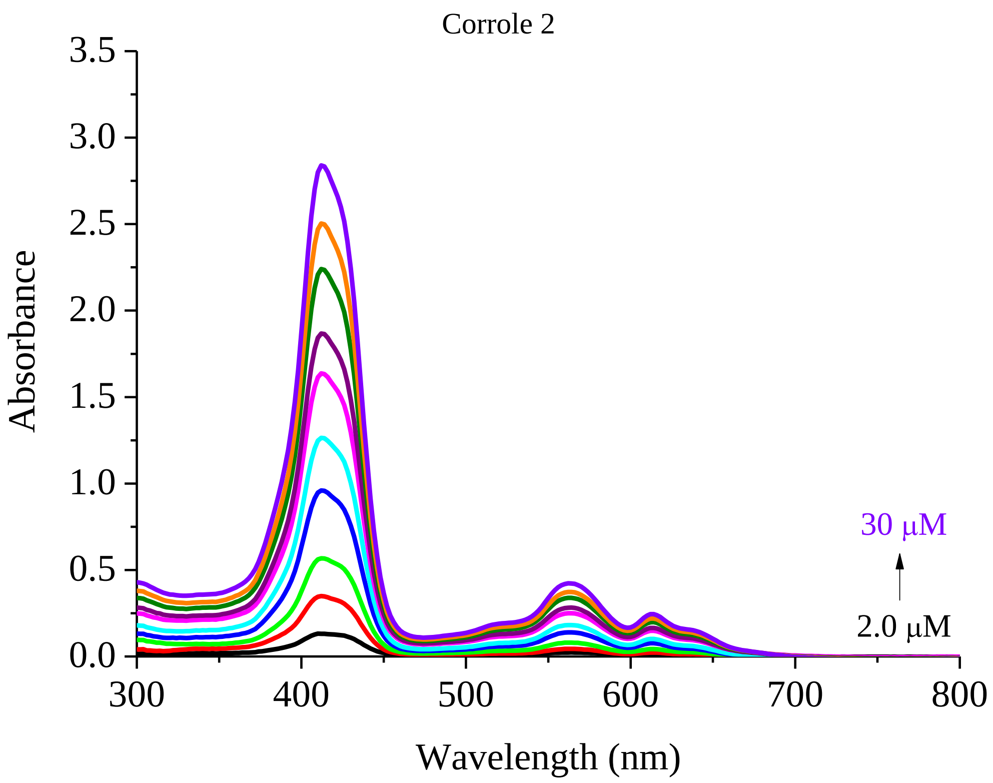

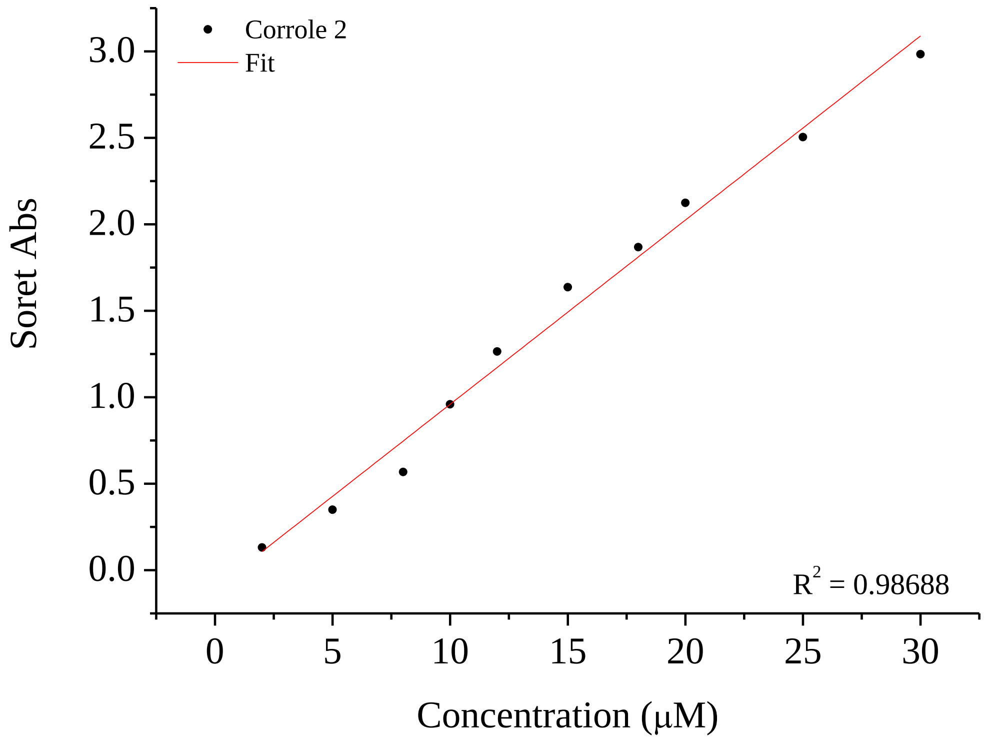

Figure S20. (up) Aggregation behavior of corrole **2** in DCM solution and (down) Abs_{Soret} *versus* concentration plot.

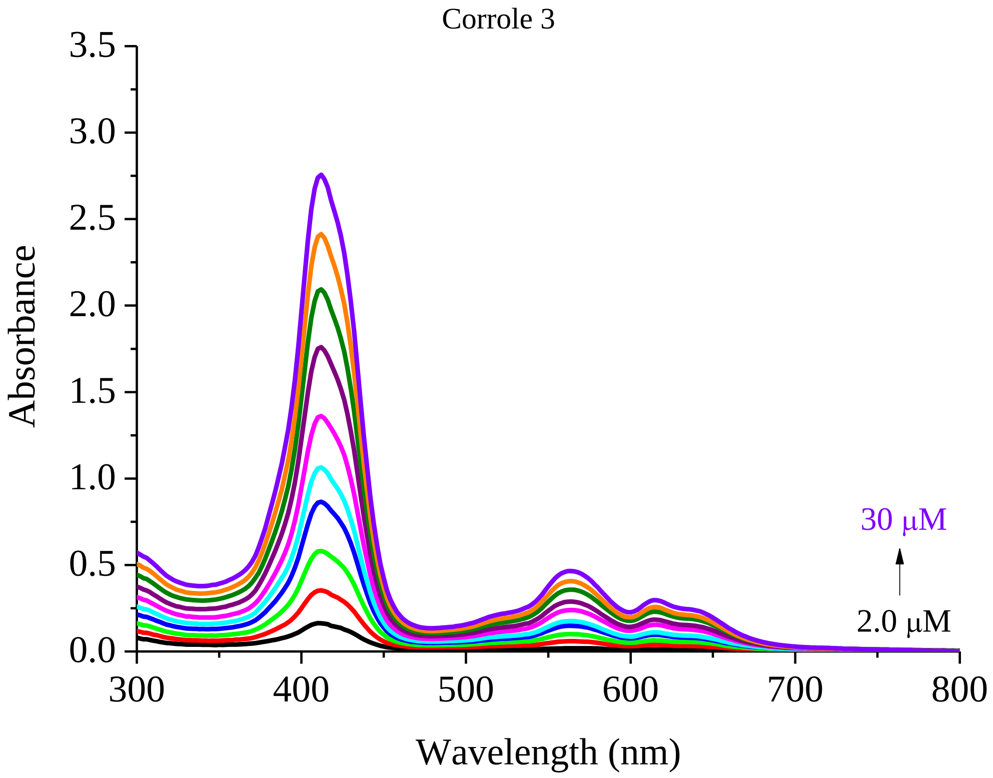

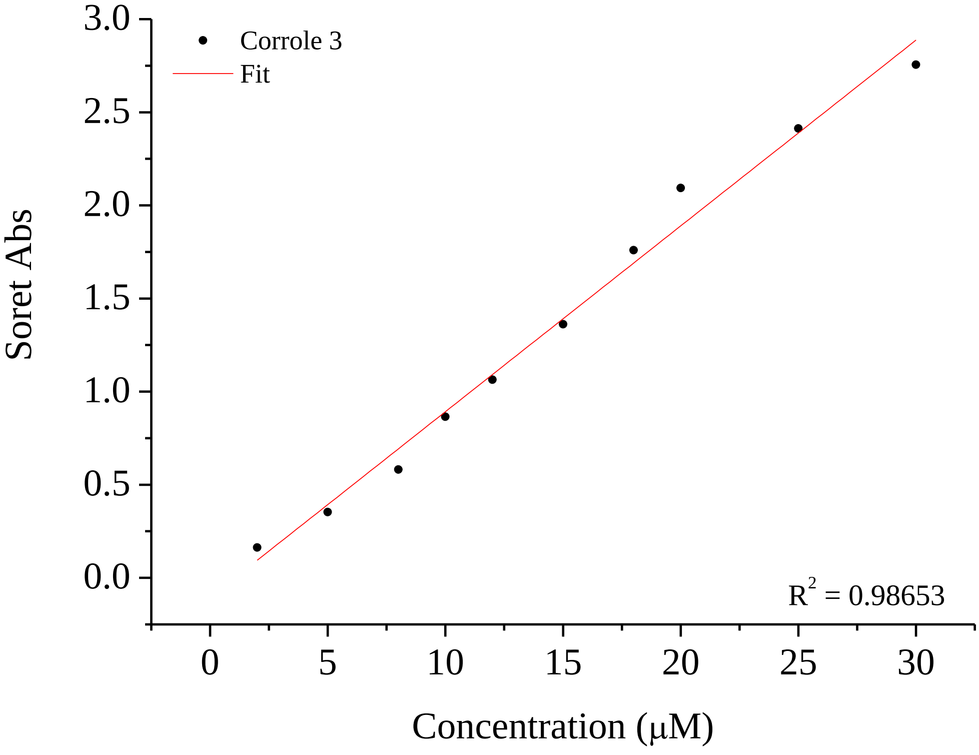

Figure S21. (up) Aggregation behavior of corrole **3** in DCM solution and (down) Abs_{Soret} *versus* concentration plot.

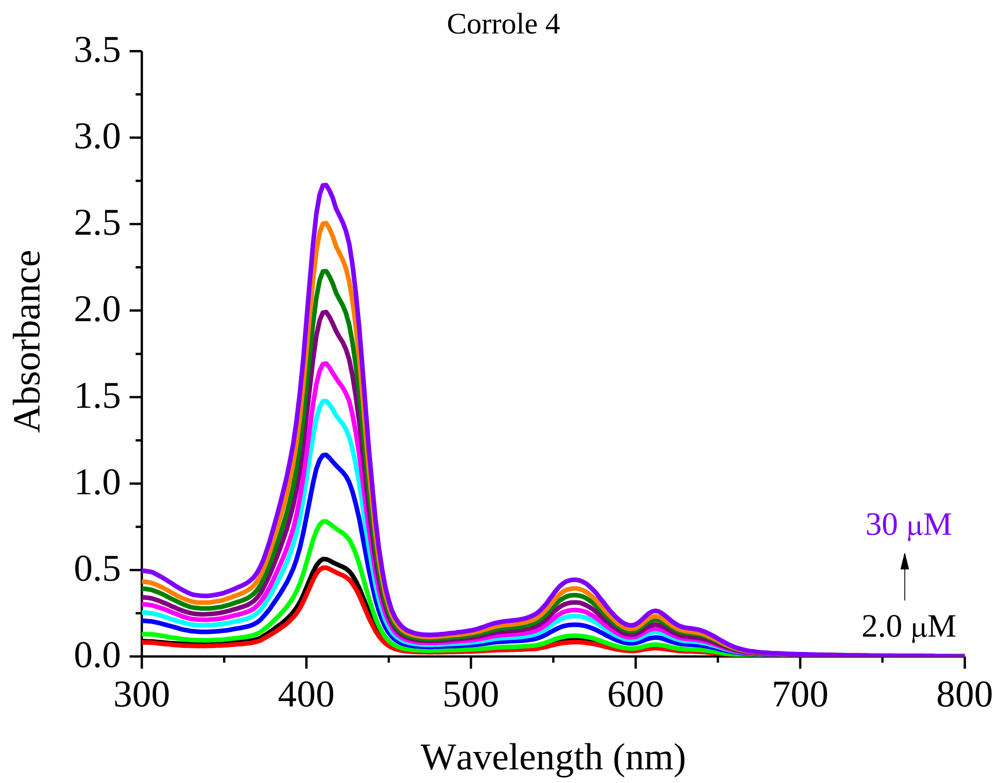

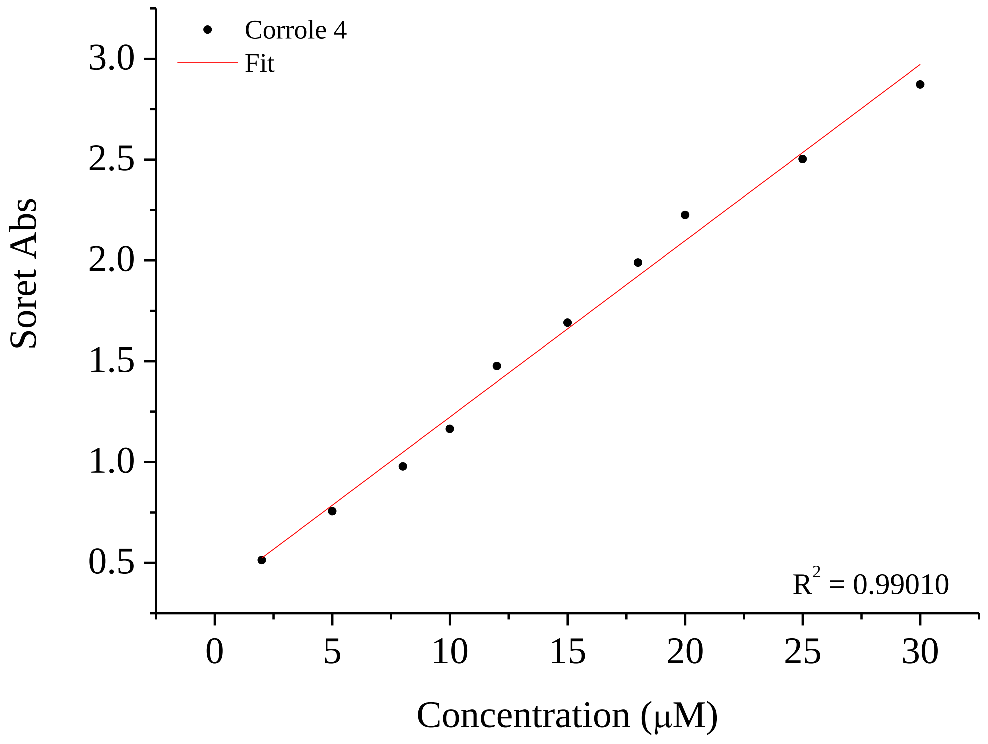

Figure S22. (up) Aggregation behavior of corrole **4** in DCM solution and (down) Abs_{Soret} *versus* concentration plot.

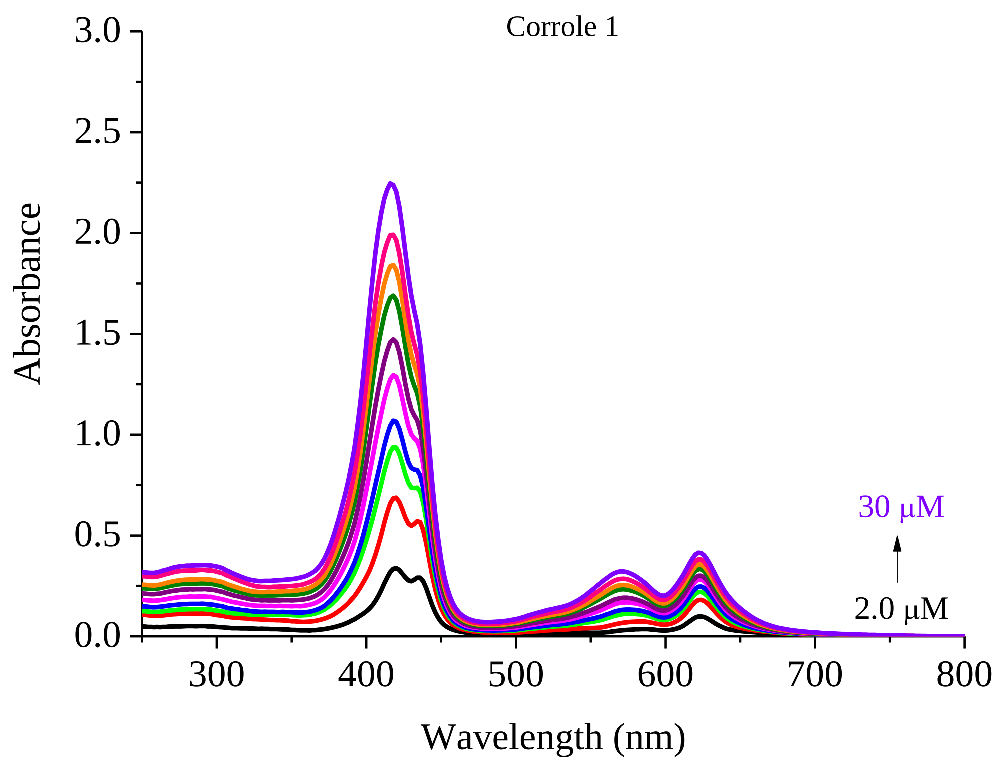

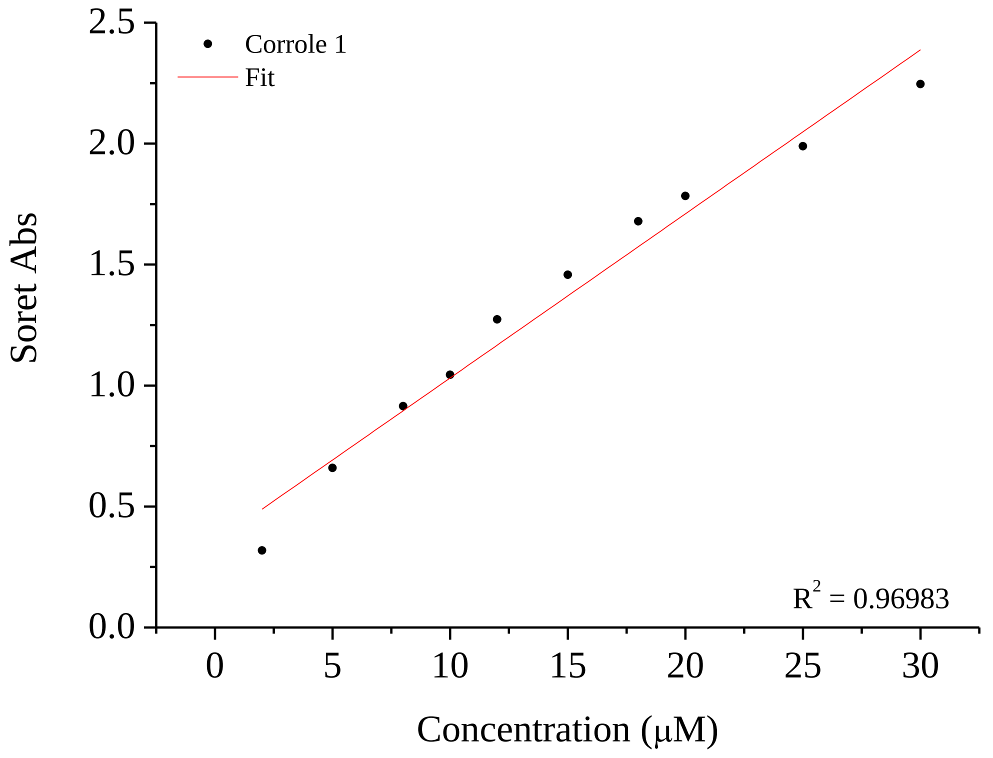

Figure S23. (up) Aggregation behavior of corrole **1** in ACN solution and (down) Abs_{Soret} *versus* concentration plot.

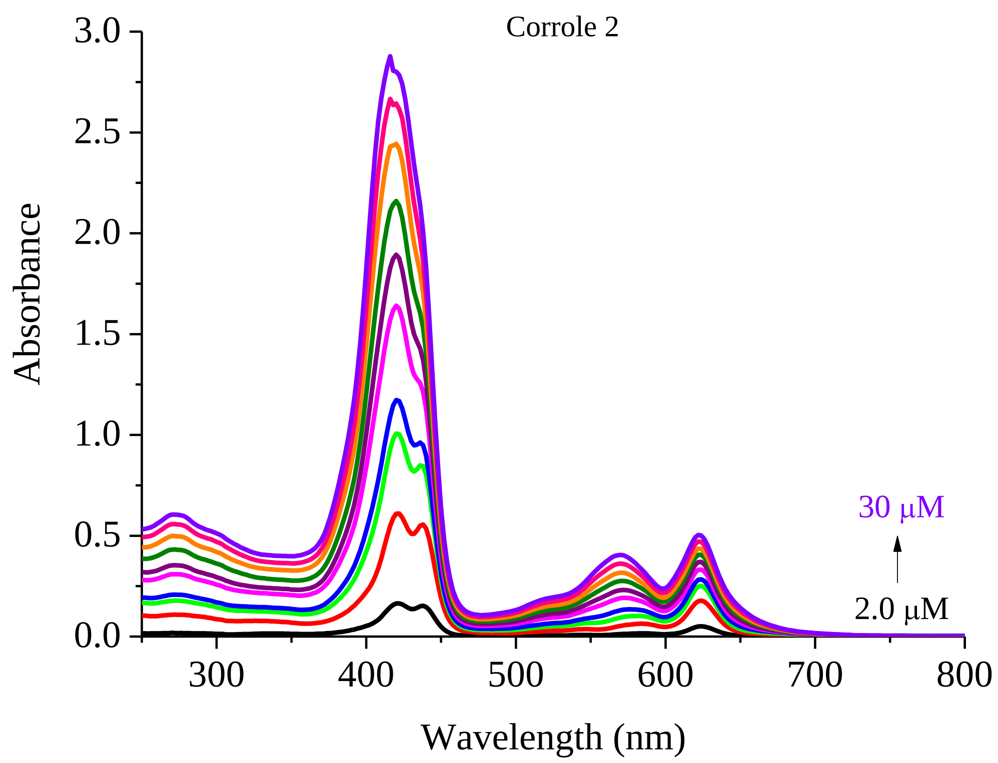

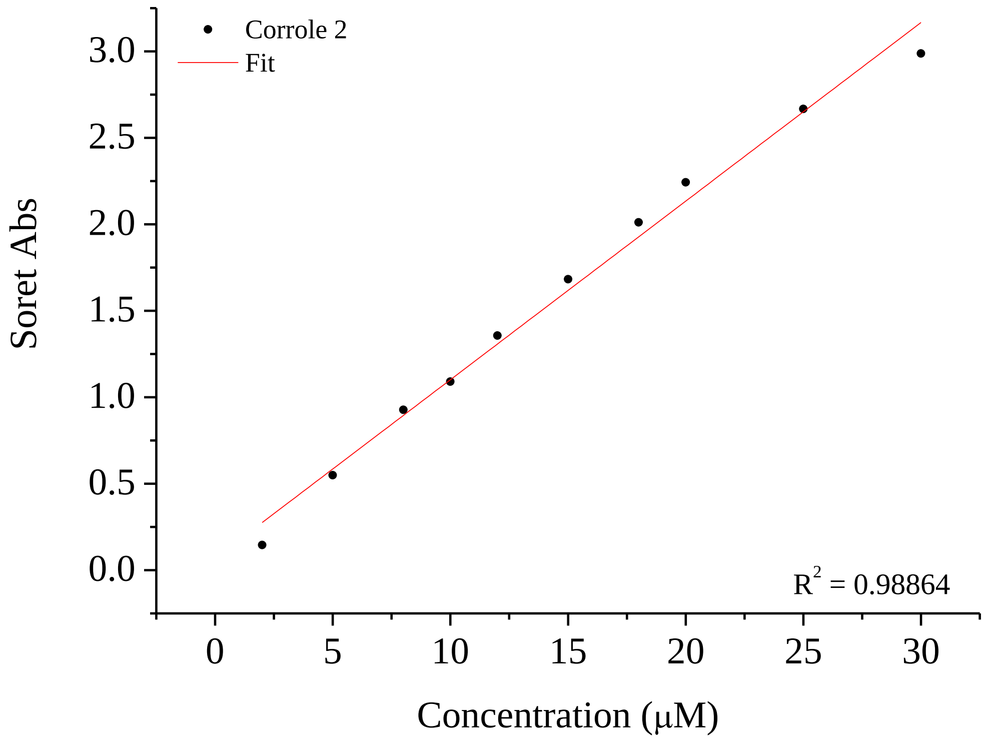

Figure S24. (up) Aggregation behavior of corrole **2** in ACN solution and (down) Abs_{Soret} *versus* concentration plot.

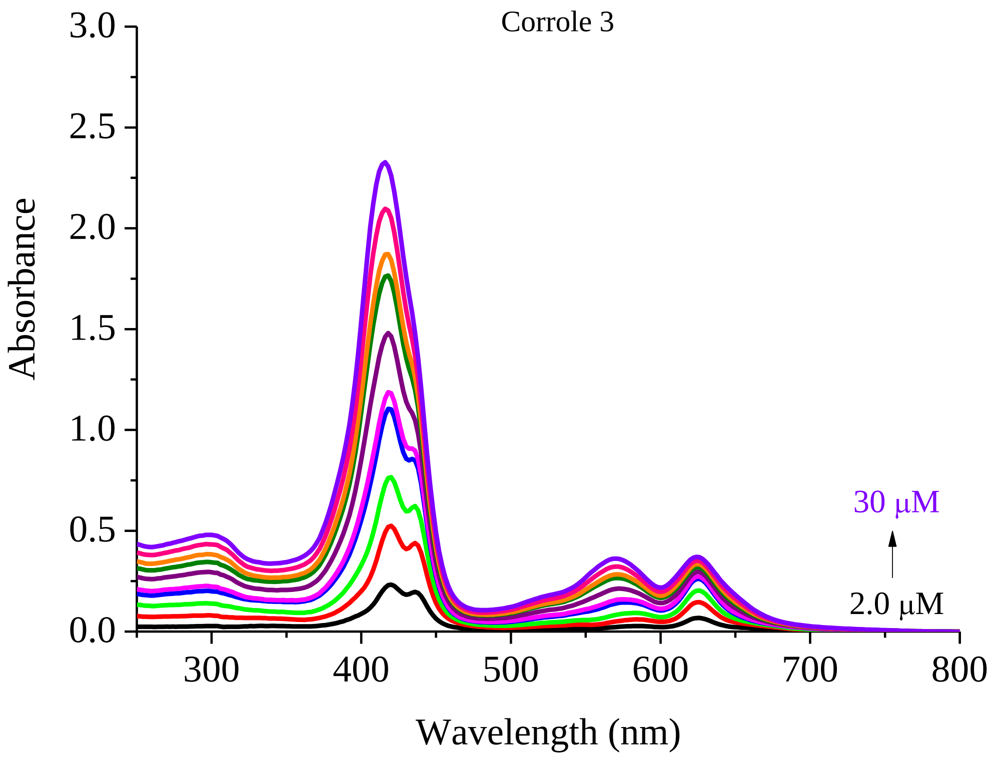

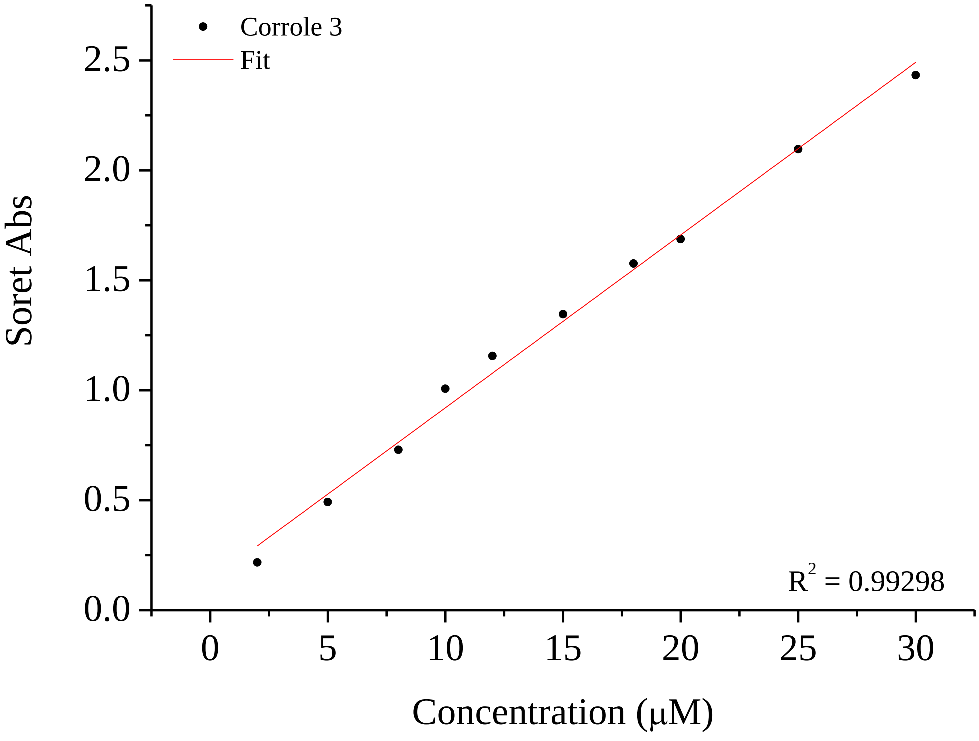

Figure S25. (up) Aggregation behavior of corrole **3** in ACN solution and (down) Abs_{Soret} *versus* concentration plot.

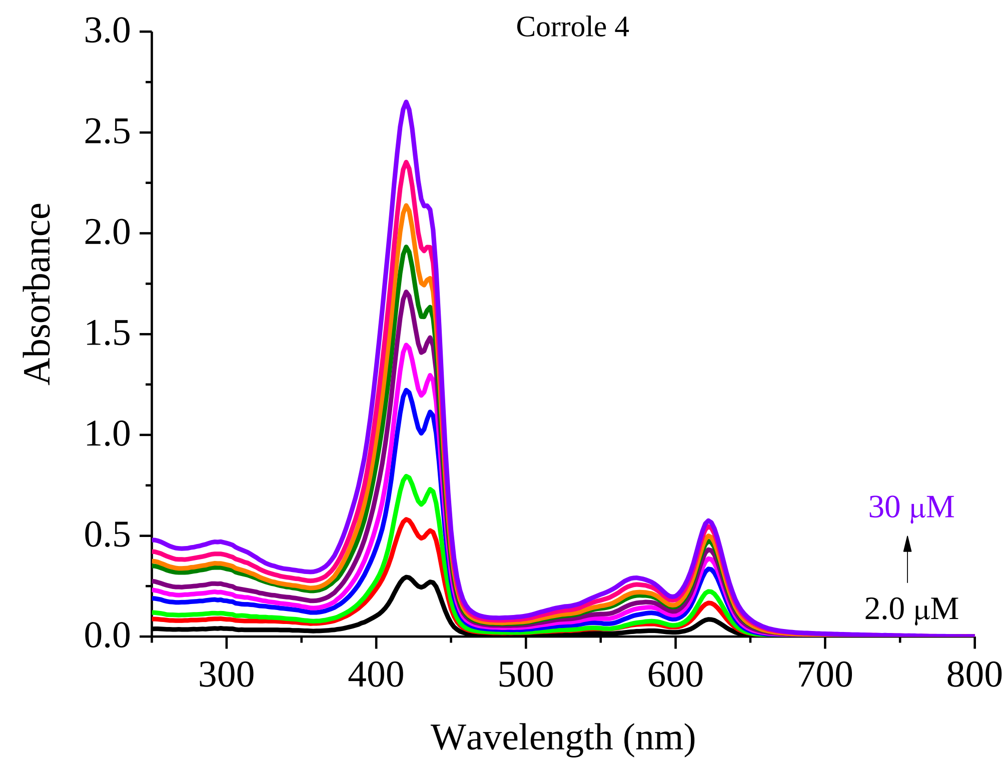

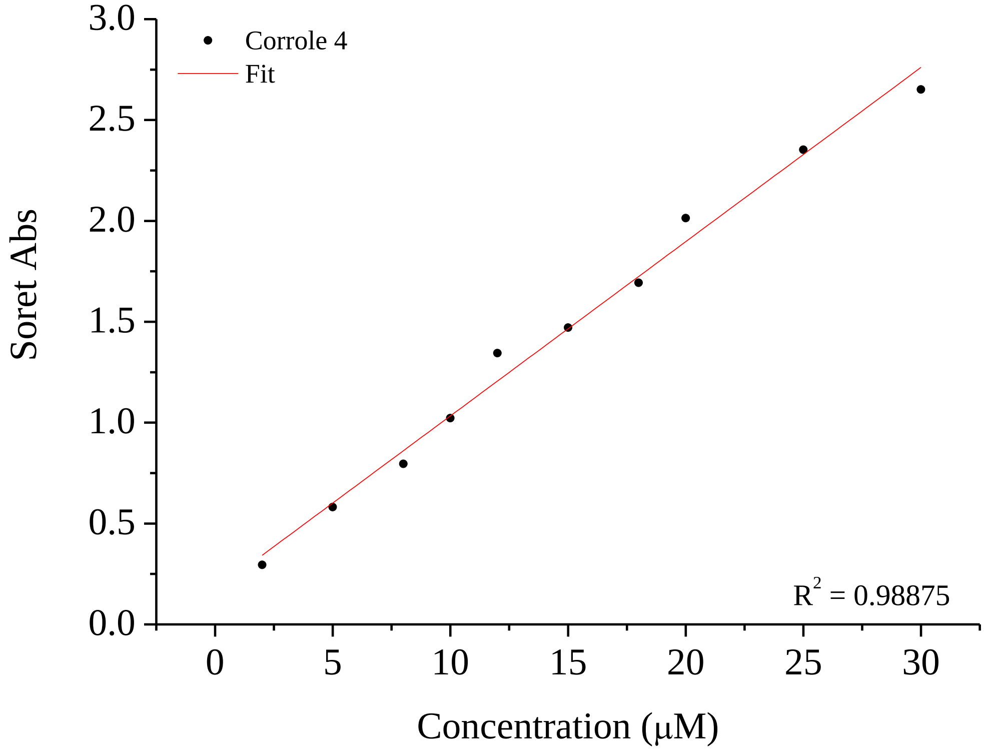

Figure S26. (up) Aggregation behavior of corrole **4** in ACN solution and (down) Abs_{Soret} *versus* concentration plot.

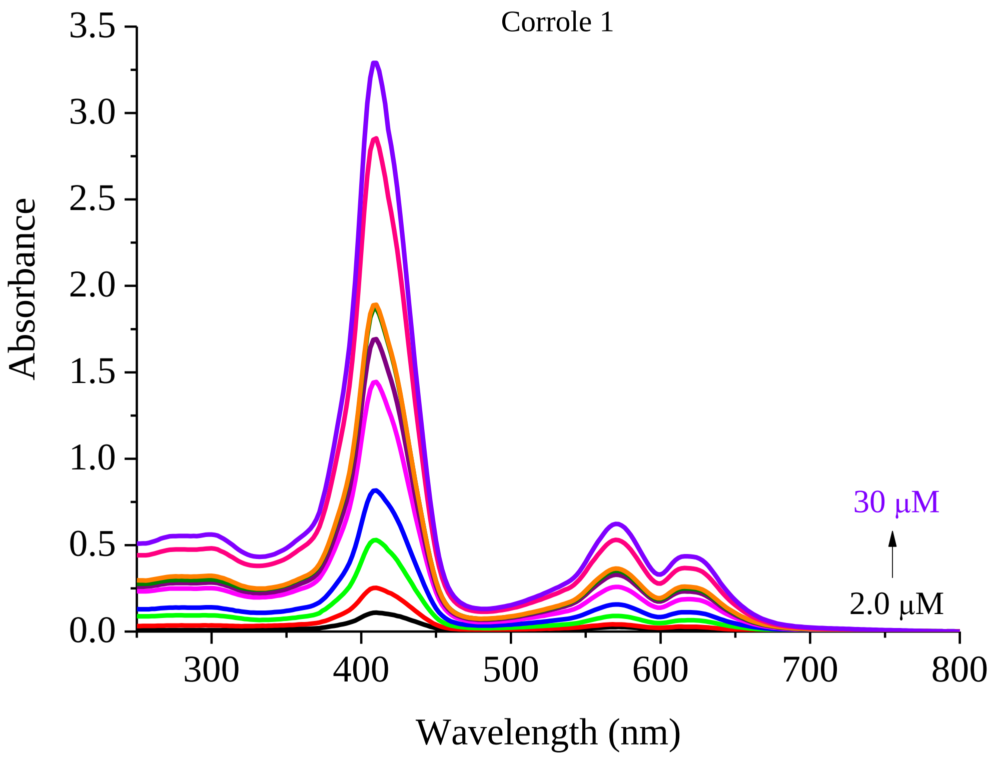

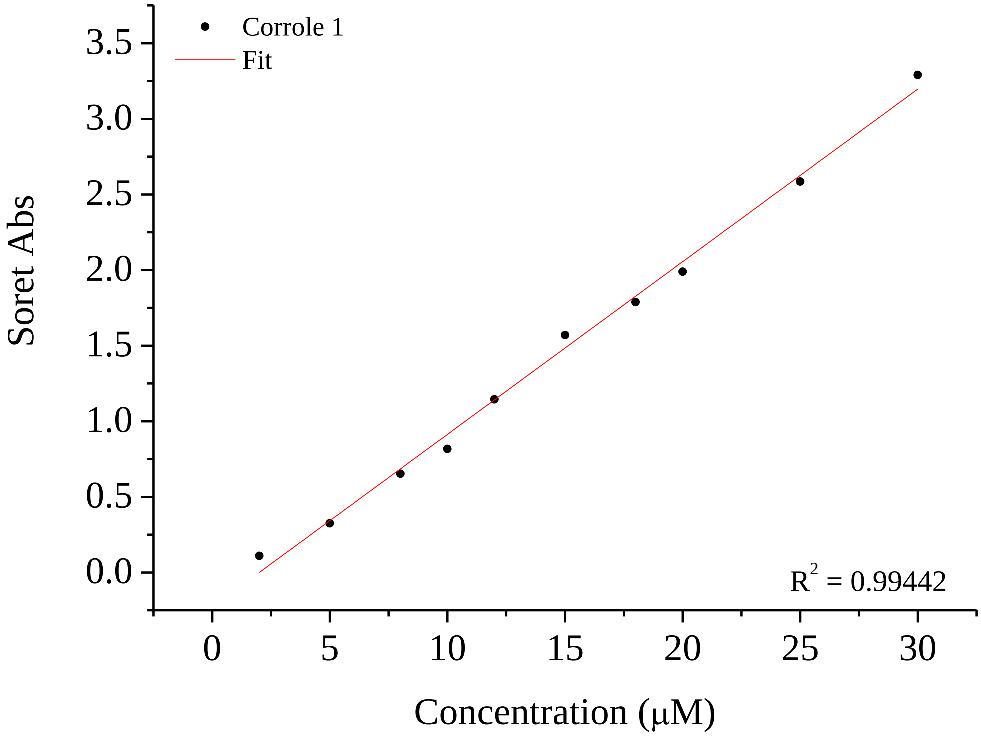

Figure S27. (up) Aggregation behavior of corrole **1** in MeOH solution and (down) Abs_{Soret} *versus* concentration plot.

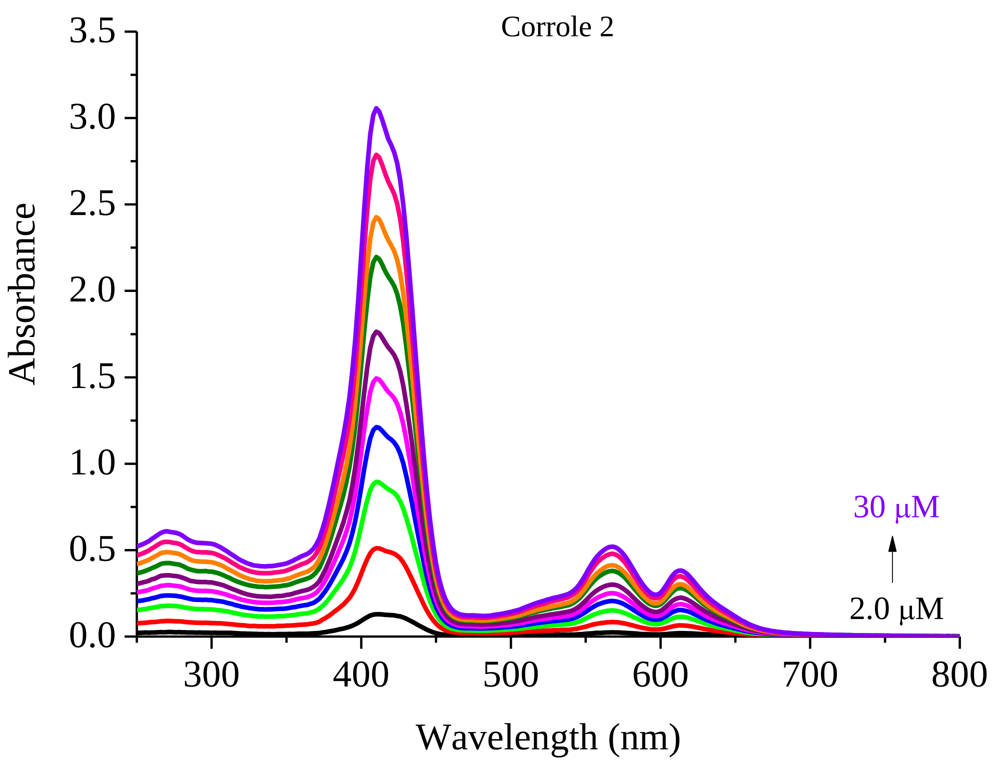

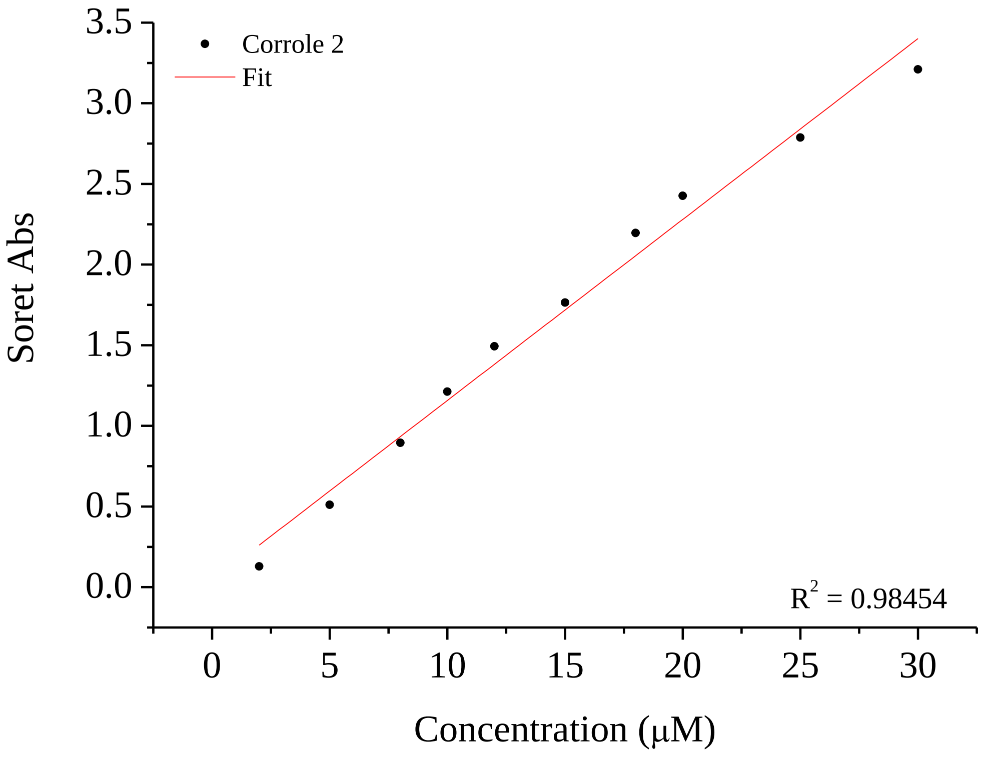

Figure S28. (up) Aggregation behavior of corrole **2** in MeOH solution and (down) Abs_{Soret} *versus* concentration plot.

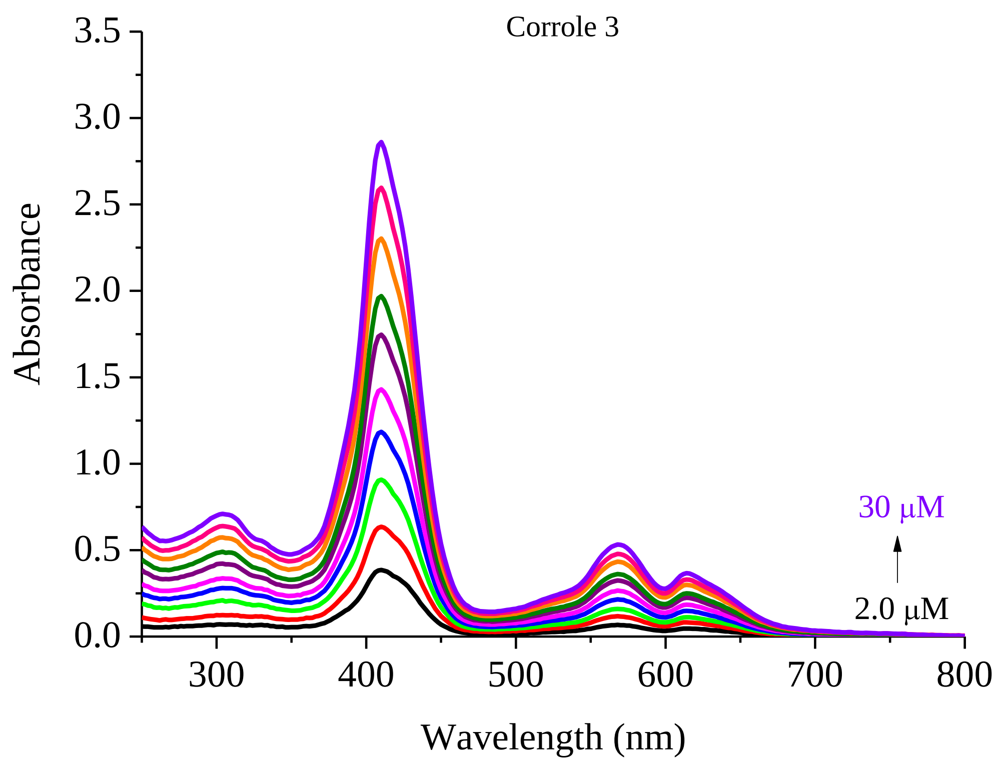

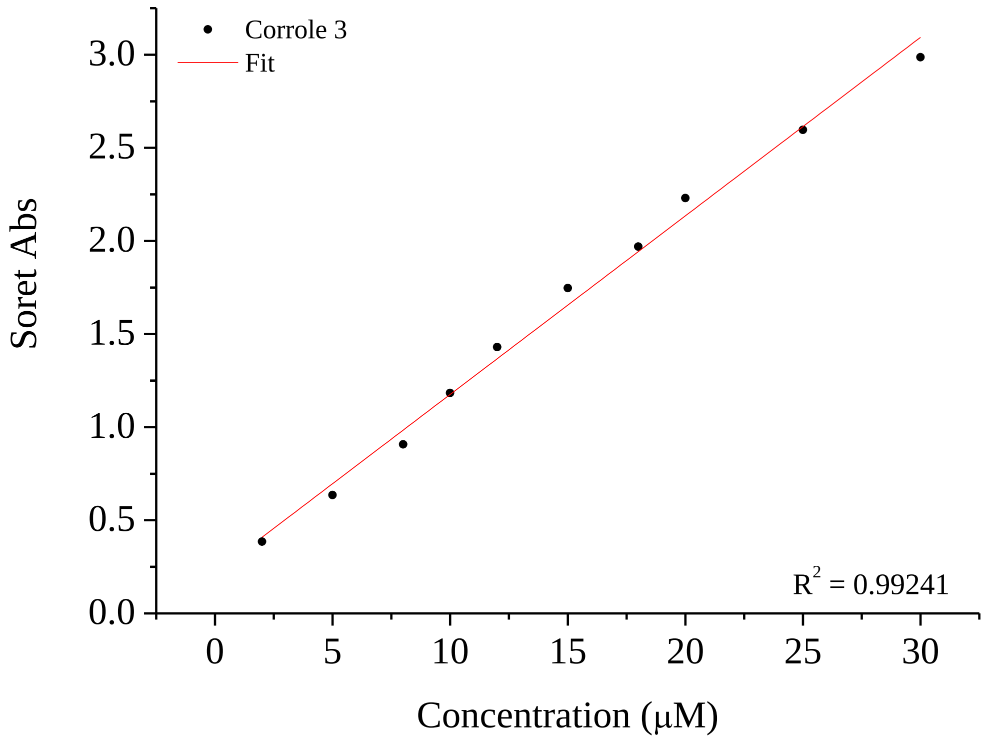

Figure S29. (up) Aggregation behavior of corrole **3** in MeOH solution and (down) Abs_{Soret} *versus* concentration plot.

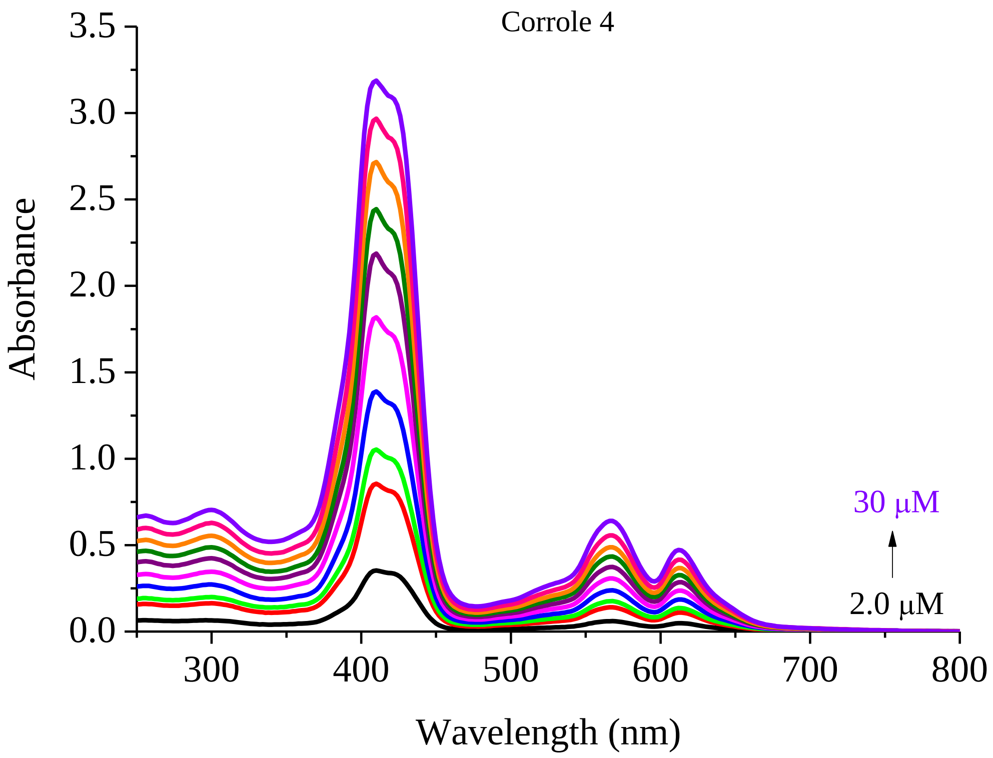

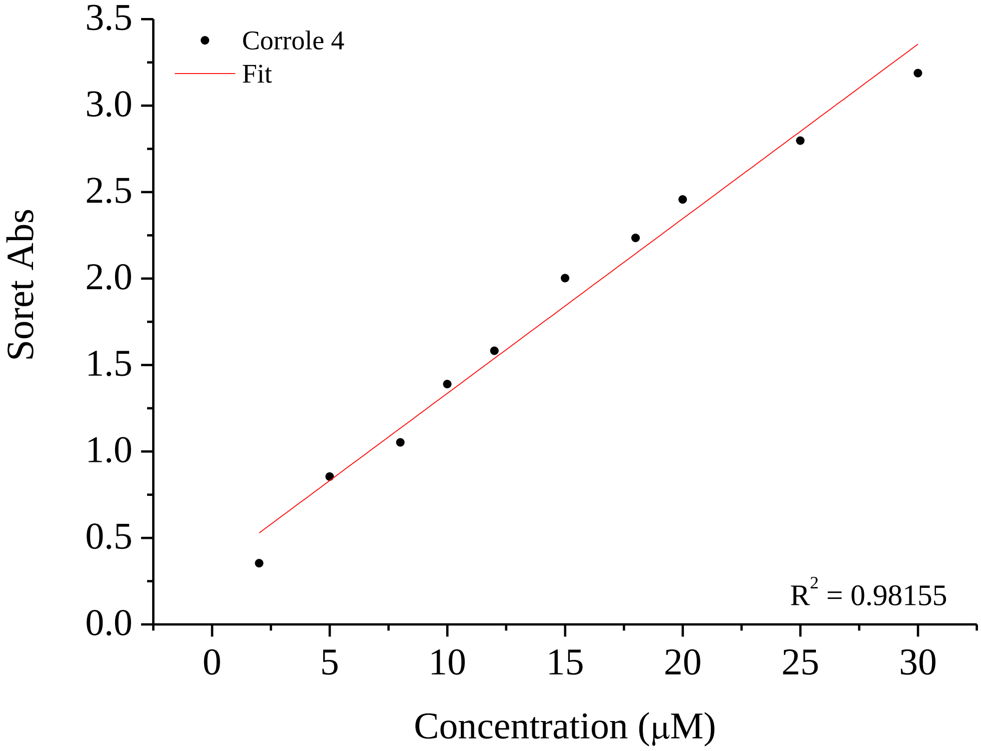

Figure S30. (up) Aggregation behavior of corrole **4** in MeOH solution and (down) Abs_{Soret} *versus* concentration plot.

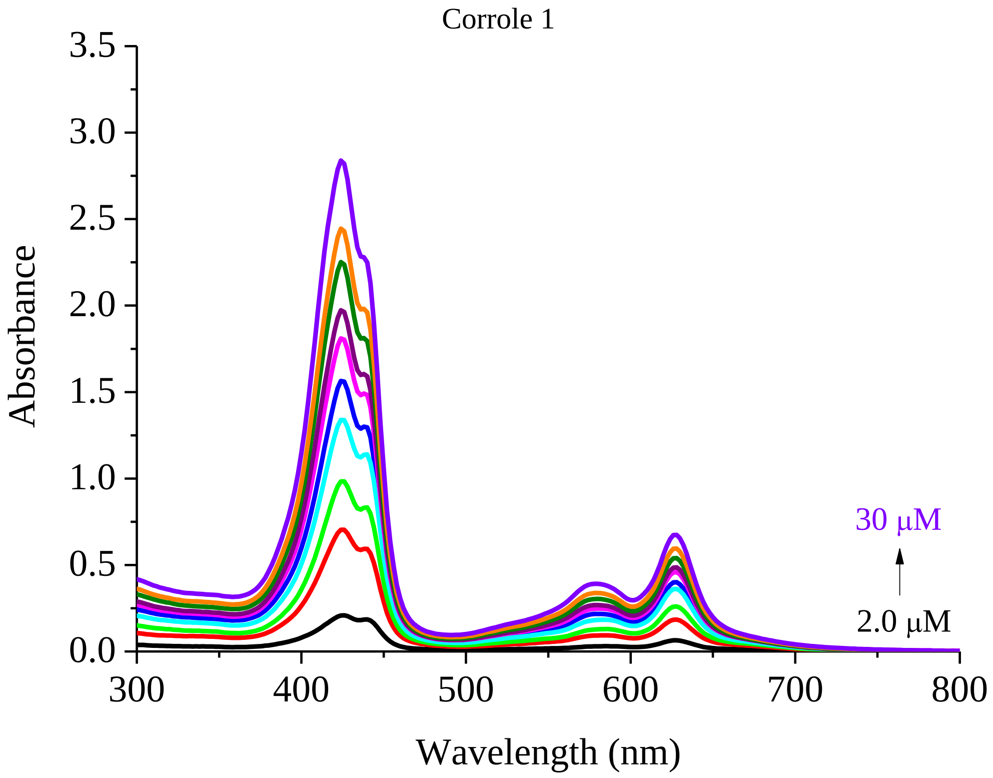

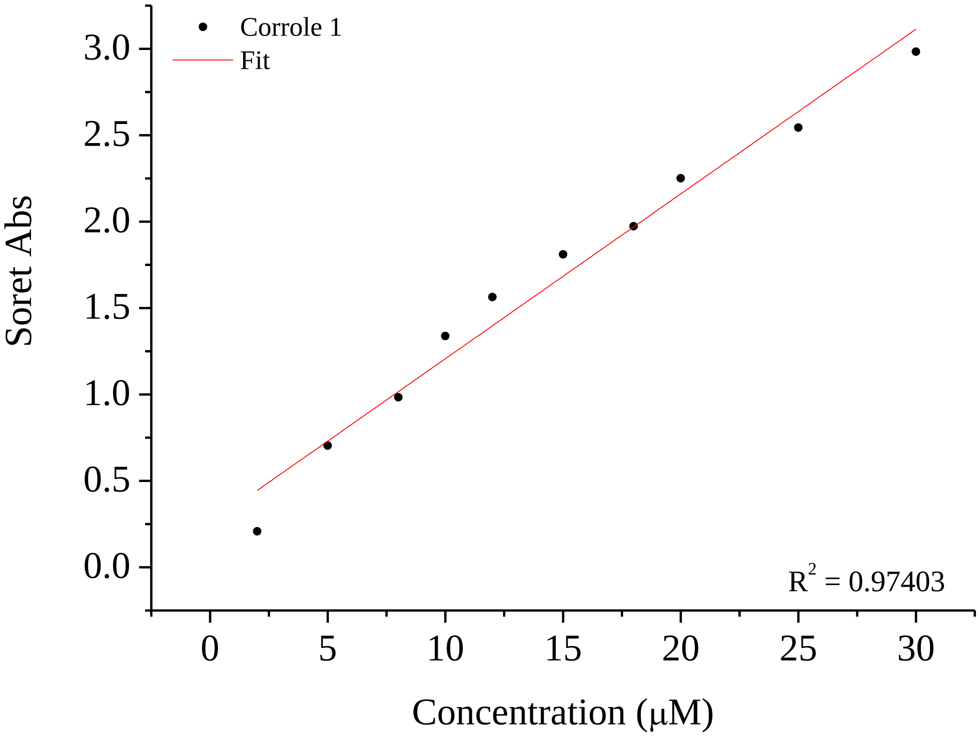

Figure S31. (up) Aggregation behavior of corrole **1** in DMSO solution and (down) Abs_{Soret} *versus* concentration plot.

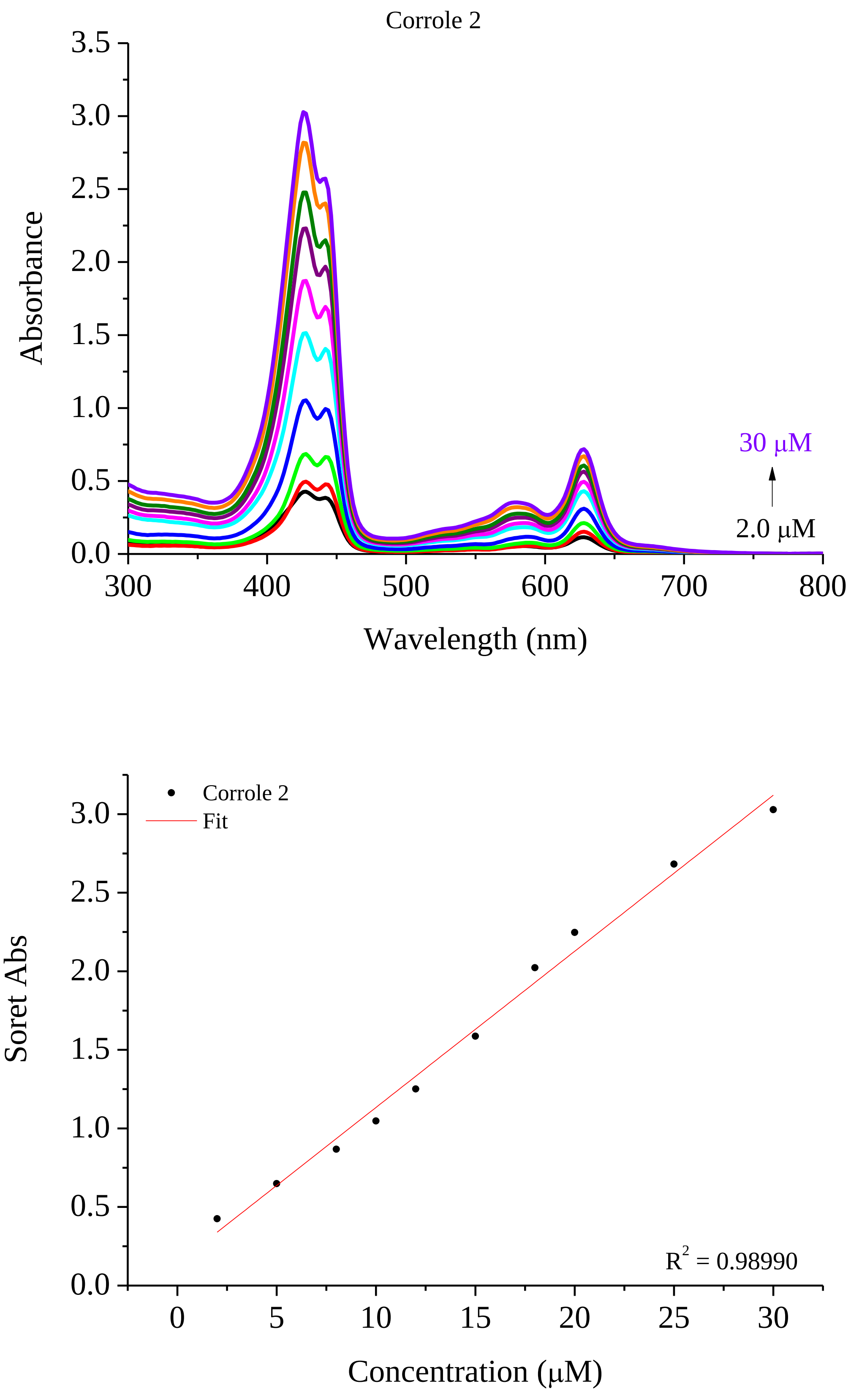

Figure S32. (up) Aggregation behavior of corrole **2** in DMSO solution and (down) Abs_{Soret} *versus* concentration plot.

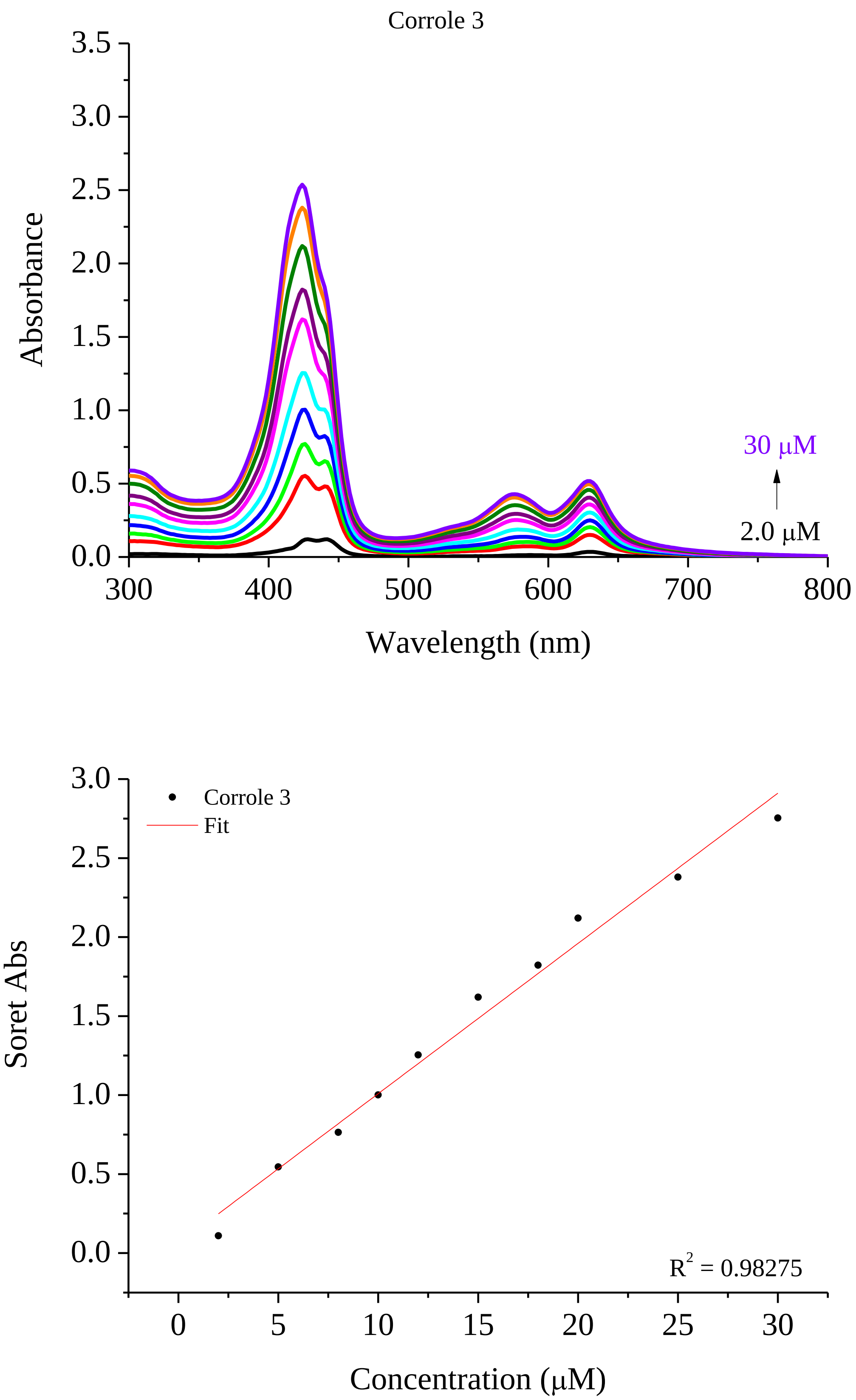

Figure S33. (up) Aggregation behavior of corrole **3** in DMSO solution and (down) Abs_{Soret} *versus* concentration plot.

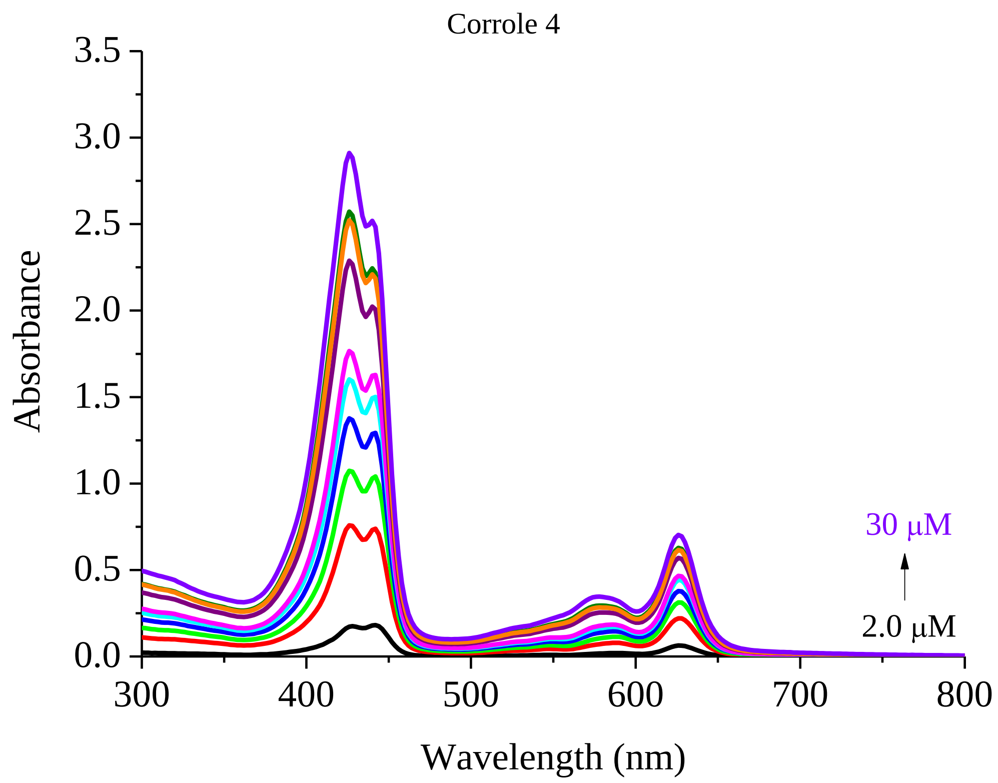

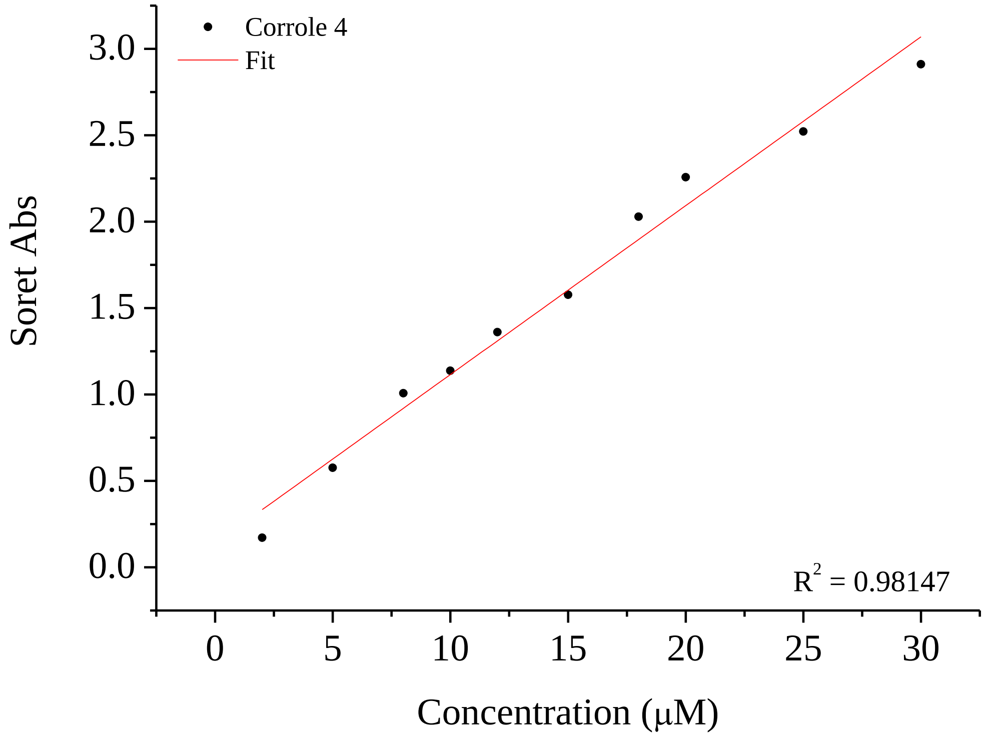

Figure S34. (up) Aggregation behavior of corrole **4** in DMSO solution and (down) Abs_{Soret} *versus* concentration plot.

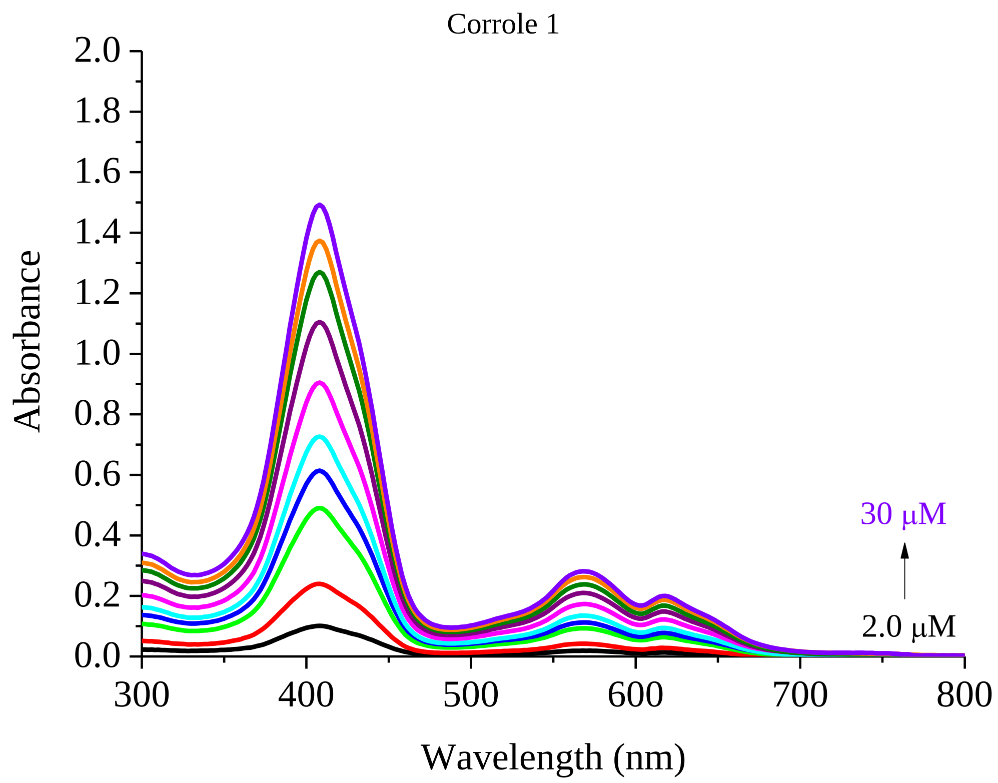

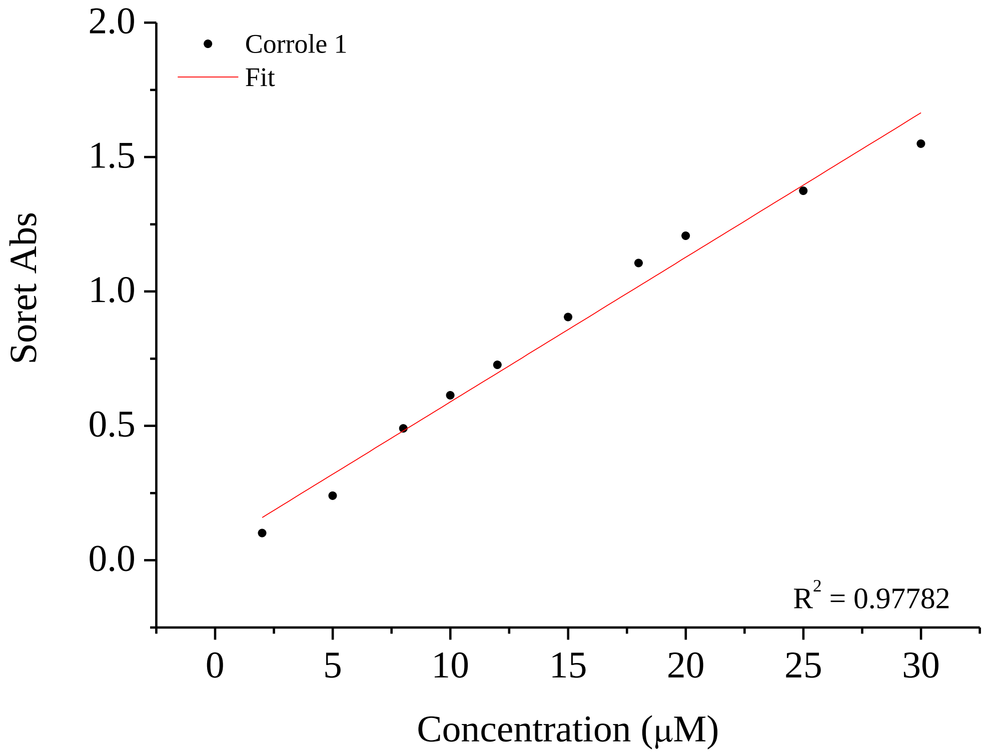

Figure S35. (up) Aggregation behavior of corrole **1** in DMSO(5%)/Tris-HCl pH 7.4 buffered mixture solution and (down) Abs_{Soret} *versus* concentration plot.

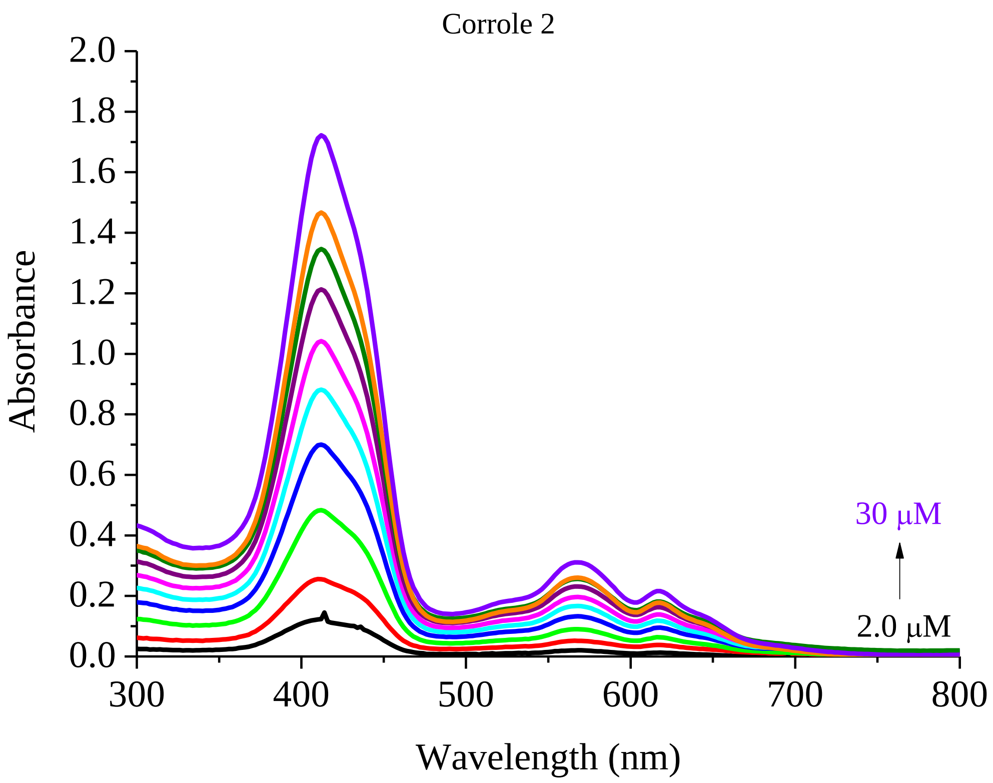

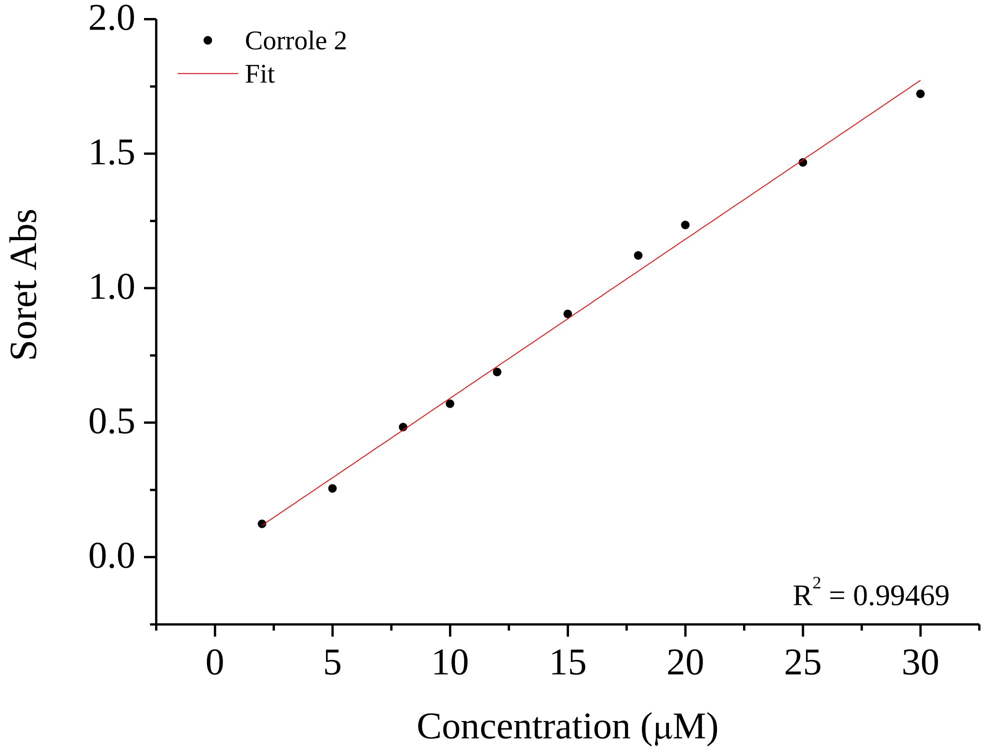

Figure S36. (up) Aggregation behavior of corrole **2** in DMSO(5%)/Tris-HCl pH 7.4 buffered mixture solution and (down) Abs_{Soret} *versus* concentration plot.

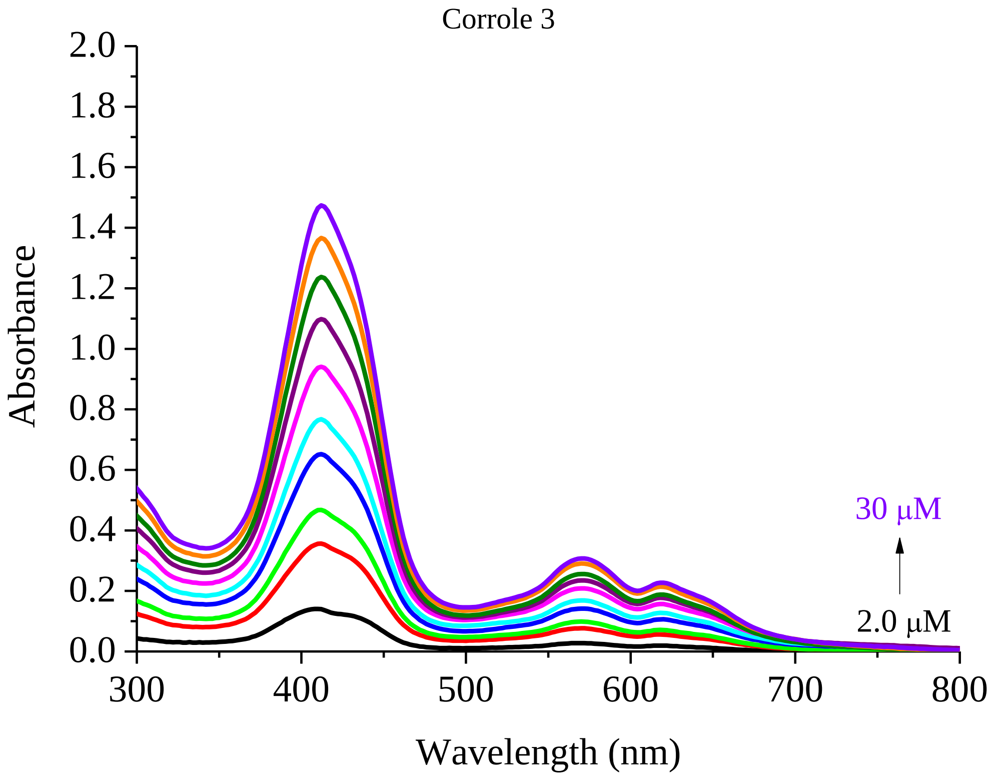

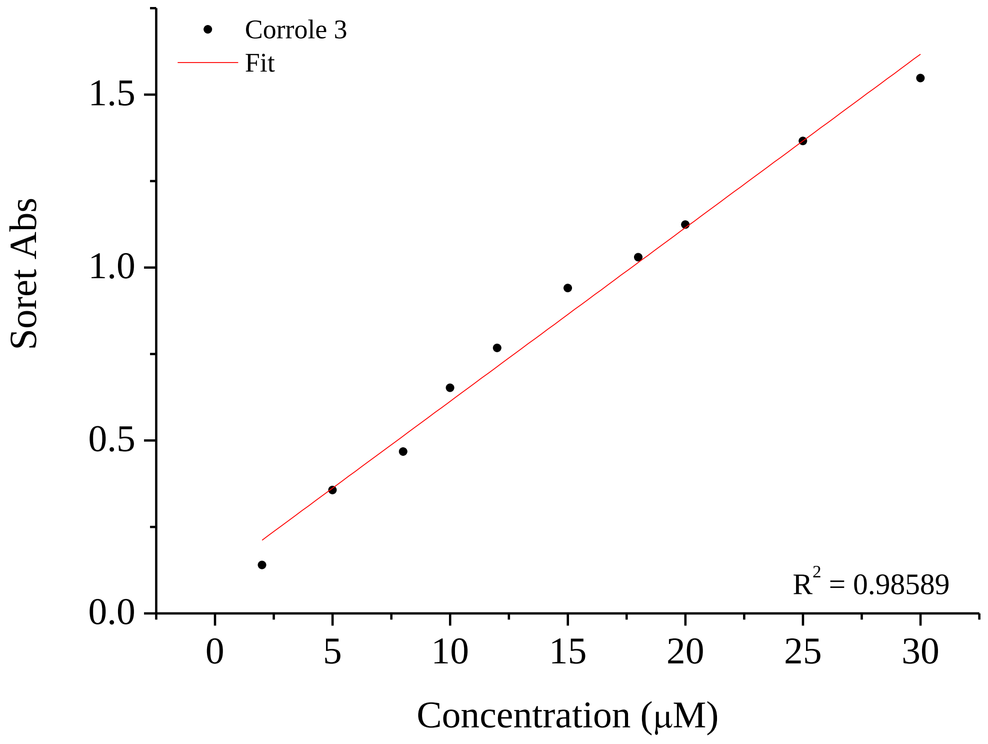

Figure S37. (up) Aggregation behavior of corrole **3** in DMSO(5%)/Tris-HCl pH 7.4 buffered mixture solution and (down) Abs_{Soret} *versus* concentration plot.

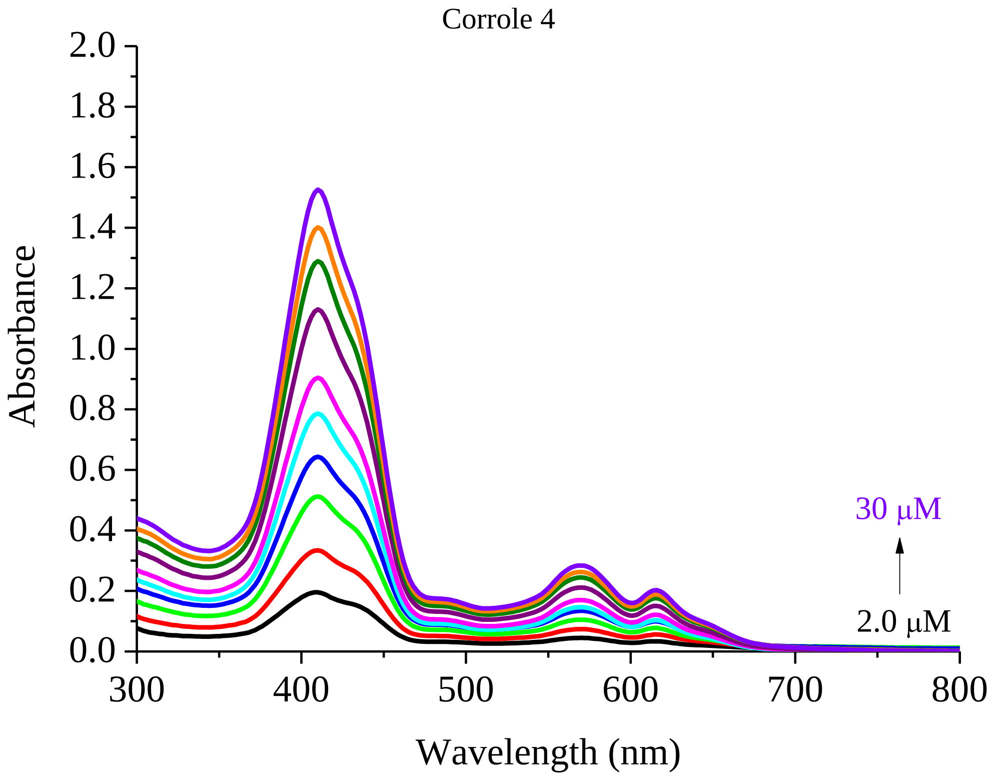

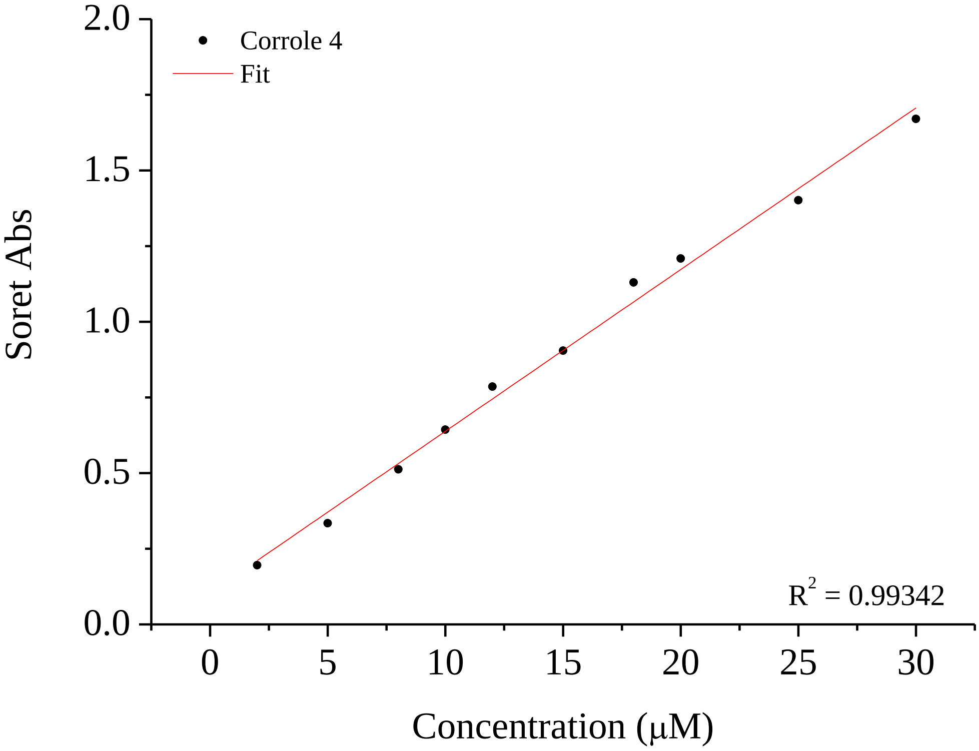

Figure S38. (up) Aggregation behavior of corrole **4** in DMSO(5%)/Tris-HCl pH 7.4 buffered mixture solution and (down) Abs_{Soret} *versus* concentration plot.

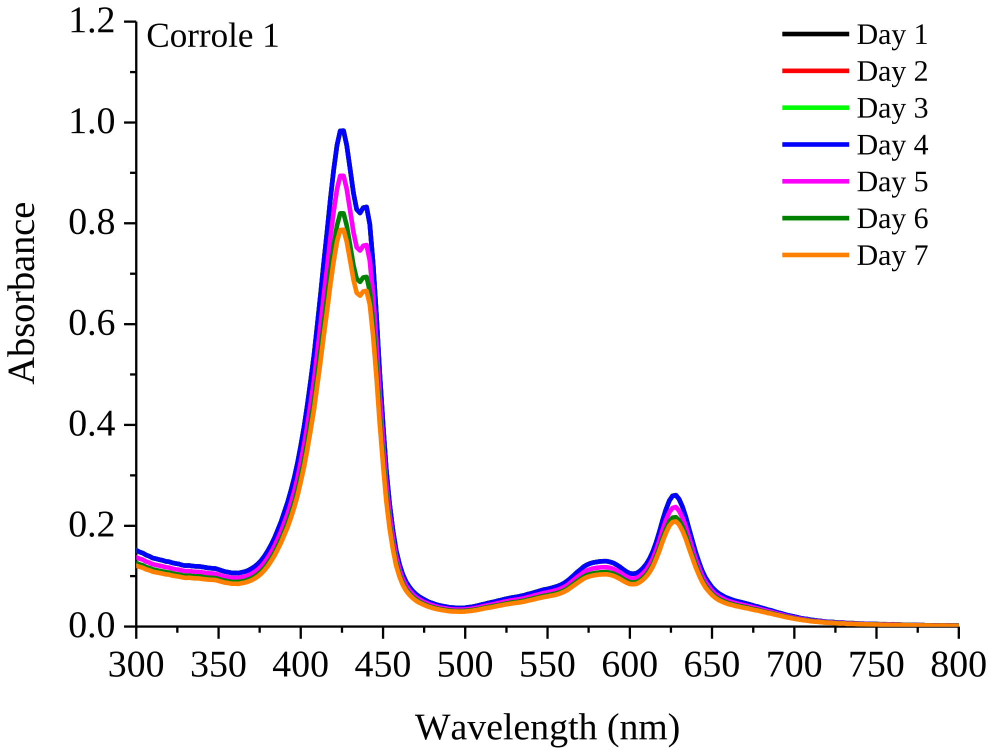

Figure S39. Stability assay of corrole **1** in DMSO solution.

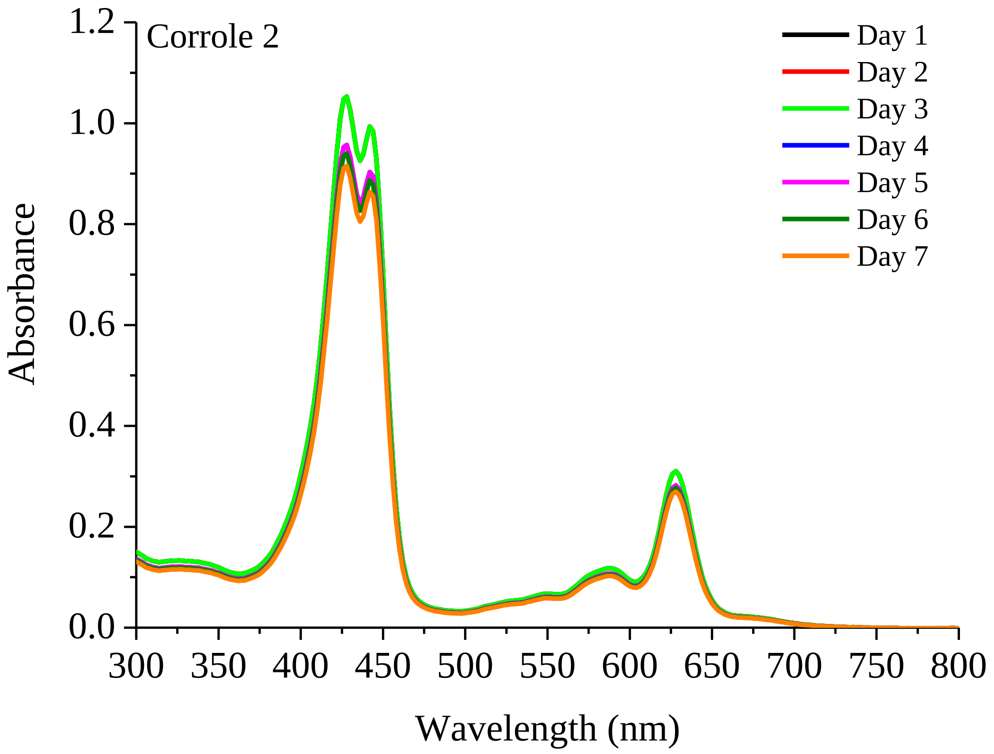

Figure S40. Stability assay of corrole **2** in DMSO solution.

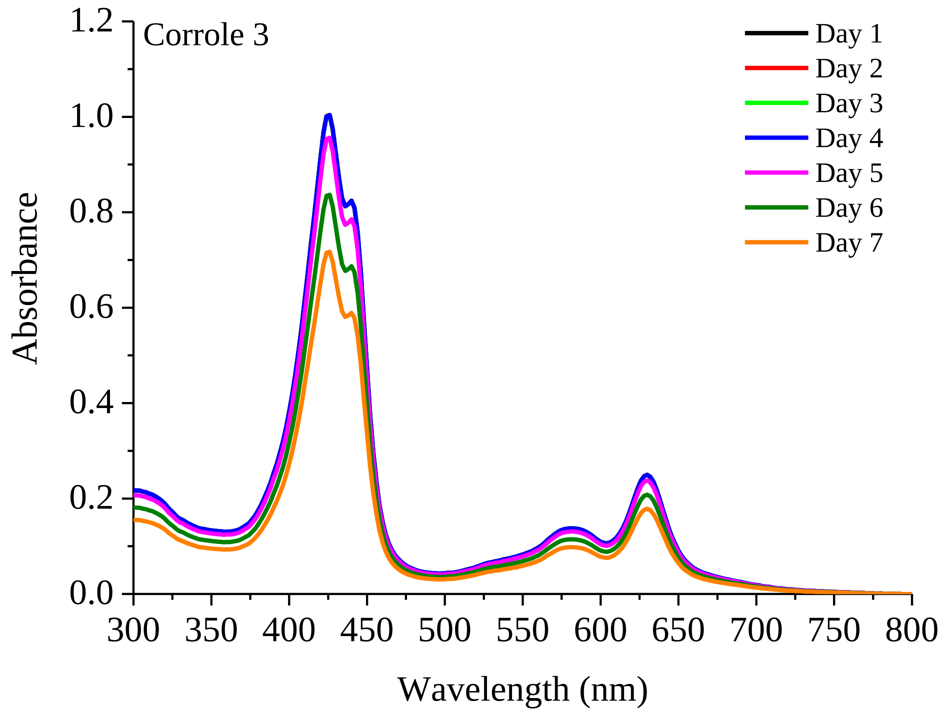

Figure S41. Stability assay of corrole **3** in DMSO solution.

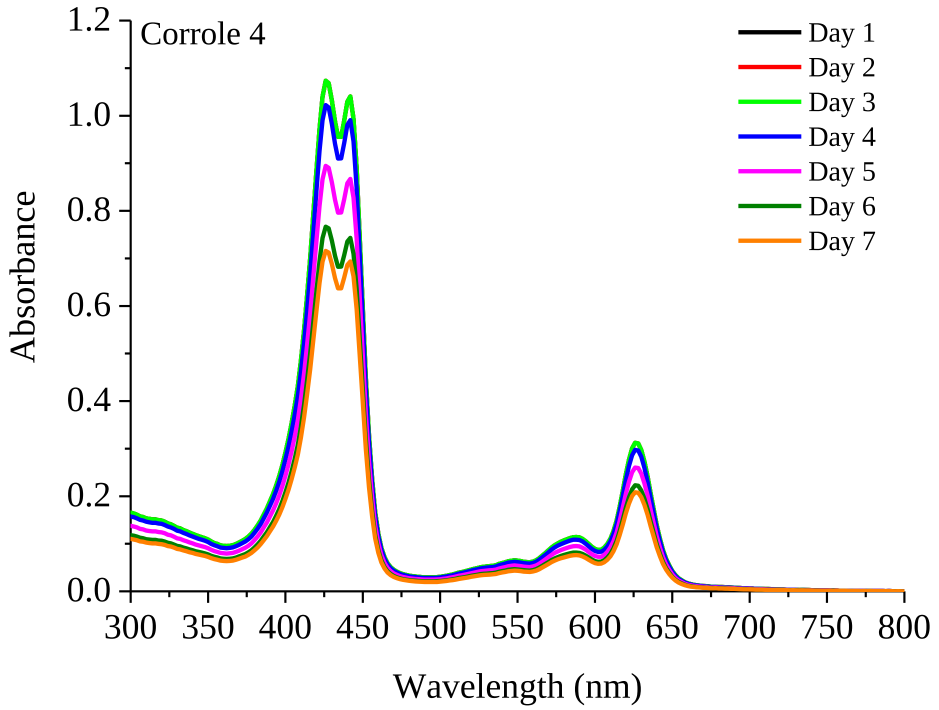

Figure S42. Stability assay of corrole **4** in DMSO solution.

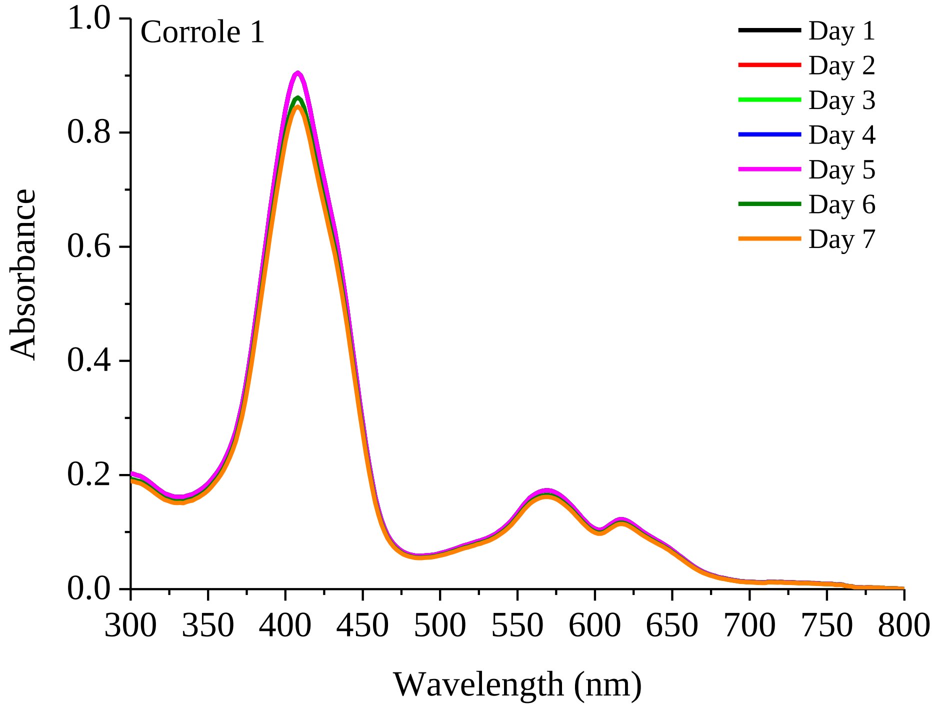

Figure S43. Stability assay of corrole **1** in DMSO(5%)/Tris-HCl pH 7.4 buffered mixture solution.

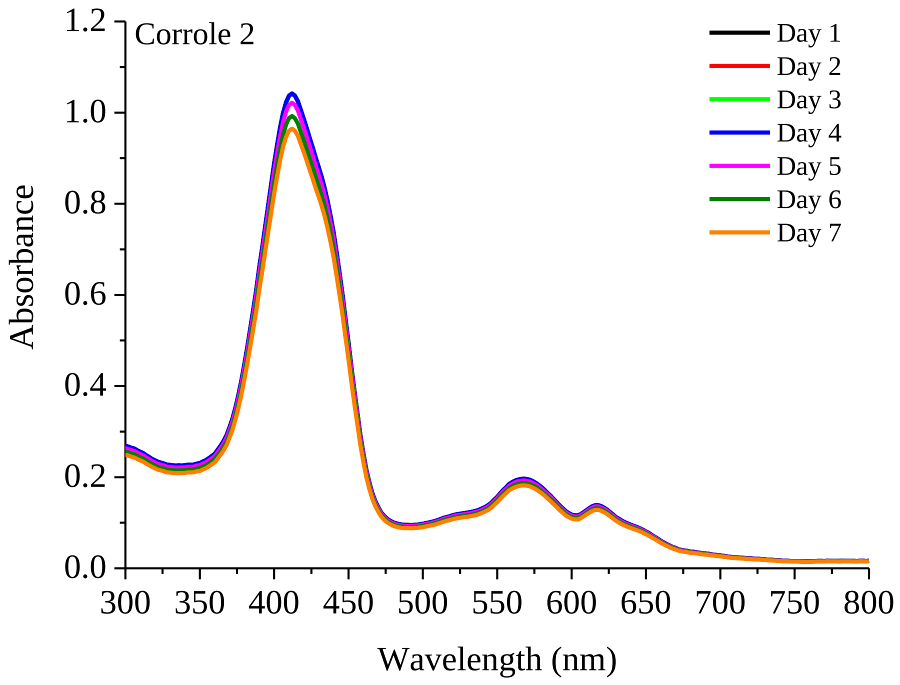

Figure S44. Stability assay of corrole **2** in DMSO(5%)/Tris-HCl pH 7.4 buffered mixture solution.

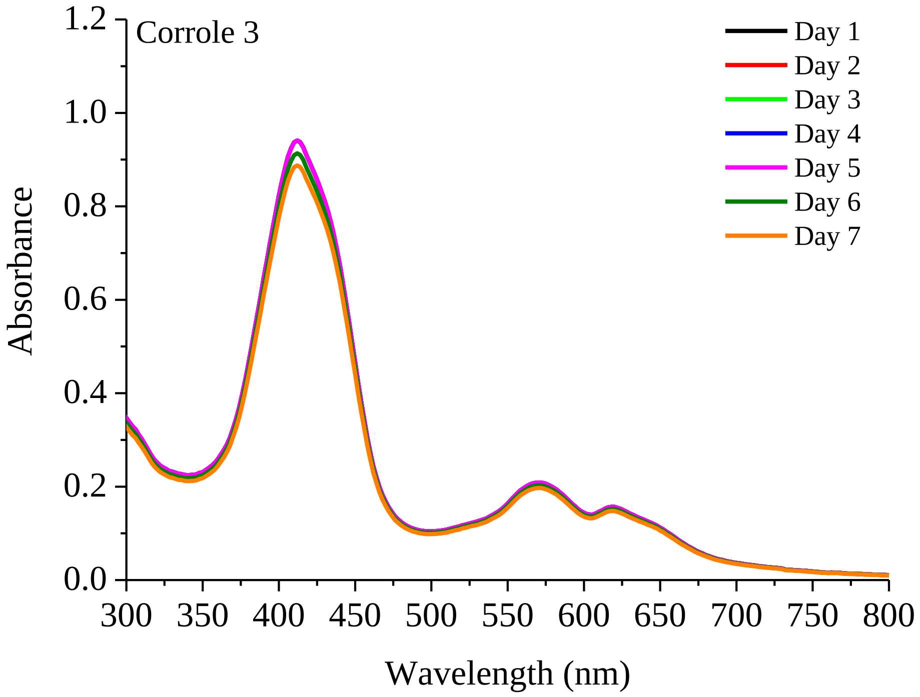

Figure S45. Stability assay of corrole **3** in DMSO(5%)/Tris-HCl pH 7.4 buffered mixture solution.

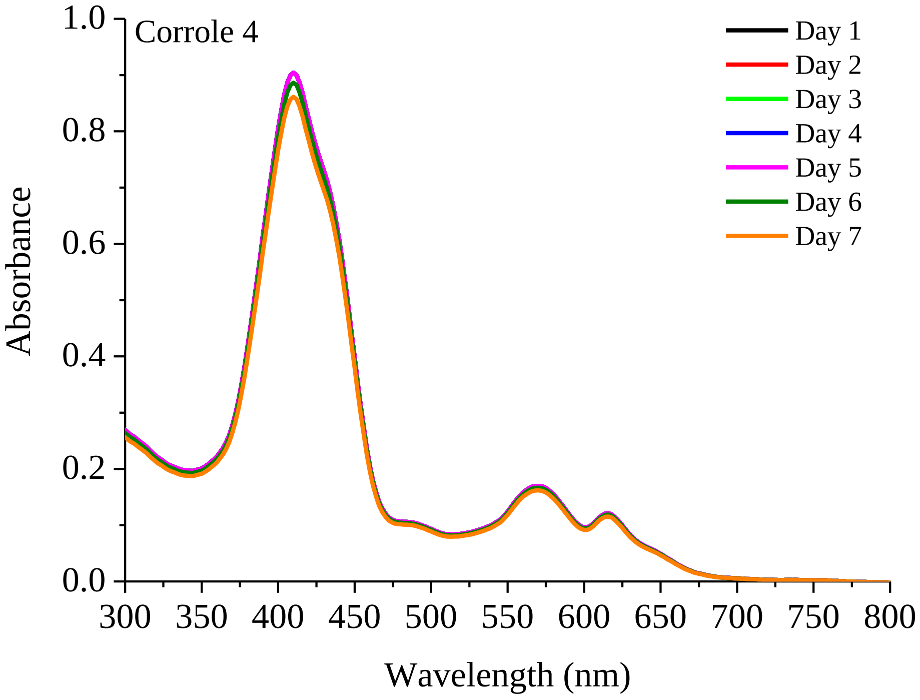

Figure S46. Stability assay of corrole **4** in DMSO(5%)/Tris-HCl pH 7.4 buffered mixture solution.

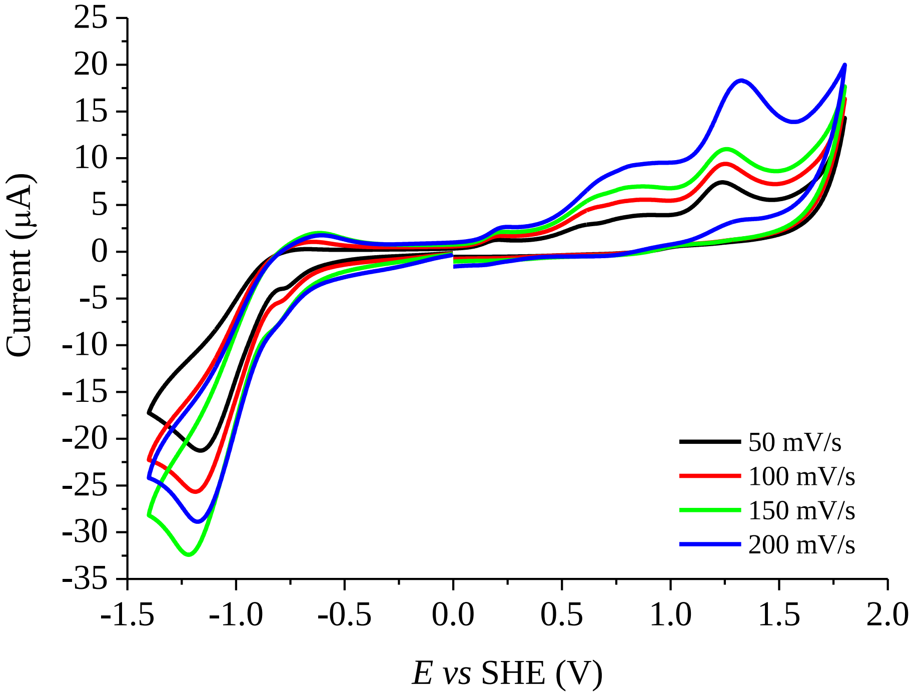

Figure S47. Cyclic voltammetry analysis of corrole **1** in dry DCM solution, using 0.1 M $TBAPF_6$ as support electrolyte.

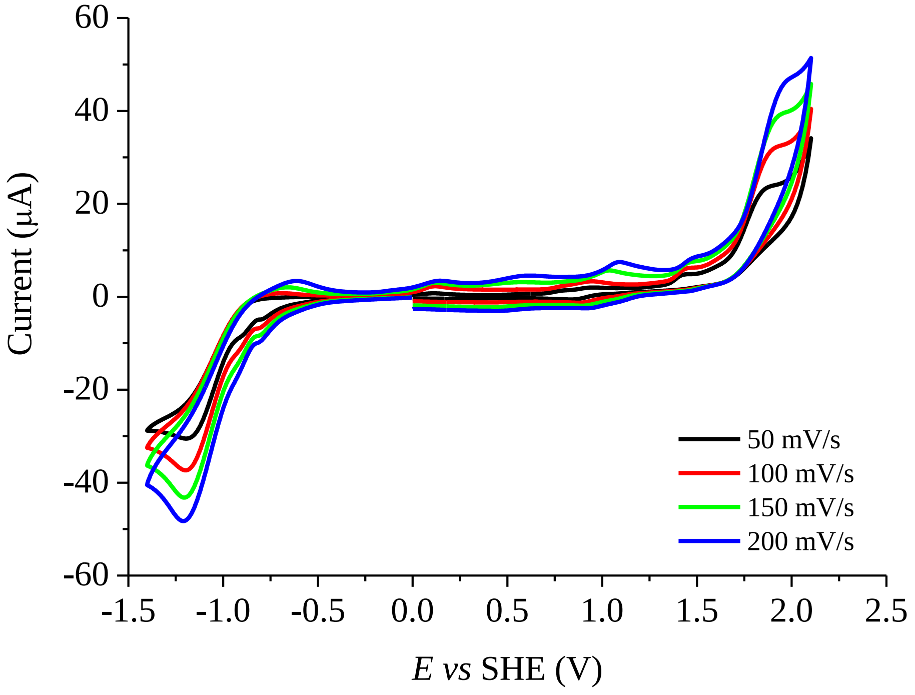

Figure S48. Cyclic voltammetry analysis of corrole **2** in dry DCM solution, using 0.1 M $TBAPF_6$ as support electrolyte.

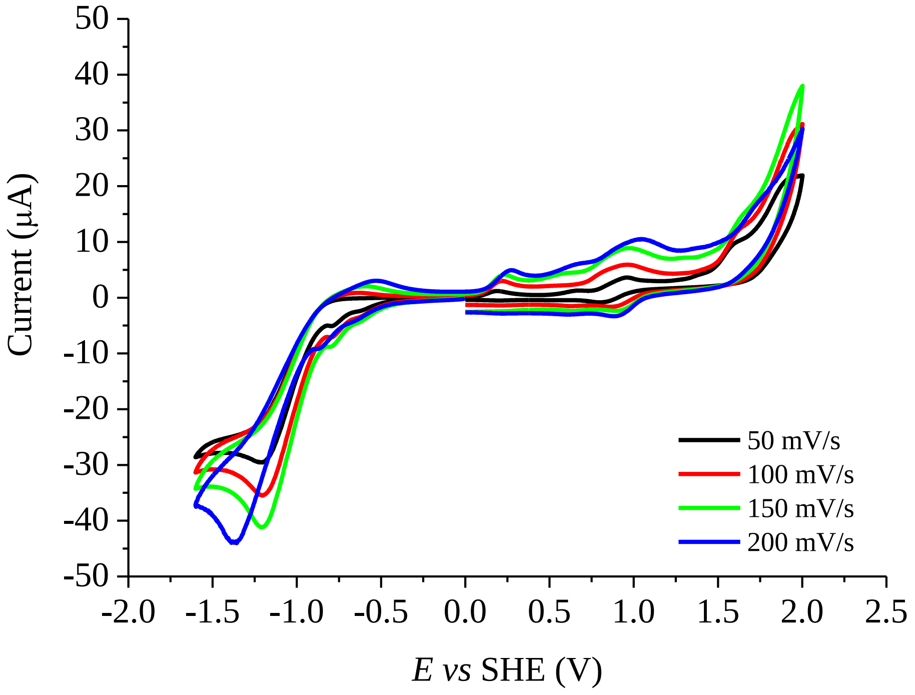

Figure S49. Cyclic voltammetry analysis of corrole **3** in dry DCM solution, using 0.1 M $TBAPF_6$ as support electrolyte.

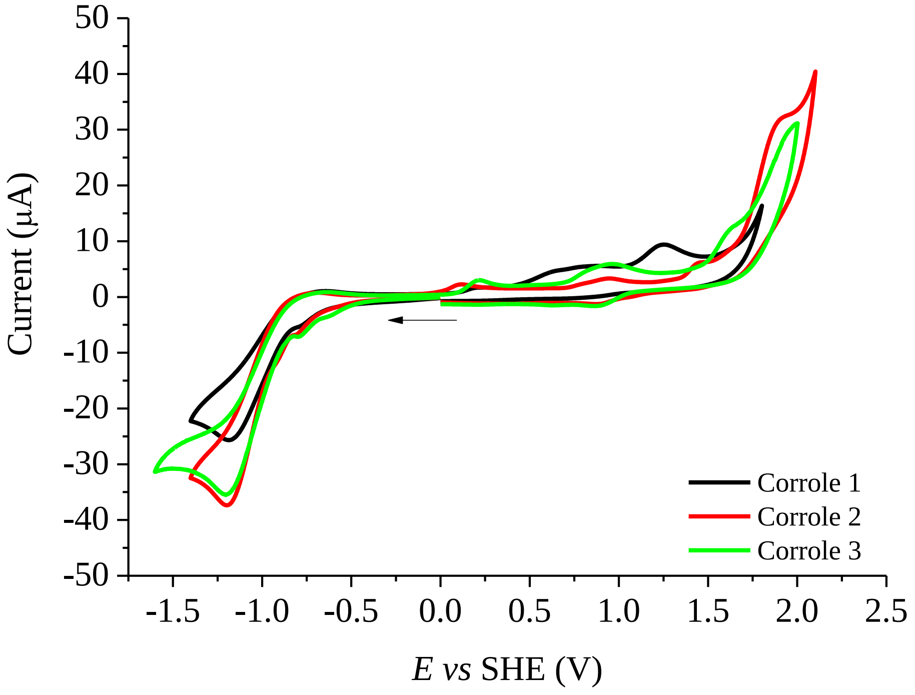

Figure S50. Cyclic voltammetry analysis of corrole **4** in dry DCM solution, using 0.1 M $TBAPF_6$ as support electrolyte.

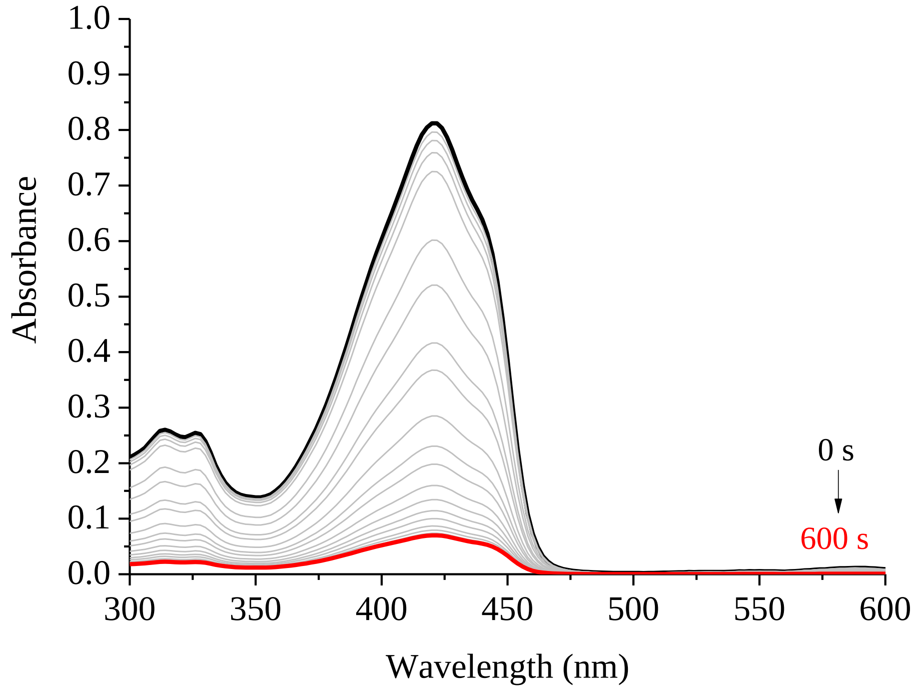

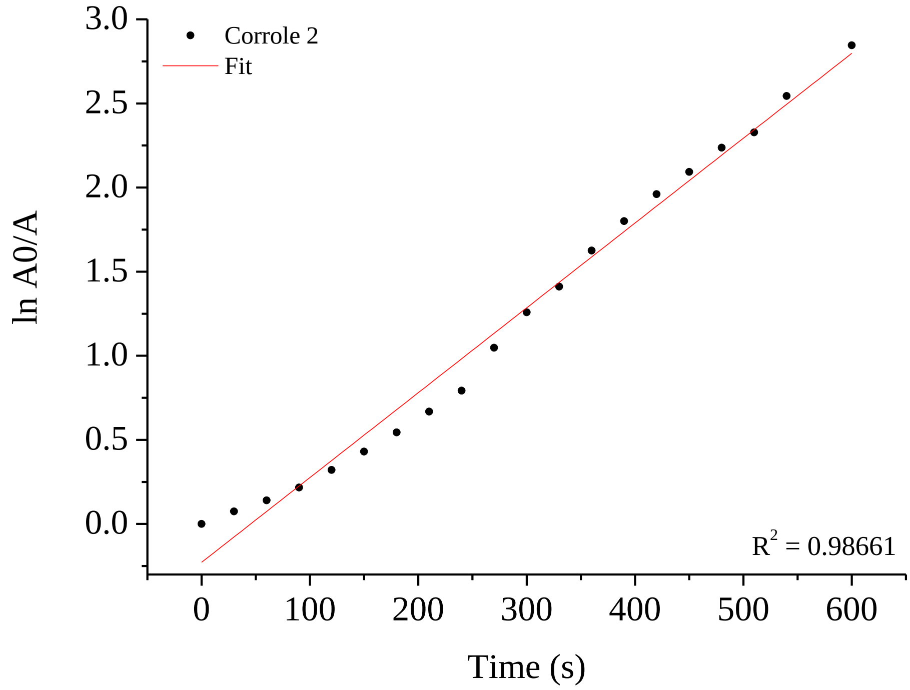

Figure S51. (up) DPBF photo-oxidation assay of corrole **2** in DMSO solution, by red-light irradiation source at 600 s and (down) ln A_0/A *versus* time plot.

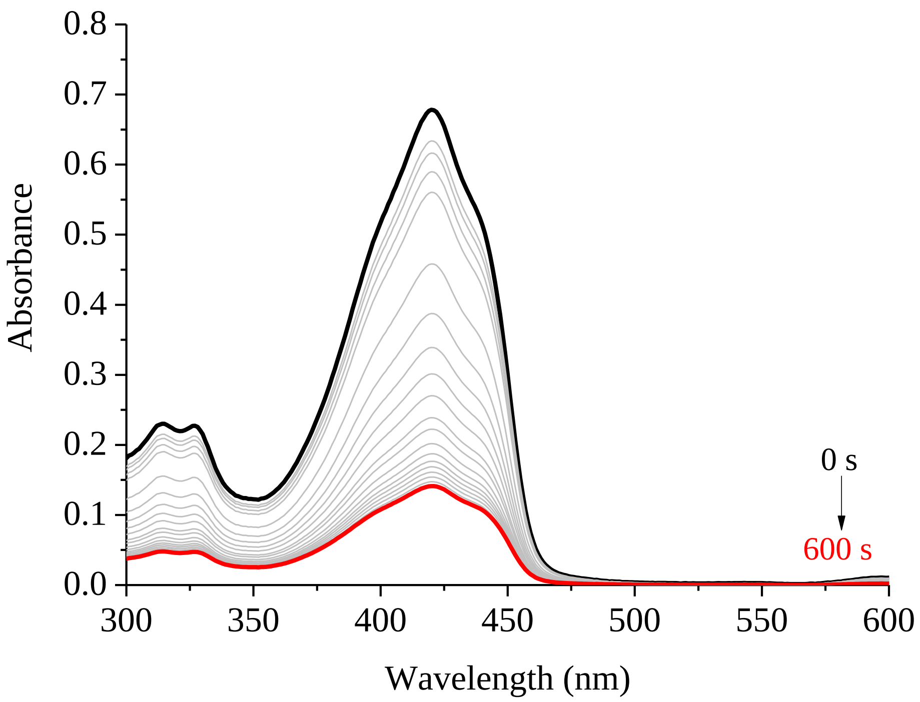

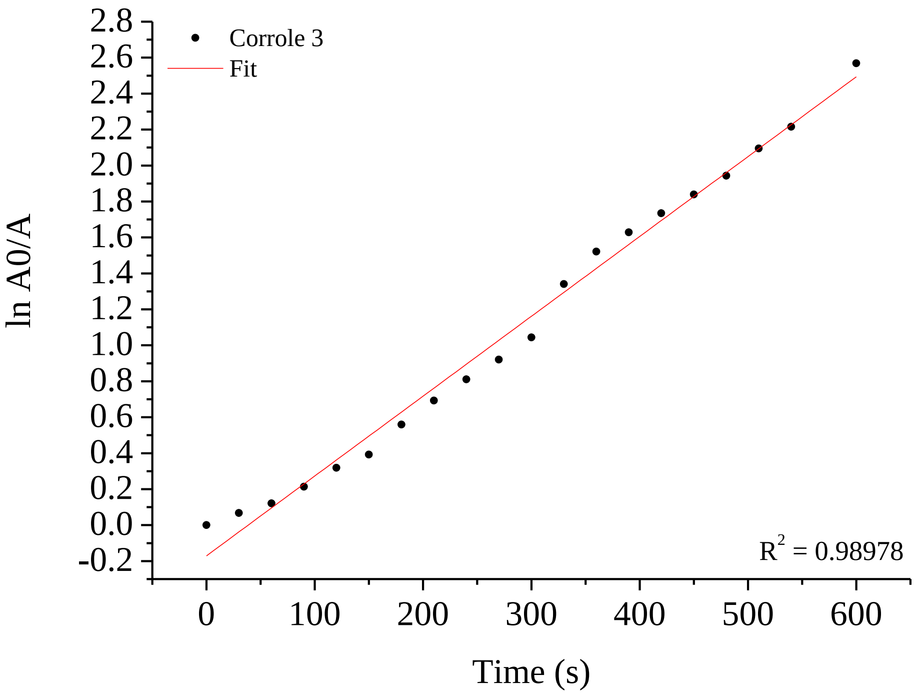

Figure S52. (up) DPBF photo-oxidation assay of corrole **3** in DMSO solution, by red-light irradiation source at 600 s and (down) ln A_0/A *versus* time plot.

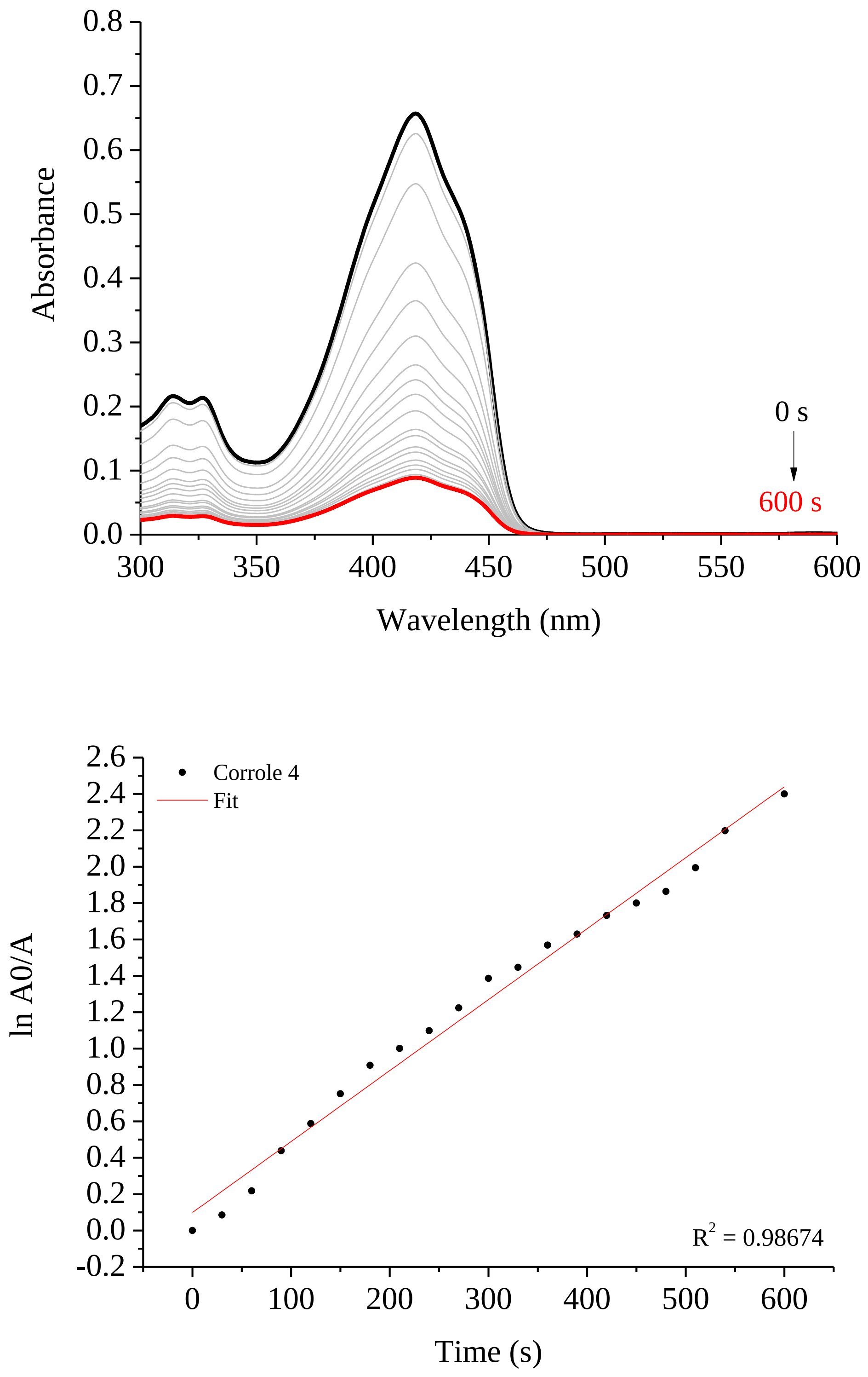

Figure S53. (up) DPBF photo-oxidation assay of corrole **4** in DMSO solution, by red-light irradiation source at 600 s and (down) ln A_0/A *versus* time plot.

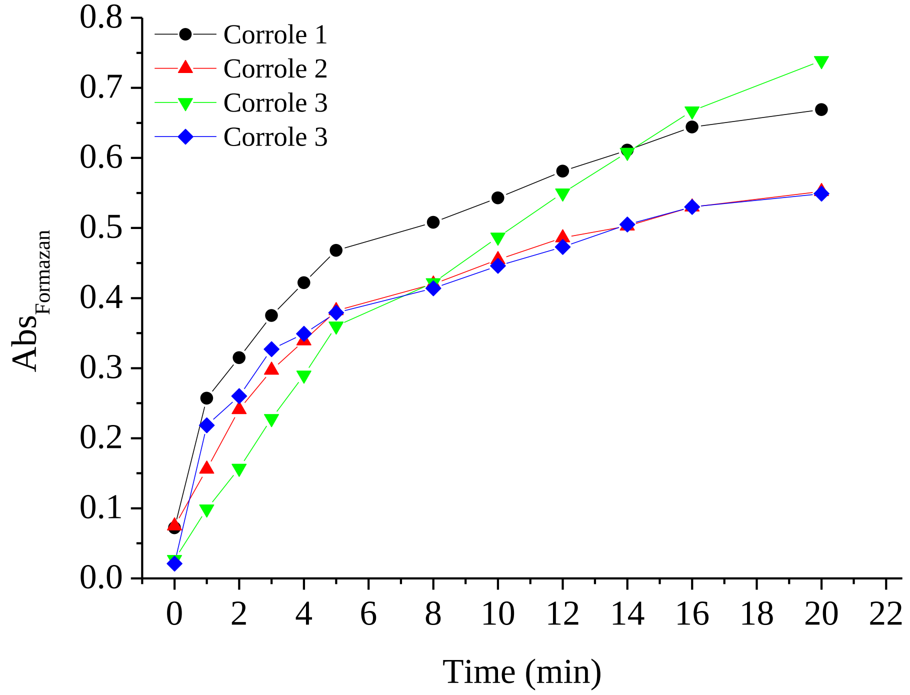

Figure S54. NBT reduction assay of corroles **1-4** in DMSO solution, by white-light irradiation source at 20 min.

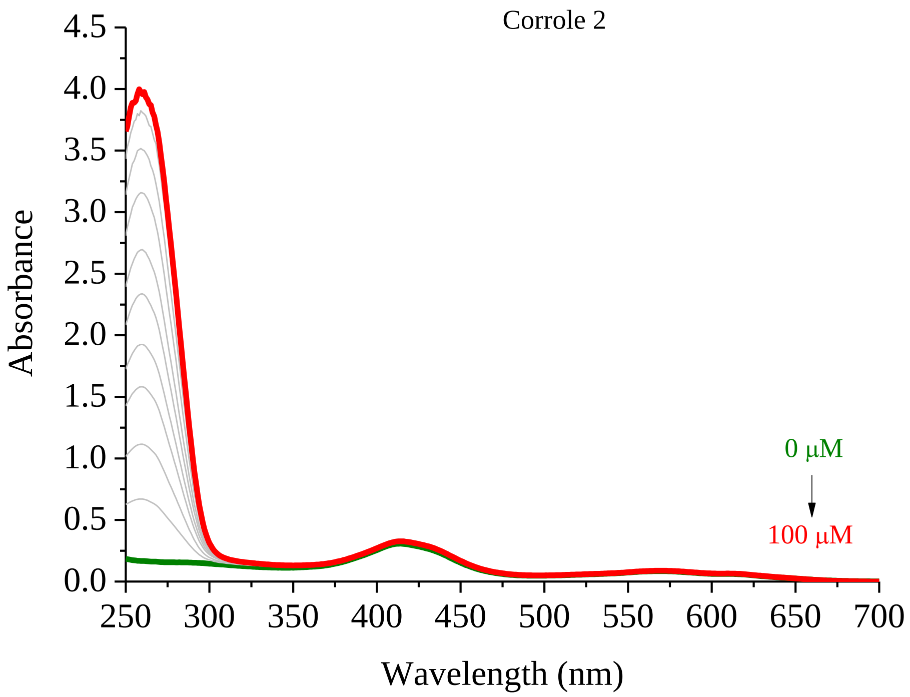

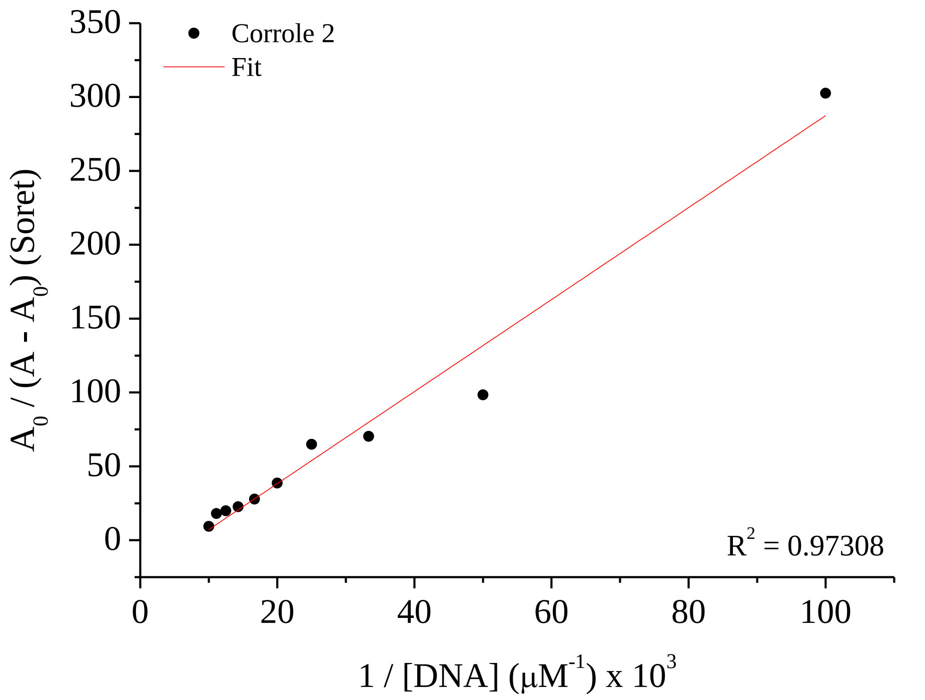

Figure S55. **(up)** UV-Vis spectra of the corrole **2** upon successive additions of CT-DNA concentrations (0 to 100 μM) in DMSO(5%)/Tris-HCl pH 7.4 mixture buffered solution. **(down)** Benesi-Hidelbrandt plots of A_0 / (A – A_0) *versus* 1 / [CT-DNA].

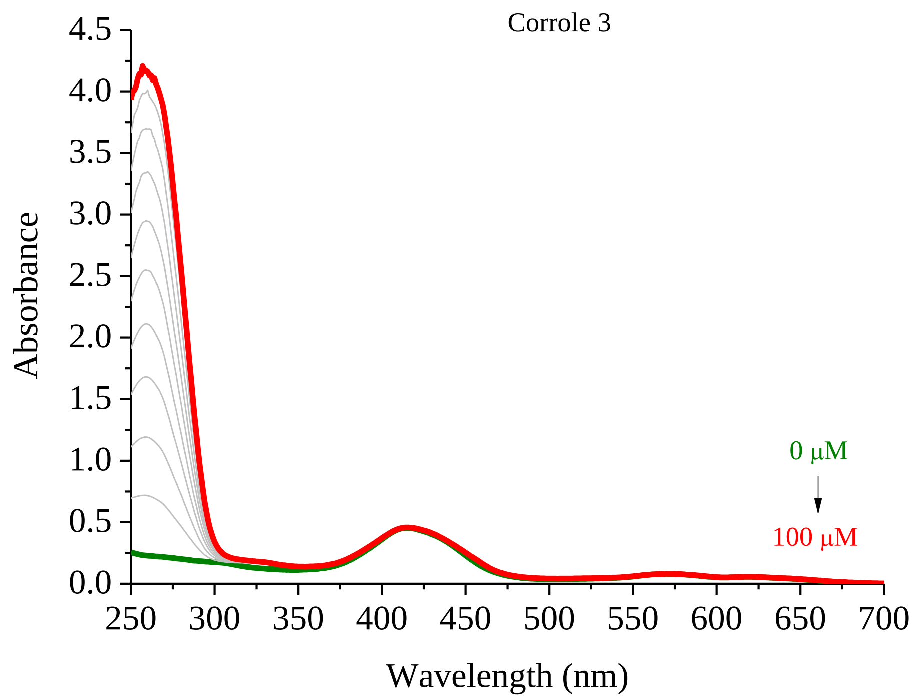

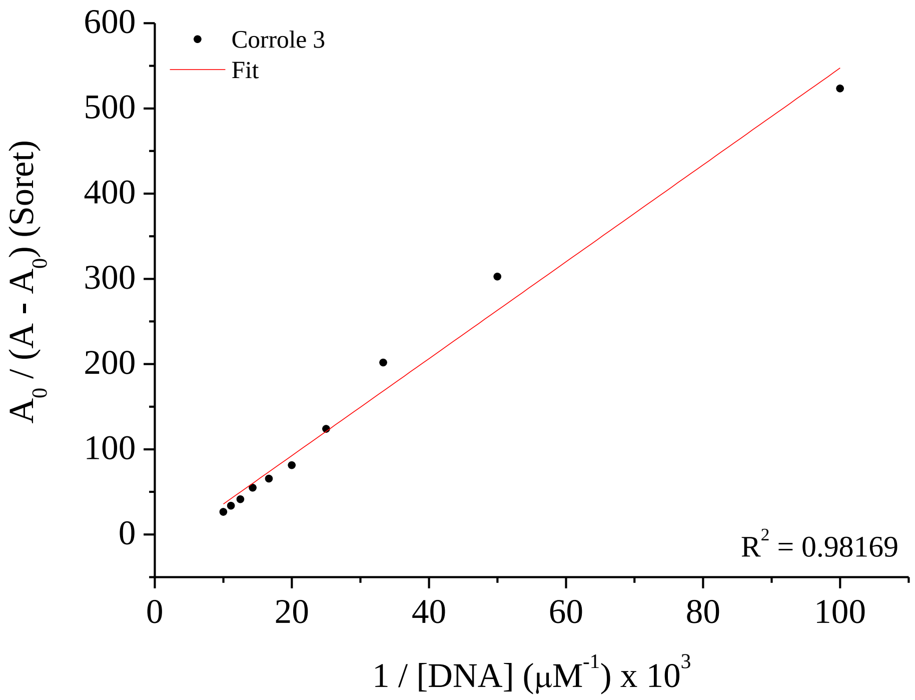

Figure S56. **(up)** UV-Vis spectra of the corrole **3** upon successive additions of CT-DNA concentrations (0 to 100 µM) in DMSO(5%)/Tris-HCl pH 7.4 mixture buffered solution. **(down)** Benesi-Hidelbrandt plots of A_0 / (A – A_0) *versus* 1 / [CT-DNA].

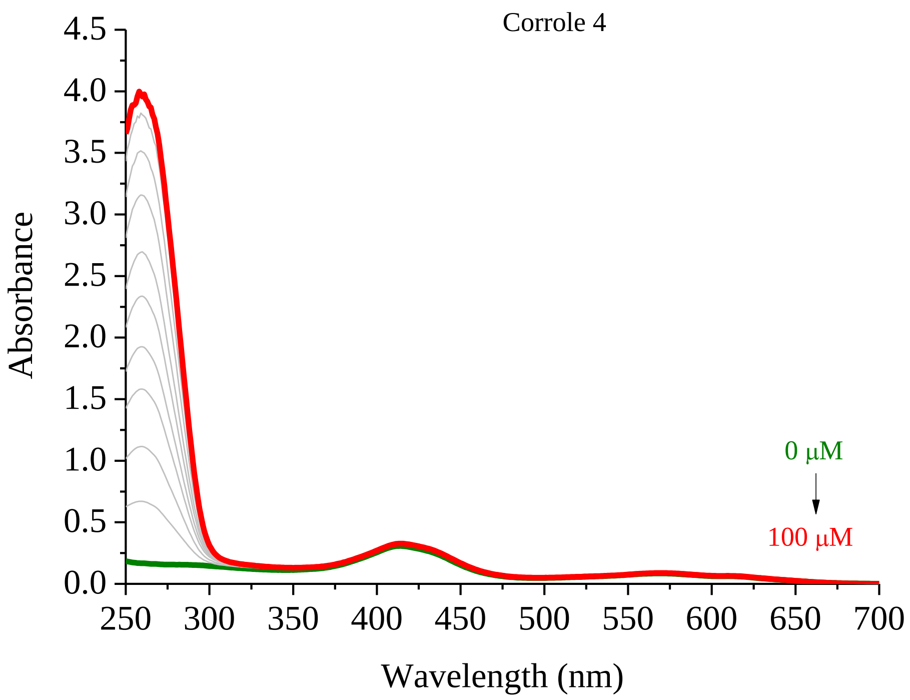

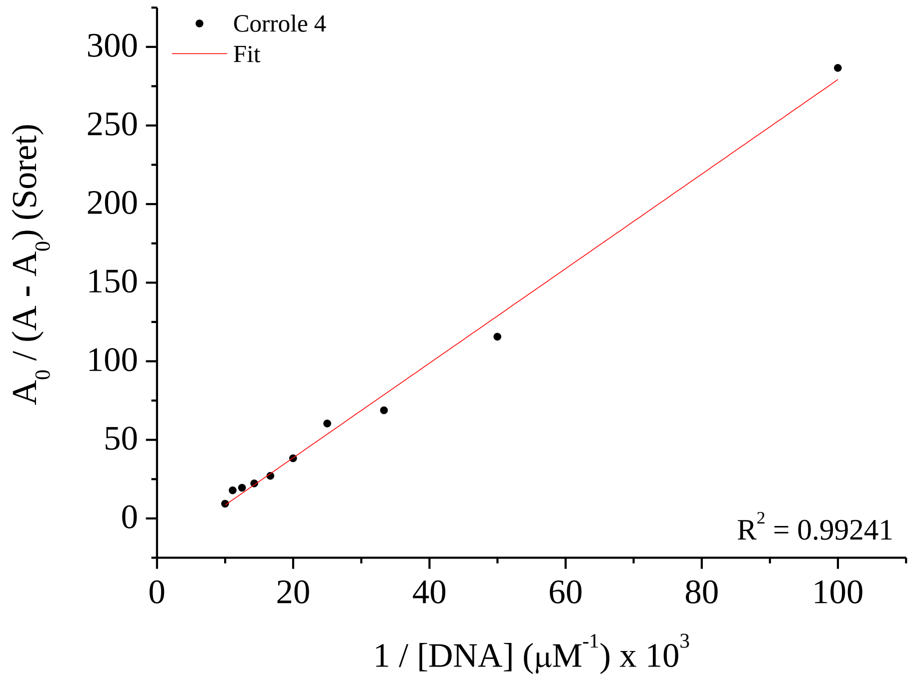

Figure S57. **(up)** UV-Vis spectra of the corrole **4** upon successive additions of CT-DNA concentrations (0 to 100 µM) in DMSO(5%)/Tris-HCl pH 7.4 mixture buffered solution. **(down)** Benesi-Hidelbrandt plots of A_0 / (A – A_0) *versus* 1 / [CT-DNA].

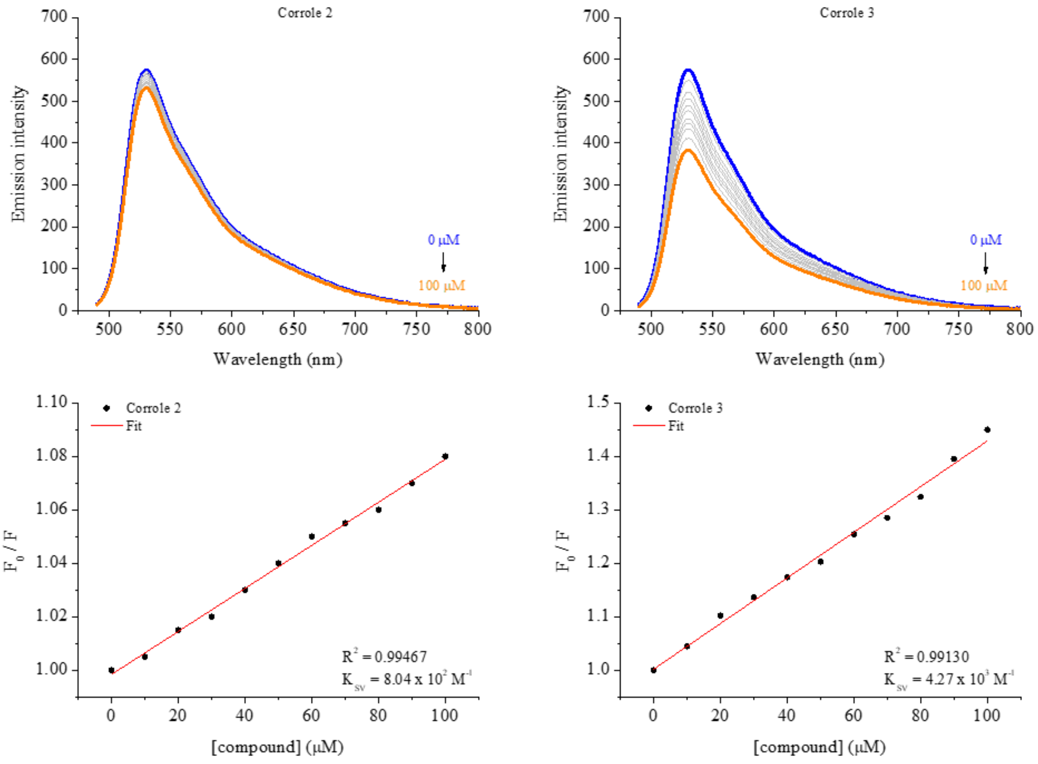

Figure S58. Steady-state fluorescence emission spectra for AO:DNA without and in the presence of corroles **2** and **3**, in DMSO(5%)/Tris-HCl pH 7.4 mixture buffered solution. Graphs shows the plot F_0/F *versus* [corrole]. [corrole] = 0–100 µM.

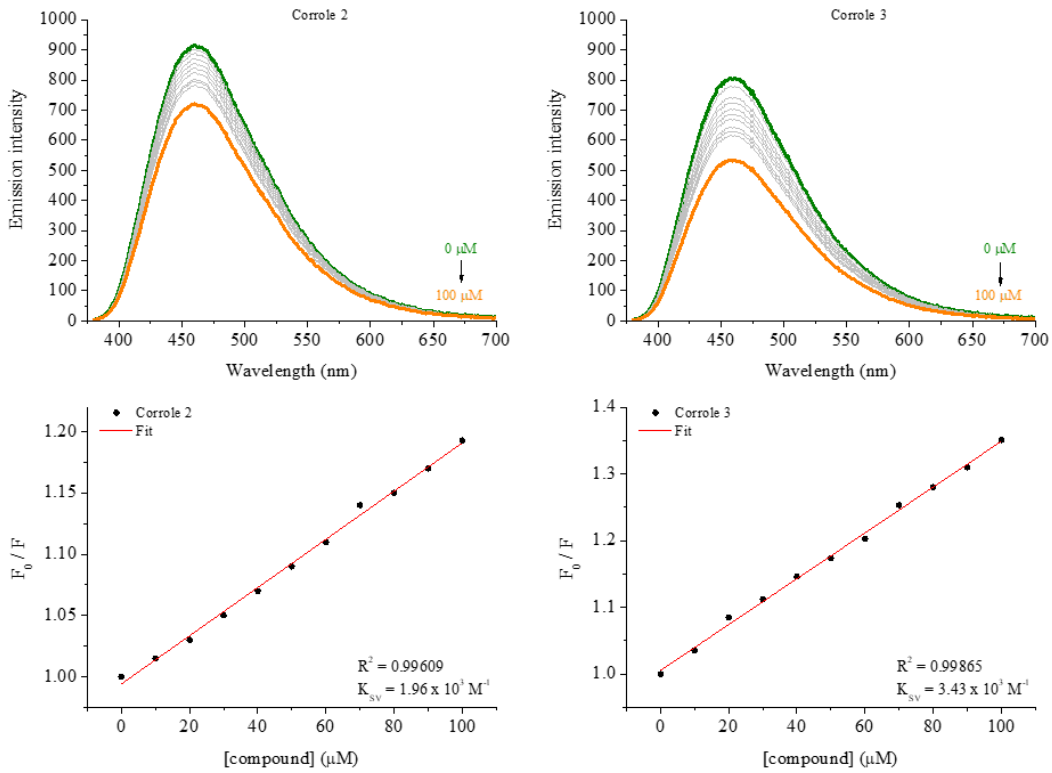

Figure S59. Steady-state fluorescence emission spectra for DAPI:DNA without and in the presence of corroles **2** and **3**, in DMSO(5%)/Tris-HCl pH 7.4 mixture buffered solution. Graphs shows the plot F_0/F *versus* [corrole]. [corrole] = 0–100 µM.

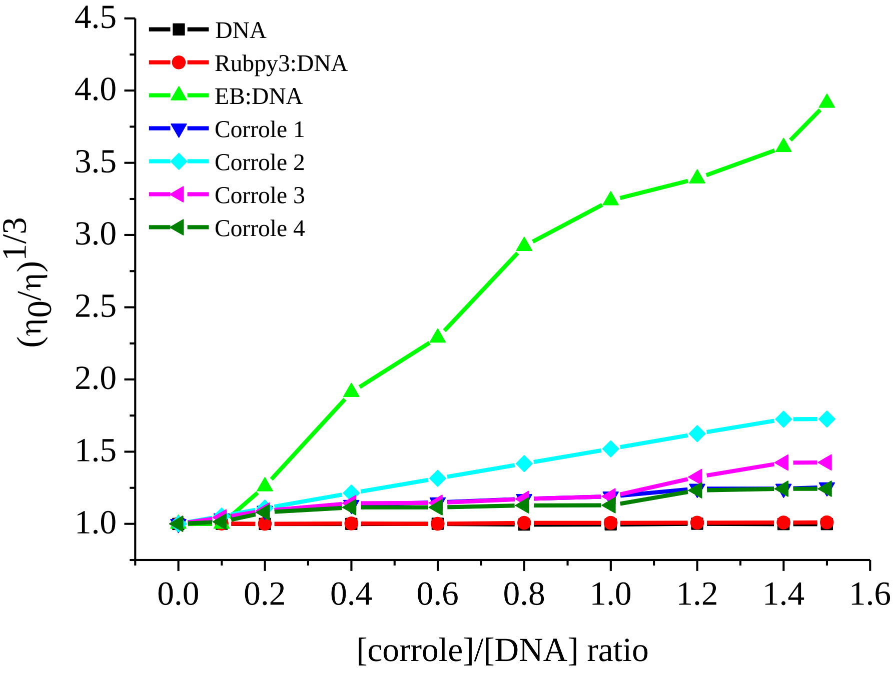

Figure S60. Viscosimetry assay of CT-DNA without and in the presence of corroles **1-4**, in DMSO(5%)/Tris-HCl pH 7.4 mixture buffered solution.

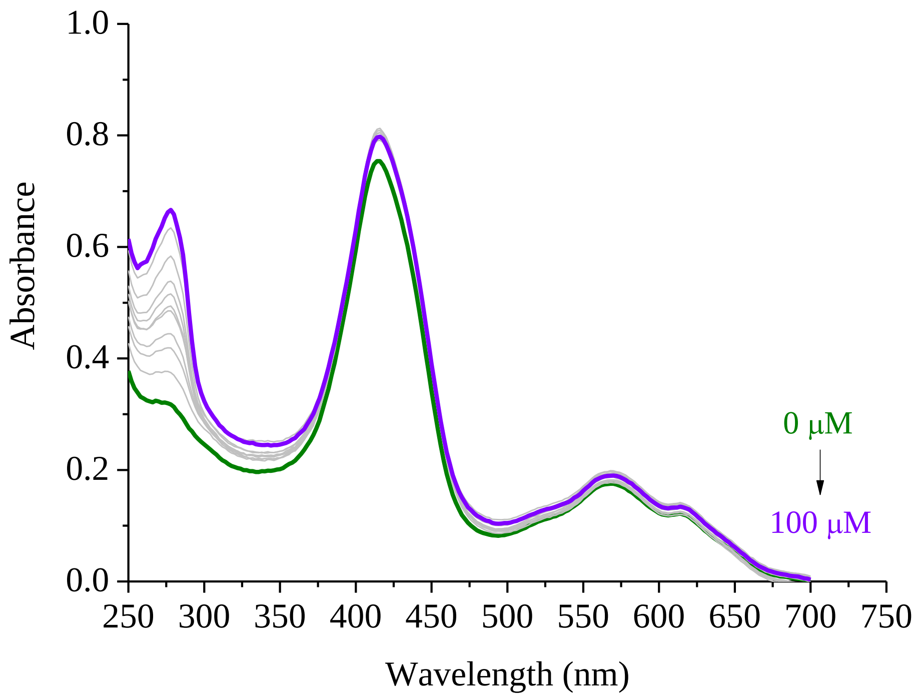

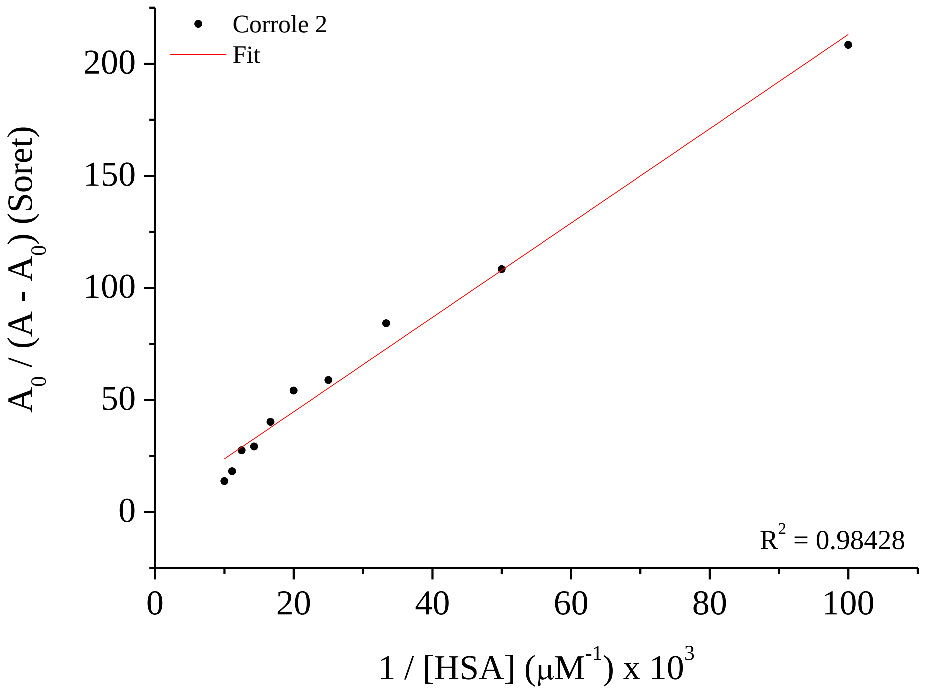

Figure S61. **(up)** UV-Vis spectra of the corrole **2** upon successive additions of HSA concentrations (0 to 100 µM) in DMSO(5%)/Tris-HCl pH 7.4 mixture buffered solution. **(down)** Benesi-Hidelbrandt plots of A_0 / (A – A_0) *versus* 1 / [HSA].

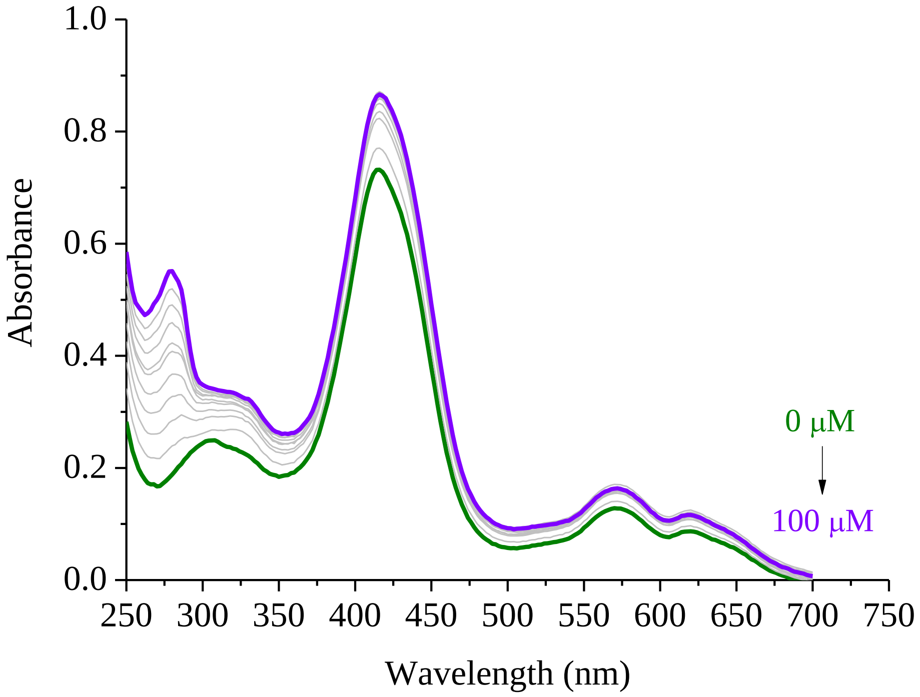

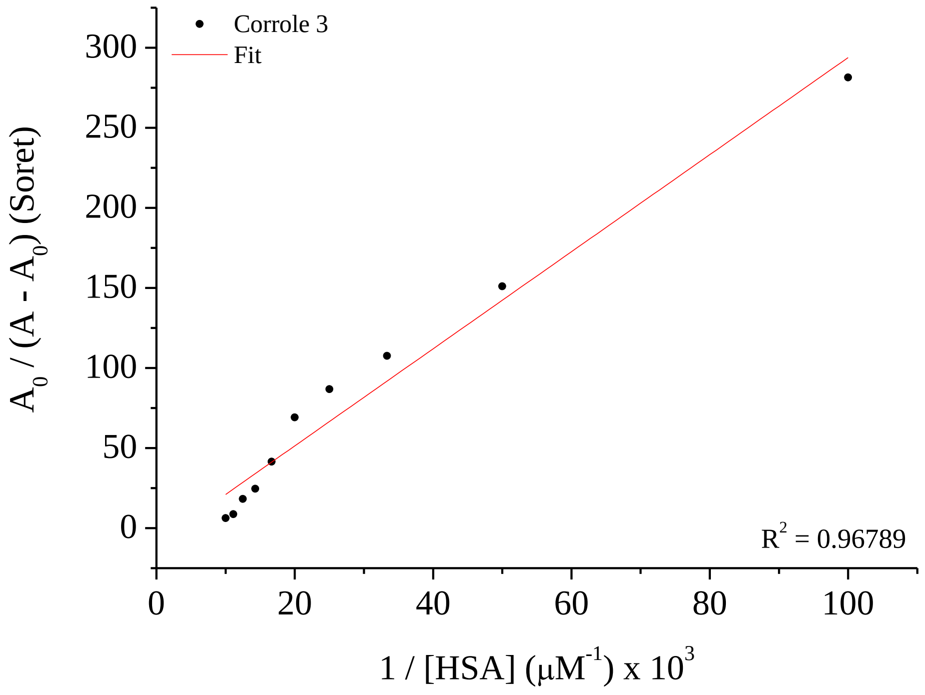

Figure S62. **(up)** UV-Vis spectra of the corrole **3** upon successive additions of HSA concentrations (0 to 100 μM) in DMSO(5%)/Tris-HCl pH 7.4 mixture buffered solution. **(down)** Benesi-Hidelbrandt plots of A_0 / (A – A_0) *versus* 1 / [HSA].

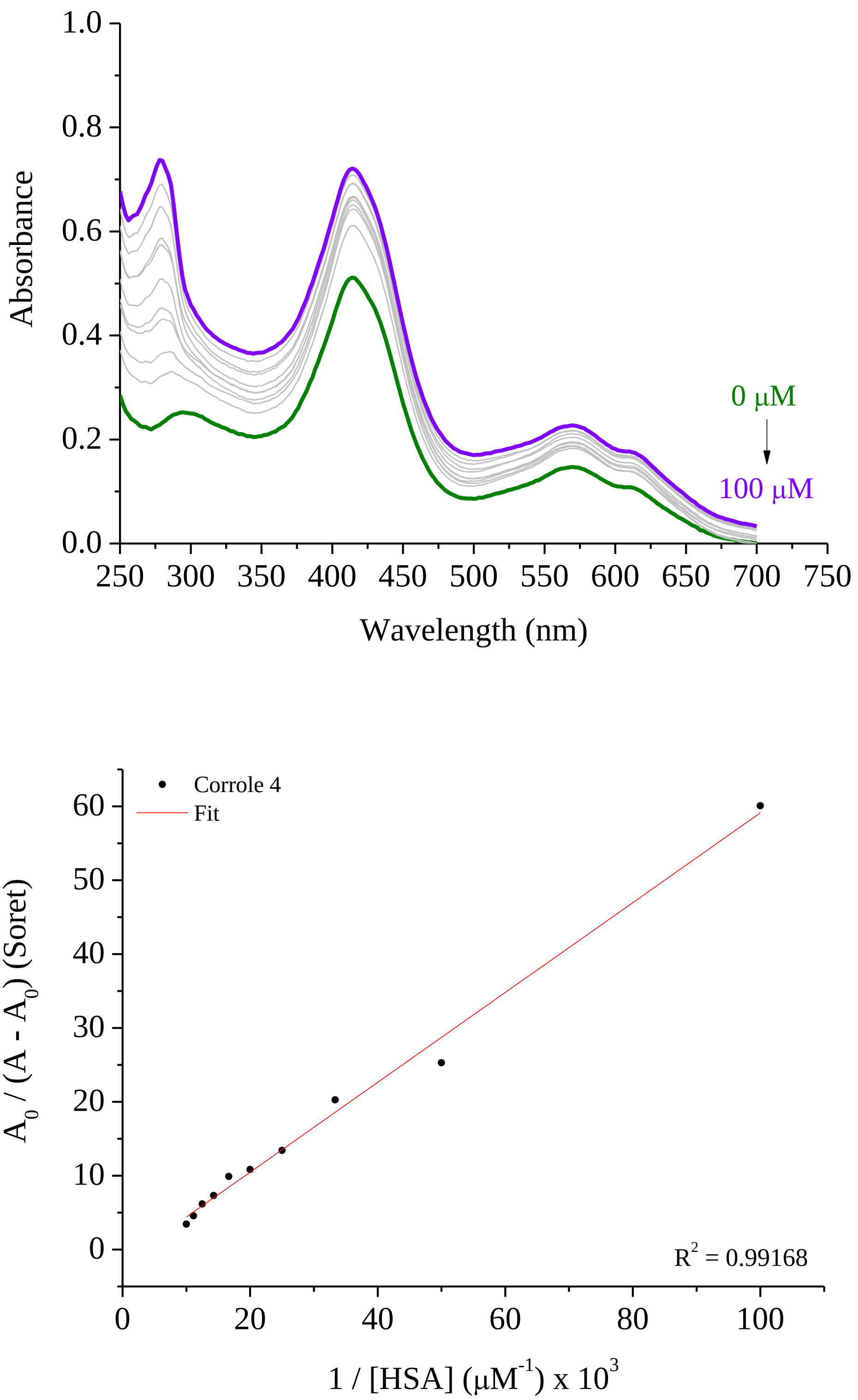

Figure S63. **(up)** UV-Vis spectra of the corrole **4** upon successive additions of HSA concentrations (0 to 100 μM) in DMSO(5%)/Tris-HCl pH 7.4 mixture buffered solution. **(down)** Benesi-Hidelbrandt plots of A_0 / (A – A_0) *versus* 1 / [HSA].

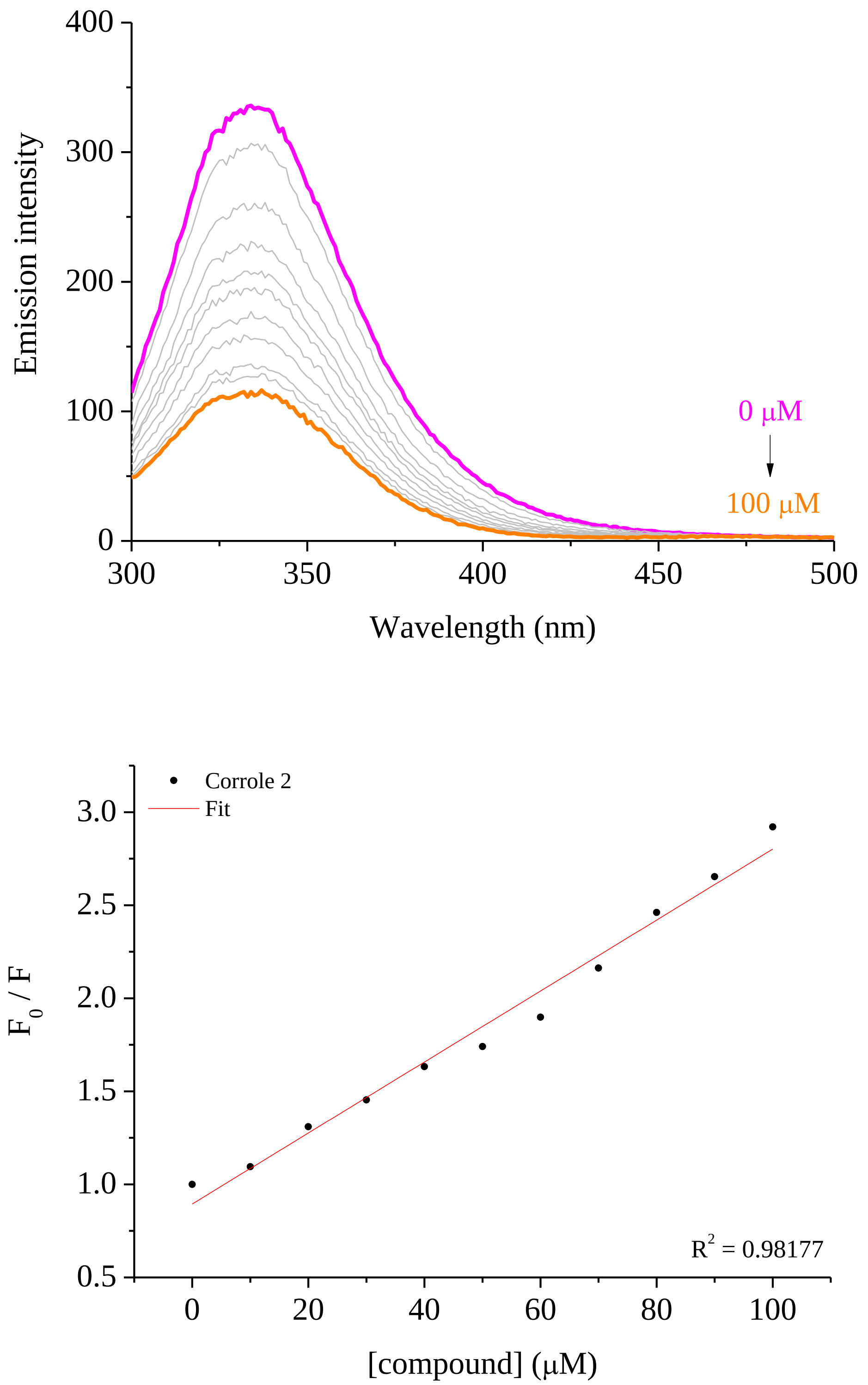

Figure S64. Steady-state fluorescence emission spectra for HSA without and in the presence of corrole **2**, in in DMSO(5%)/Tris-HCl pH 7.4 mixture buffered solution. Graph shows the plot F_0/F *versus* [corrole]. [corrole] = 0–100 μM.

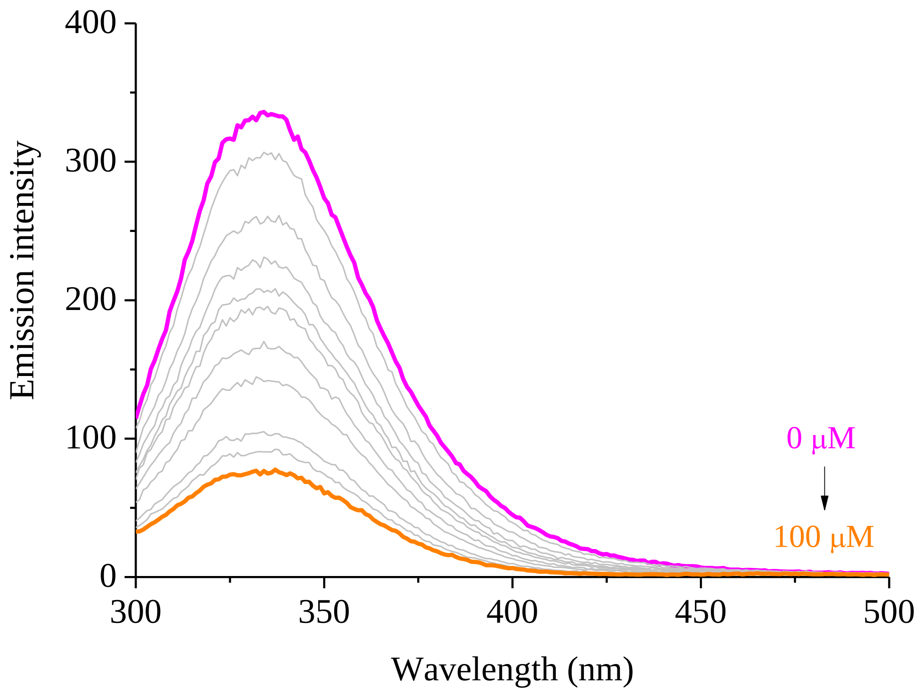

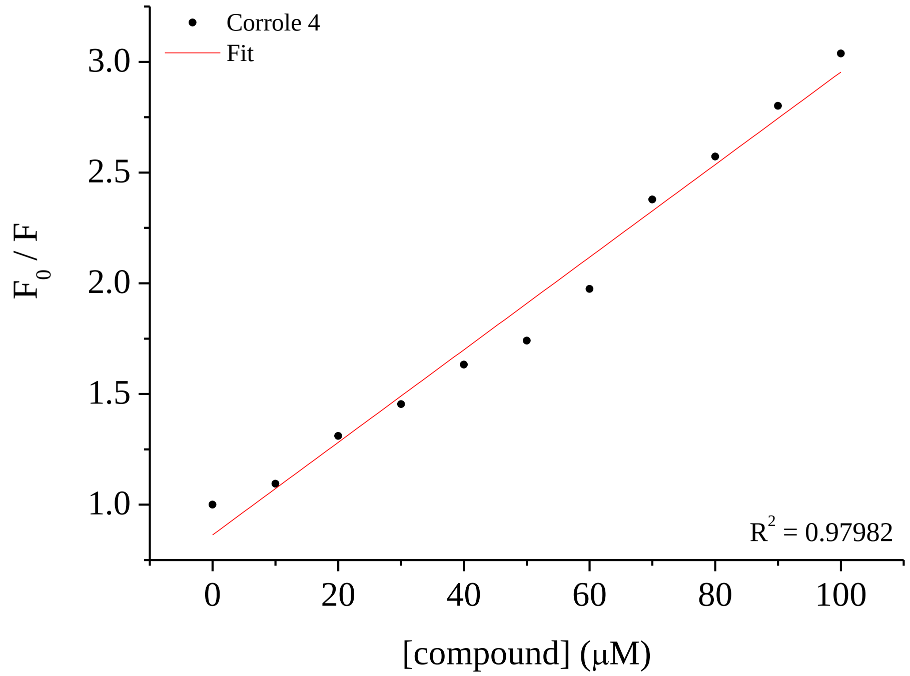

Figure S65. Steady-state fluorescence emission spectra for HSA without and in the presence of corrole **4**, in in DMSO(5%)/Tris-HCl pH 7.4 mixture buffered solution. Graph shows the plot F_0/F *versus* [corrole]. [corrole] = 0–100 μM.

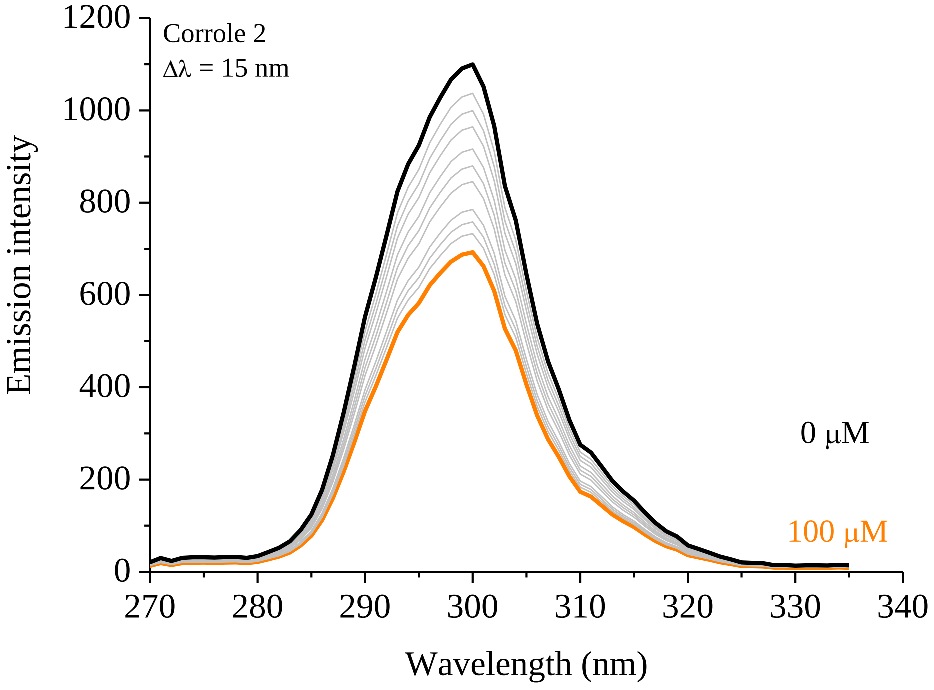

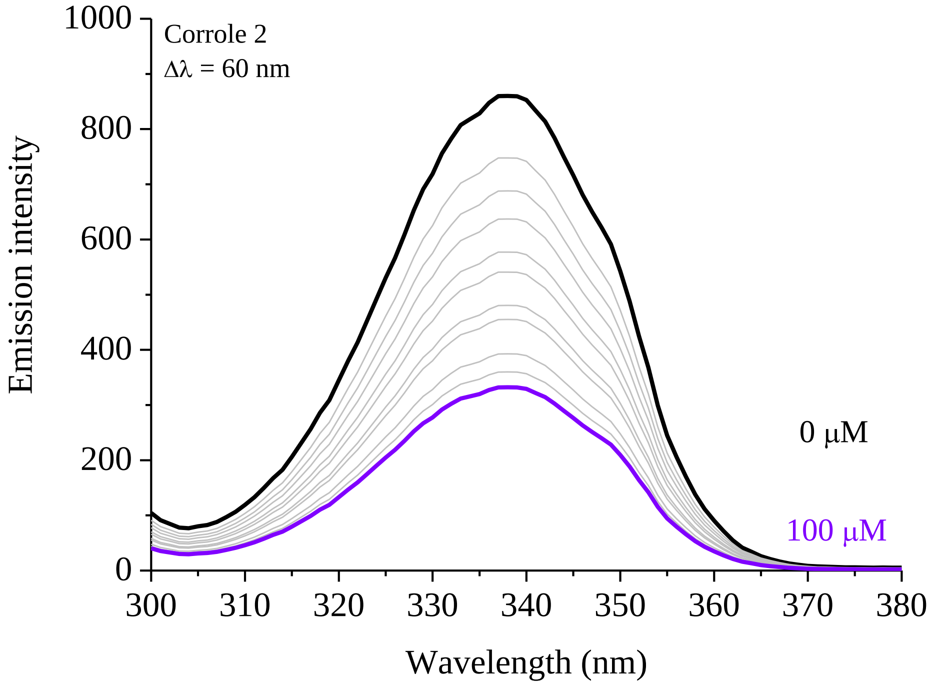

Figure S66. SF fluorescence emission spectra for HSA without and in the presence of corrole **2**, in DMSO(5%)/Tris-HCl pH 7.4 mixture buffered solution, when (up) Δλ = 15 nm and (down) Δλ = 60 nm.

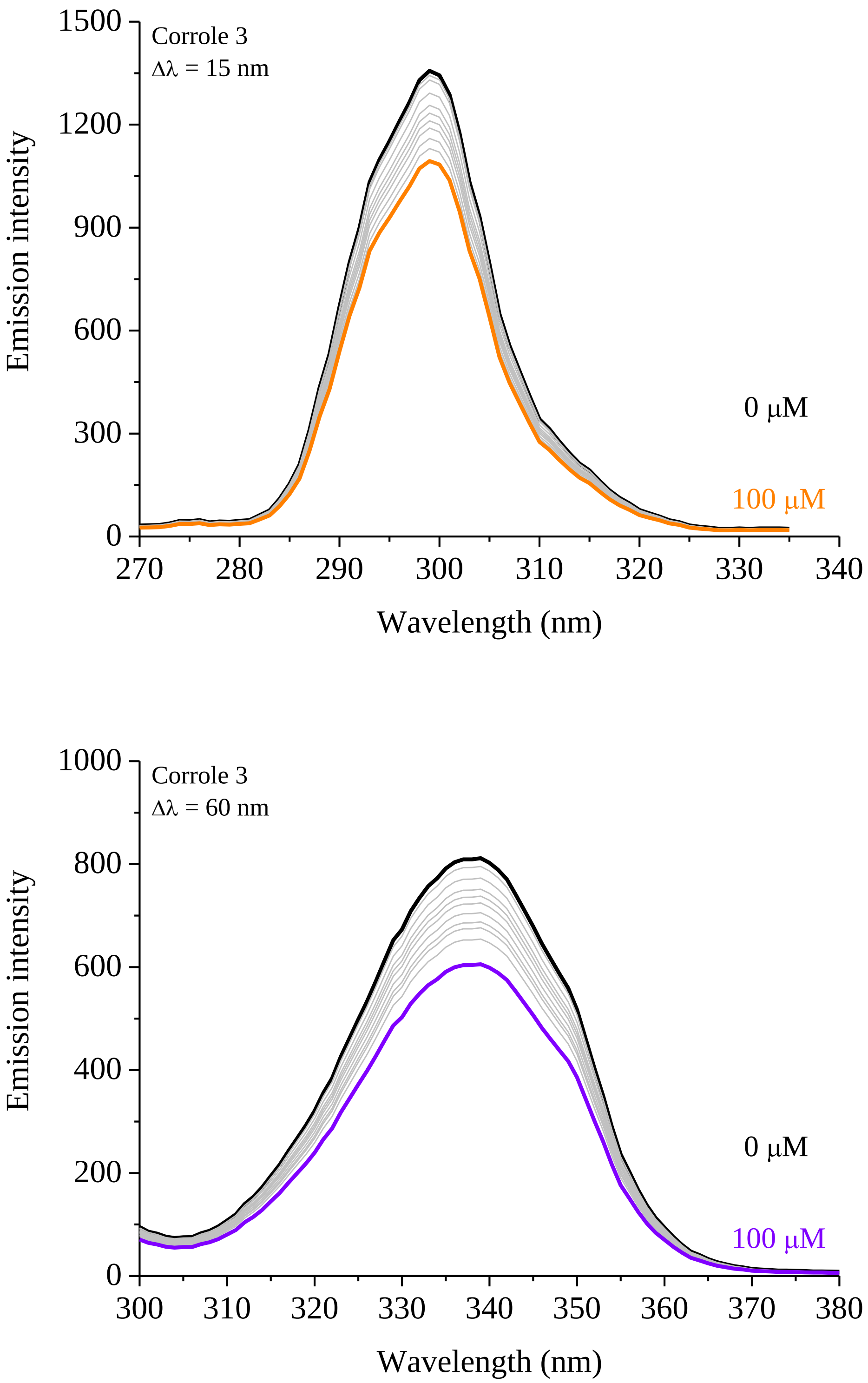

Figure S67. SF fluorescence emission spectra for HSA without and in the presence of corrole **3**, in DMSO(5%)/Tris-HCl pH 7.4 mixture buffered solution, when (up) Δλ = 15 nm and (down) Δλ = 60 nm.

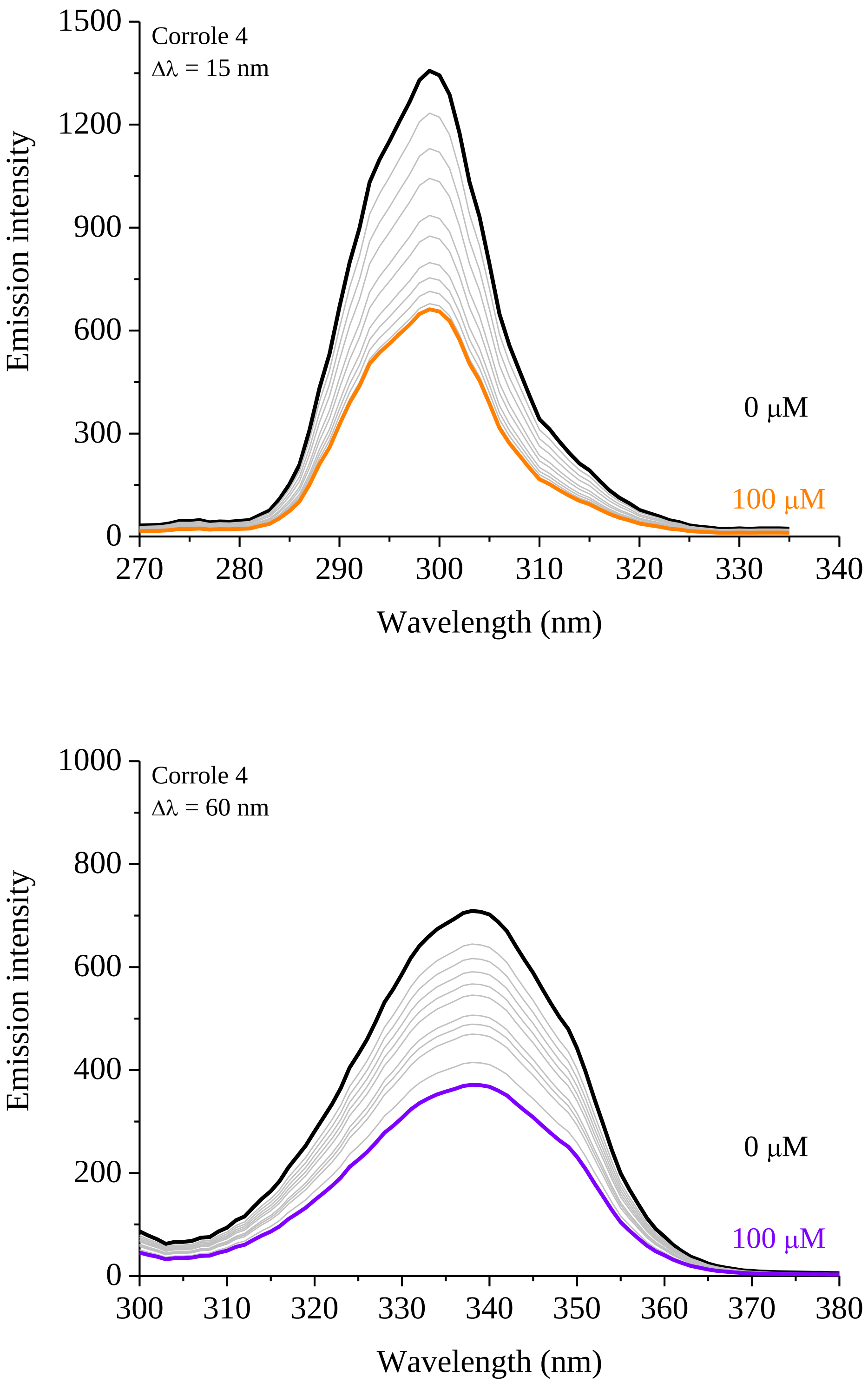

Figure S68. SF fluorescence emission spectra for HSA without and in the presence of corrole **4**, in DMSO(5%)/Tris-HCl pH 7.4 mixture buffered solution, when (up) Δλ = 15 nm and (down) Δλ = 60 nm.

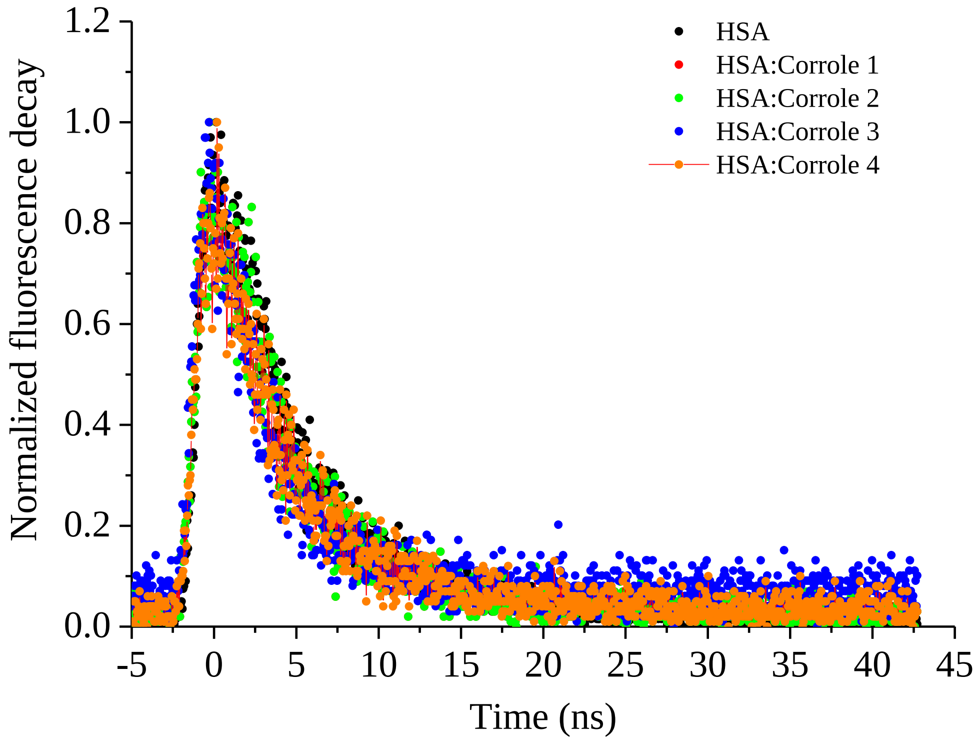

Figure S69. Normalized fluorescence decays of HSA in DMSO(5%)/Tris-HCl pH 7.4 mixture buffered solution, in the absence or in the presence of corroles **1-4**.

Table S1. The absorption wavelengths, λ (nm), and the oscillator strengths, *f*, of the two main peaks of the Soret and Q-bands of the tautomeric state T1, in DMSO solvent, for the studied corrole compounds.

	Soret				Q			
	λ (nm)	*f*	λ (nm)	*f*	λ (nm)	*f*	λ (nm)	*f*
Corrole 1	357.36	1.4144	373.24	1.3183	544.99	0.1032	562.68	0.2926
Corrole 2	358.76	1.4011	375.81	1.3708	547.06	0.1053	564.06	0.2893
Corrole 3	360.69	1.3770	381.29	1.4022	554.05	0.0842	575.62	0.3195
Corrole 4	361.38	1.3800	382.45	1.5170	552.76	0.0793	573.38	0.3105

Table S2. The absorption wavelengths, λ (nm), and the oscillator strengths, *f*, of the two main peaks of the Soret and Q-bands of the tautomeric state T2, in DMSO solvent, for the studied corrole compounds.

	Soret				Q			
	λ (nm)	*f*	λ (nm)	*f*	λ (nm)	*f*	λ (nm)	*f*
Corrole 1	366.45	1.4491	381.47	1.4273	533.03	0.0356	581.29	0.1634
Corrole 2	368.48	1.4580	386.81	1.4265	538.55	0.0527	588.15	0.1885
Corrole 3	370.57	1.4518	390.31	1.4458	543.60	0.0376	596.63	0.2187
Corrole 4	371.36	1.4600	391.58	1.5509	542.74	0.0356	595.04	0.2077

Table S3. Fluorescence decay data of HSA in the absence or in the presence of corroles **1-4**.

Corrole	tf (ns)	X^2
HSA:1	4.89 ± 0.002	1.03680
HSA:2	4.77 ± 0.002	1.17756
HSA:3	4.60 ± 0.002	0.95752
HSA:4	4.60 ± 0.002	1.07678
HSA	5.25 ± 0.001	1.13768

Table S4. Molecular docking results for the interaction between DNA:corroles in the minor groove.

Corrole	Nitrogenated bases	Interaction	Distance (Å)
**Corrole 2**	DG-04	Van der Waals	1.50
	DA-05	Van der Waals	2.30
	DT-20	Van der Waals	3.70
	DC-21	Van der Waals	3.40
	DG-22	Hydrogen bonding	3.20
**Corrole 3**	DG-04	Van der Waals	2.80
	DA-05	Van der Waals	1.70
	DA-06	Hydrogen bonding	1.90
	DT-20	Hydrogen bonding	3.10
	DC-21	Van der Waals	2.00
	DG-22	Hydrogen bonding	3.20
**Corrole 4**	DG-02	Hydrogen bonding	3.60
	DC-23	Van der Waals	3.40
	DG-22	Van der Waals	2.80
	DG-04	Van der Waals	1.40
	DC-21	Van der Waals	3.80
	DA-05	Van der Waals	2.40
	DT-20	Van der Waals	3.30

Table S5. Molecular docking results for the interaction between HSA:corroles for the corresponding main binding site.

Corrole	Amino acid residue	Interaction	Distance (Å)
**Corrole 2**	Lys-195	Hydrogen bonding	2.90
	Lys-199	Van der Waals	1.50
	Trp-214	Van der Waals	2.80
	Arg-222	Hydrogen bonding	3.00
	His-242	Van der Waals	3.10
	Cys-245	Van der Waals	2.80
	Arg-257	Hydrogen bonding	3.70
	Ala-291	Van der Waals	3.40
	Glu-292	Van der Waals	2.20
	Val-343	Van der Waals	2.40

	Arg-114	Hydrogen bonding	3.70
	Leu-115	Van der Waals	3.60
	Arg-117	Van der Waals	1.40
**Corrole 3**	Ile-142	Van der Waals	1.50
**Corrole 4**	His-146	Van der Waals	3.40
	Tyr-161	Hydrogen bonding	2.60
	Leu-182	Van der Waals	2.40
	Arg-186	Van der Waals	3.20
	Lys-190	Van der Waals	2.70
